# Dynamic nanodomains dictate macroscopic properties in lead halide perovskites

**DOI:** 10.1038/s41565-025-01917-0

**Published:** 2025-06-02

**Authors:** Milos Dubajic, James R. Neilson, Johan Klarbring, Xia Liang, Stephanie A. Bird, Kirrily C. Rule, Josie E. Auckett, Thomas A. Selby, Ganbaatar Tumen-Ulzii, Yang Lu, Young-Kwang Jung, Cullen Chosy, Zimu Wei, Yorrick Boeije, Martin v. Zimmermann, Andreas Pusch, Leilei Gu, Xuguang Jia, Qiyuan Wu, Julia C. Trowbridge, Eve M. Mozur, Arianna Minelli, Nikolaj Roth, Kieran W. P. Orr, Arman Mahboubi Soufiani, Simon Kahmann, Irina Kabakova, Jianning Ding, Tom Wu, Gavin J. Conibeer, Stephen P. Bremner, Michael P. Nielsen, Aron Walsh, Samuel D. Stranks

**Affiliations:** 1 Department of Chemical Engineering and Biotechnology, University of Cambridge, Cambridge, UK; 2 School of Photovoltaic & Renewable Engineering, UNSW Sydney, Kensington, New South Wales Australia; 3 Department of Chemistry, Colorado State University, Fort Collins, CO USA; 4 School of Materials Science & Engineering, Colorado State University, Fort Collins, CO USA; 5 Inorganic Chemistry Laboratory, University of Oxford, Oxford, UK; 6 Department of Materials, Imperial College London, London, United Kingdom; 7 Department of Physics, Chemistry and Biology (IFM), Linköping University, Linköping, Sweden; 8 Australian Synchrotron, Australian Nuclear Science and Technology Organisation, Clayton, Victoria Australia; 9 Australian Nuclear Science and Technology Organisation, Kirrawee, New South Wales Australia; 10 Department of Physics, Cavendish Laboratory, University of Cambridge, Cambridge, UK; 11 Deutsches Elektronen-Synchrotron DESY, Hamburg, Germany; 12 Taizhou Institute of Science and Technology, Nanjing University of Science and Technology, Taizhou, China; 13 School of Microelectronics and Control Engineering, Jiangsu Province Cultivation Base for State Key Laboratory of Photovoltaic Science and Technology, Jiangsu Collaborative Innovation Center of Photovoltaic Science and Engineering, Changzhou University, Changzhou, China; 14 Faculty of Engineering and IT, University Technology Sydney, Sydney, Australia; 15 Helmholtz-Zentrum Berlin für Materialien und Energie GmbH, Solar Energy Division, Berlin, Germany; 16 School of Mathematical and Physical Sciences, University Technology Sydney, Sydney, Australia; 17 Institute of Technology for Carbon Neutralization, Yangzhou University, Yangzhou, China; 18 School of Materials Science and Engineering, Faculty of Science, University of New South Wales, UNSW Sydney, Kensington, New South Wales Australia; 19 Department of Applied Physics, The Hong Kong Polytechnic University, Kowloon, China; 20 Present Address: Department of Materials Science and Engineering, Stanford University, Stanford, CA USA

**Keywords:** Solar cells, Phase transitions and critical phenomena

## Abstract

Lead halide perovskites have emerged as promising materials for solar energy conversion and X-ray detection owing to their remarkable optoelectronic properties. However, the microscopic origins of their superior performance remain unclear. Here we show that low-symmetry dynamic nanodomains present in the high-symmetry average cubic phases, whose characteristics are dictated by the A-site cation, govern the macroscopic behaviour. We combine X-ray diffuse scattering, inelastic neutron spectroscopy, hyperspectral photoluminescence microscopy and machine-learning-assisted molecular dynamics simulations to directly correlate local nanoscale dynamics with macroscopic optoelectronic response. Our approach reveals that methylammonium-based perovskites form densely packed, anisotropic dynamic nanodomains with out-of-phase octahedral tilting, whereas formamidinium-based systems develop sparse, isotropic, spherical nanodomains with in-phase tilting, even when crystallography reveals cubic symmetry on average. We demonstrate that these sparsely distributed isotropic nanodomains present in formamidinium-based systems reduce electronic dynamic disorder, resulting in a beneficial optoelectronic response, thereby enhancing the performance of formamidinium-based lead halide perovskite devices. By elucidating the influence of the A-site cation on local dynamic nanodomains, and consequently, on the macroscopic properties, we propose leveraging this relationship to engineer the optoelectronic response of these materials, propelling further advancements in perovskite-based photovoltaics, optoelectronics and X-ray imaging.

## Abstract (web summary)

Dynamic nanodomains in lead halide perovskites, dictated by A-site cations, crucially affect the optoelectronic properties by modulating electronic disorder and consequently enabling better solar cells and optoelectronic devices.

## Main

Lead halide perovskites (LHPs), with their extraordinary optoelectronic properties, have emerged as a promising class of materials for light emission, detection and energy conversion^1–4^. Recent advancements in perovskite solar cells have led to record-breaking efficiencies primarily by using formamidinium (FA) cations in perovskite polycrystalline films^5,6^. Similarly, FAPbBr_3_ single crystals outperform MAPbBr_3_ in X-ray detection, exhibiting lower dark currents, enhanced thermal and phase stabilities, fivefold longer charge-carrier lifetimes and fourfold greater diffusion lengths^7,8^. This pronounced sensitivity to the choice of A-site cation is perplexing given that conventional understanding suggests that the electronic structure, and consequently the device performance, should not be substantially influenced by such changes.

In many solids, short-range correlations of atomic species can give rise to a local crystallographic structure that differs from the global, average structure and can critically define the macroscopic properties. Hidden local order is known to modify the electronic and phononic band structures^9^, and has been linked to unexplained macroscopic properties in various materials classes, including certain battery materials^10,11^, relaxor ferroelectrics^12^ and superconductors^13^. In the cubic perovskite aristotype, local atomic correlations, such as B-site off-centring, octahedral tilt correlations and A-site correlations, can lead to diverse local structures (Supplementary Fig. 2a). The real space description of the local structure in these materials is still debated and ranges from polymorphous networks^14^ and B-site off-centring correlations^15^ to local orthorhombic phases embedded in average tetragonal and cubic global structures^16,17^, static in-phase and anti-phase octahedral correlations^18^, non-centrosymmetric dynamic local domains^19^ and two-dimensional dynamic octahedral sheets^20,21^. Although simulations suggest that such a hidden local order can substantially influence the optoelectronic performance and ion migration in LHPs^21–24^, direct experimental evidence has remained elusive.

In this paper, we use single-crystal diffuse scattering and inelastic neutron spectroscopy to directly probe the local structure of MAPbBr_3_ and FAPbBr_3_ in reciprocal space. We develop a phenomenological model and run large-scale machine learning molecular dynamics (MD) simulations^25,26^ that fully reproduce all the experimental features in the diffuse scattering patterns. This approach allows us to achieve a comprehensive understanding of the local structure in real space, which manifests in the presence of dynamic nanodomains. We can accurately quantify their spatial dimensions, assign local symmetry and estimate their density within the material, as well as rule out certain local structural configurations previously proposed in the literature. We uncover that the properties of dynamically tilted octahedral pockets, which form transient twinned nanodomains and constitute the local structure, are dictated by the A-site cation. Specifically, methylammonium (MA) cations promote densely packed planar nanodomains with out-of-phase octahedral tilting, whereas FA favours sparse spherical nanodomains with in-phase octahedral tilting. Hyperspectral photoluminescence (PL) microscopy experiments supported by simulations provide the first direct experimental evidence linking the A-site-cation-driven local structure to the tunability of the macroscopic properties in LHPs. We demonstrate that the spherical nanodomains in FA hybrid LHPs lead to superior macroscopic optoelectronic performance of FA over their MA analogues. The control over local order could be leveraged to engineer enhanced optoelectronic properties, potentially driving further advancements in perovskite-based photovoltaics, optoelectronics and X-ray imaging and even other, more exotic device types.

## A-site cations dictate local octahedral correlations

**Fig. 1:**
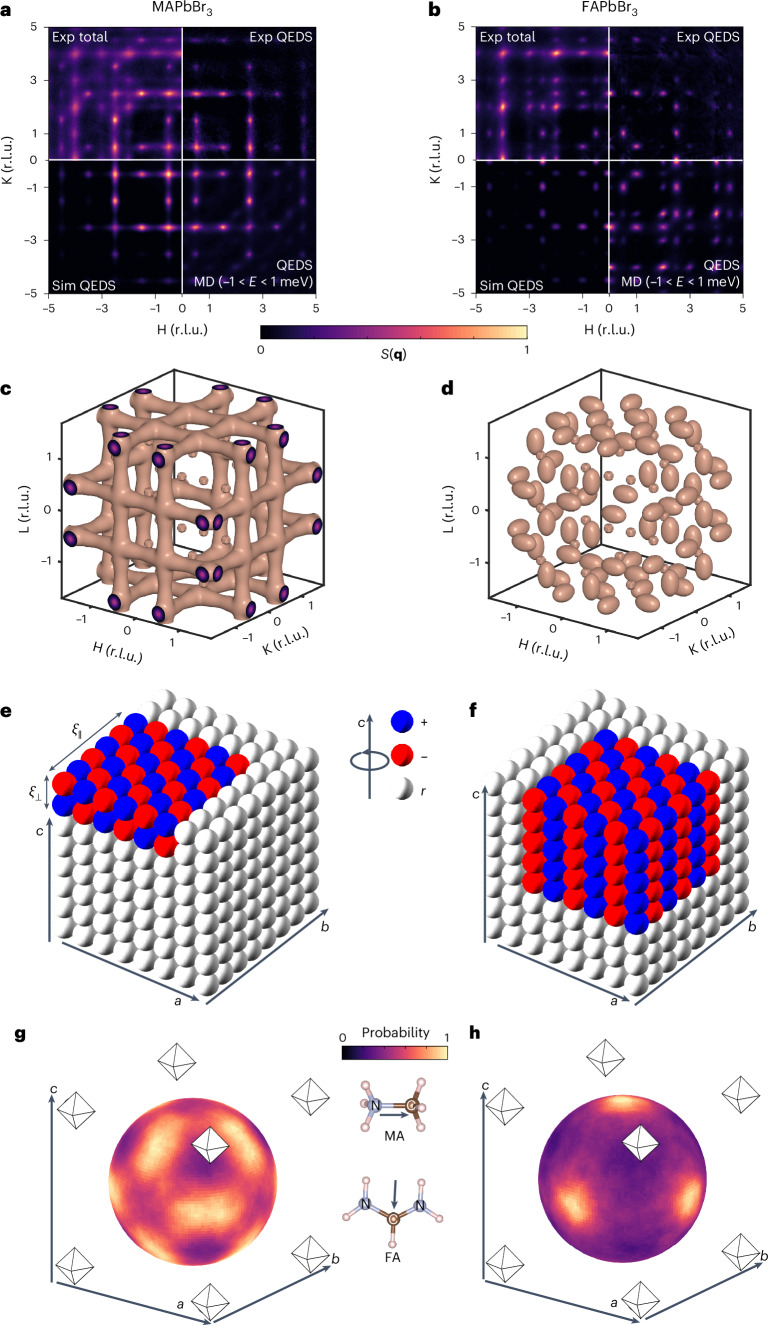
X-ray diffuse scattering reveals the striking contrast in symmetry, shape and distribution of nanodomains of locally tilted octahedra in MAPbBr_3_ and FAPbBr_3_. **a**,**b**, Experimental (Exp; 300 K) and simulated (Sim) X-ray scattering function *S*(**q**) at the (H, K, L = 1.5) reciprocal space plane in the average cubic $Pm\bar{3}m$ phase of MAPbBr_3_ (**a**) and FAPbBr_3_ (**b**). The figures are divided into four quadrants: top left, total experimental *S*(**q**); top right, experimentally derived QEDS; bottom left, simulated QEDS obtained by fitting the experimentally derived QEDS data to a phenomenological model; bottom right, MD-simulated *S*(**q**, *E*) integrated over the QEDS energy window, that is, –1 meV < *E* < 1 meV. *S*(**q**) intensities in each quadrant are normalized to 1, with the two-dimensional images coloured via bilinear interpolation. MD trajectories simulated at 340 K for MAPbBr_3_ and 250 K for FAPbBr_3_ were chosen as they best match the experimental 300 K data due to an offset between the experimentally and MD-simulated phase transition temperatures (Supplementary Note 8). **c**,**d**, Isosurface representations of 3D QEDS at 300 K in MAPbBr_3_ (**c**) and FAPbBr_3_ (**d**) underscore the composition-dependent differences. r.l.u., reciprocal lattice units. **e**,**f**, Local octahedral correlations at a single snapshot in time inferred from the experimentally derived QEDS in MAPbBr_3_ (**e**) and FAPbBr_3_ (**f**). Each octahedron is represented by a sphere in which the colour represents the respective tilt angle along the *c* crystallographic direction, with blue corresponding to positive; red, to negative; and white, to a random close-to-zero tilt angle. Local octahedral tilts in MAPbBr_3_ (**e**) form planar/pancake-like correlations, with the octahedra correlated out-of-phase in the *c* direction, whereas in FAPbBr_3_, correlations are more isotropic, forming spherical nanodomains of octahedra that are correlated in-phase along the *c* direction. Nanodomain sizes are defined by correlation lengths *ξ*_⊥_ and *ξ*_∥_, as depicted in **e**. **g**,**h**, Coloured spheres for MAPbBr_3_ (**g**) and FAPbBr_3_ (**h**) depict the spatial distributions of the electric polarization vector probability density of A-site cations, as derived from the MD trajectories. The A-site cation, and consequently the associated probability density spheres, occupy the space within the cuboctahedral cavity defined by the inorganic octahedra, as shown. The insets illustrate the orientation of the mapped vectors (with arrows) relative to the geometry of the MA and FA molecules.

To explore the impact of the organic A-site cation on the local structure in LHPs, we performed X-ray diffuse scattering on MAPbBr_3_ and FAPbBr_3_ single crystals to cover a large portion of reciprocal space. Diffuse scattering arises when X-rays scatter from atoms that dynamically or statically deviate from perfect periodicity and still maintain some degree of spatial correlation within the crystal. We present the experimental X-ray scattering function *S*(**q**) across (H, K, L = 1.5) reciprocal planes of MAPbBr_3_ and FAPbBr_3_ (Fig. 1a,b, top-left quadrants), respectively, for nominally cubic ($Pm\bar{3}m$) phases at room temperature. The observed diffuse scattering indicates the presence of local spatial correlations that form twinned nanodomains in these materials.

We conducted MD simulations using machine learning potentials on 20^3^ pseudo-cubic unit cells (Methods and ref. ^25^). Since inelastic and elastic X-ray scattering events are indistinguishable in the present measurements, the experimental *S*(**q**) is inherently energy integrated. We, therefore, computed *S*(**q**, *E*) at (H, K, L = 1.5) planes from the MD trajectories and selectively integrated it over different energy ranges to separate it into distinct components. Full-energy-range integration yielded *S*(**q**), demonstrating excellent agreement with the experiment (Supplementary Note 6). Quasi-elastic diffuse scattering (QEDS) arises from nearly elastic scattering events with energy transfers below 1 meV and this MD-derived QEDS features broad diffuse peaks at the R high-symmetry points in MAPbBr_3_ and at the X and M points in FAPbBr_3_ (Fig. 1a,b (bottom-right quadrant), respectively). By contrast, integrating over 1 meV < *E* < 10 meV captures thermal diffuse scattering (TDS), which, in general, is widely distributed across reciprocal space and is here pronounced at the X points (Supplementary Fig. 18). Details about the symmetry points in the Brillouin zone are provided in Supplementary Fig. 15.

We separated the total experimental diffuse scattering into QEDS (Fig. 1a,b, top-right quadrants) and TDS components following the procedure detailed in Supplementary Note 7. In MAPbBr_3_, the experimental QEDS matches the MD-derived pattern (root mean squared error analysis is given in Supplementary Fig. 14). In FAPbBr_3_, they mostly agree, except for the extra intensity at the X point in the MD QEDS, attributed to the zone-centre acoustic phonons (Supplementary Note 6b). Further, we will show that the QEDS at the R points in MAPbBr_3_ and the M points in FAPbBr_3_ arises from anharmonic lattice contributions^20,27,28^, which generate low-energy excitations of dynamic, locally tilted octahedral nanodomains that scatter X-rays quasi-elastically.

To quantify the nanodomain size, shape and symmetry from experimental QEDS, we developed a phenomenological local octahedra tilting model. In this approach, we calculate the X-ray structure factors for a cubic lattice containing octahedrally tilted nanodomains and broaden each three-dimensional (3D) Bragg peak in inverse proportion to the 3D correlation lengths, which are used as fitting parameters (Supplementary Note 5a). The model accurately reproduces the experimental QEDS (compare the top-right and bottom-left quadrants in Fig. 1a,b) as well as replicates the QEDS features in other reciprocal space planes (Supplementary Note 5c).

The QEDS patterns of MAPbBr_3_ and FAPbBr_3_ exhibit distinct characteristics. In MAPbBr_3_, rod-like 3D QEDS in reciprocal space (Fig. 1c) indicates planar (anisotropic) local nanodomains in real space with out-of-phase tilted octahedra akin to the tetragonal *I*4/*mcm* symmetry (Fig. 1e). Fitting to a phenomenological model yields in-plane diameters of *ξ*_∥_ ≈ 21 Å and out-of-plane diameters of *ξ*_⊥_ ≈ 6 Å (Fig. 1e). By contrast, FAPbBr_3_ exhibits ellipsoidal QEDS (Fig. 1d), reflecting a more isotropic local structure in real space, with spherical nanodomains having in-phase tilted octahedra akin to the *P*4/*mbm* symmetry, where *ξ*_∥_ ≈ 21 Å and *ξ*_⊥_ ≈ 14 Å (Fig. 1f). This difference is consistent with recent predictions of distinct dynamic disorder in FAPbBr_3_ and MAPbBr_3_ (ref. ^29^). Real and reciprocal space analyses of the MD trajectories confirm these findings (Supplementary Note 8). Volumetric density estimates from the MD data show that MAPbBr_3_ contains roughly twice as many dynamic nanodomains as FAPbBr_3_ (Supplementary Fig. 23). This observation aligns with both experimental and MD-simulated *S*(**q**) data, which show that the intensity of TDS relative to the QEDS signals in FAPbBr_3_ is markedly higher than in MAPbBr_3_. Further details on how TDS and QEDS relate to the density of dynamic nanodomains are given in Supplementary Note 7.

The contrasting nanodomain morphologies can be understood to originate from the complex interplay between an anharmonic potential energy surface (governing the octahedral tilt modes) and dynamic hydrogen bonds. Anharmonic interactions lead to strong in-plane correlations of octahedral tilt rotations but only weak out-of-plane correlations, resulting in default planar dynamic nanodomains^30^. In hybrid LHPs, however, the stochastically reorienting organic cations enhance the out-of-plane coupling through dynamic hydrogen bonding with the inorganic framework. The directionality of hydrogen bonds can be inferred from MD trajectories by computing spatially and temporally averaged probabilities of the electric polarization vector orientation in the MA and FA molecules (Fig. 1g,h, respectively). In FA, the vector shows strong preferential orientations along the 〈100〉 directions, whereas MA vectors adopt a broader range of orientations, with the 〈100〉 and 〈111〉 directions being prohibited. Pair distribution function analysis of the same trajectories further indicates that FA forms stronger hydrogen bonds with bromine compared with MA (Supplementary Fig. 19). These findings suggest that the isotropic (spherical) dynamic nanodomains in FAPbBr_3_ arise from the robust and highly directional coupling of FA to the inorganic cage, facilitating extended out-of-plane correlation lengths. By contrast, the weaker hydrogen bonding and less preferential orientation of the A-site cation in MAPbBr_3_ lead to weaker out-of-plane coupling, resulting in the presence of only default, anharmonically driven planar nanodomains.

### Local dynamic nanodomains are twinned

**Fig. 2:**
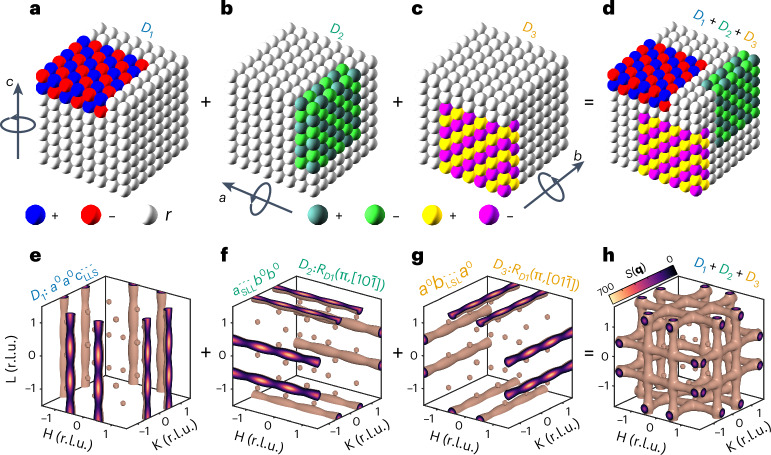
Phenomenological X-ray diffuse scattering model reveals three types of locally tilted octahedral nanodomains in cubic MAPbBr_3_. **a**, *D*_1_ local nanodomain component in real space consists of correlated octahedral tilts, with short-range anti-phase ordering of *c*-axis tilts (one *I*4/*mcm* unit of *c* axis) along *c* and out-of-phase long-range ordering of *c*-axis tilts along *a* and *b*, denoted as ${a}^{0}{a}^{0}{c}_{{\rm{LLS}}}^{---}$ in modified Glazer notation (Supplementary Note 4 provides details about the notation). Note that when the octahedra are tilted along the *c* axis, these tilts are always out-of-phase correlated along *a* and *b* due to rigid octahedra connection rules. **b**, *D*_2_ nanodomain component in real space consists of octahedra that exhibit short-range anti-phase ordering of *a*-axis tilts along *a* and long-range ordering of *a*-axis tilts along *b* and *c* denoted as ${a}_{{\rm{SLL}}}^{---}{b}^{0}{b}^{0}$. **c**, *D*_3_ nanodomain component in real space consists of octahedra that exhibit short-range anti-phase ordering of *b*-axis tilts along *b* and long-range ordering of *b*-axis tilts along *a* and *c* denoted as ${a}^{0}{b}_{{\rm{LSL}}}^{---}{a}^{0}$. Short-range and long-range correlation lengths are equivalent to out-of-plane and in-plane correlation lengths, respectively, in Fig. 1. Each octahedron is represented by a sphere, where the colour represents the octahedron tilt angle along the *c* (**a**), *a* (**b**) and *b* (**c**) crystallographic directions. Colours represent positive/negative tilts, whereas white corresponds to a random (close-to-zero) tilt angle. **e**–**g**, *S*(**q**) QEDS isosurfaces computed from real space nanodomains *D*_1_ (**e**), *D*_2_ (**f**) and *D*_3_ (**g**). **f**, Reciprocal space of the second local structure component (*D*_2_) is derived by rotating *D*_1_ in **a** by 180° around the $[10\bar{1}]$ vector of the *D*_1_ coordinate system, which is, for this particular crystal symmetry, equivalent to 90° rotation around [010]. **g**, Reciprocal space of the third local structure component (*D*_3_) is achieved by rotating *D*_1_ in **a** by 180° around the $[01\bar{1}]$ vector of the *D*_1_ coordinate system, (equivalent to 90° rotation around [100]). **h**, Experimentally observed *S*(**q**) QEDS isosurface is the cumulative sum of all the three components. As shown in **d**, in real space, the local structure is characterized as a combination of three distinct local *I*4/*mcm* planar nanodomains that are mutually orthogonal, that is, twinned.

Utilizing our phenomenological model, we show that local dynamic nanodomains exhibit twinning. The QEDS patterns in the cubic phases are modelled as the sum of three non-merohedrally twinned components of the local *I*4/*mcm* (MAPbBr_3_) and *P*4/*mbm* (FAPbBr_3_) nanodomains. These components utilize the same rotation operators that define the global twins emerging during the ferroelastic cubic to tetragonal phase transition in MAPbBr_3_. We will demonstrate this in the example of MAPbBr_3_. The local twinning rules are analogous in FAPbBr_3_ and are shown in Supplementary Fig. 5. In Fig. 2a, we present the kernel twin component *D*_1_ of the local tetragonal *I*4/*mcm* phase in MAPbBr_3_ (Fig. 1g). The resulting *S*(**q**) isosurface of the local structure component *D*_1_ is shown in Fig. 2e. The diffuse scattering rods align with the *L* reciprocal space direction (*c* direction in real space), due to the short-range *c*-axis tilt correlations along the same direction. Similarly, after applying the relevant symmetry operations, we can derive the *S*(**q**) isosurfaces of *D*_2_ and *D*_3_ components (Fig. 2f,g, respectively). The experimental QEDS is a sum of the three components (Fig. 2h). Analogously, real space local structure is a sum of the real space components (Fig. 2d). Thus, the local structure consists of mutually orthogonal (twinned) nanodomains of locally tilted octahedra embedded in the nearly cubic matrix, as depicted in Fig. 2d for MAPbBr_3_ and Supplementary Fig. 5 for FAPbBr_3_. The same conclusion is drawn from the real space analysis of MD trajectories (Supplementary Note 8). Although previously not explicitly discussed in the literature, this phenomenon also occurs in MAPbI_3_ (ref. ^21^) and CsPbBr_3_ (ref. ^20^), and is likely universal across various halide perovskite compositions.

## Probing spatiotemporal local structure correlations

**Fig. 3:**
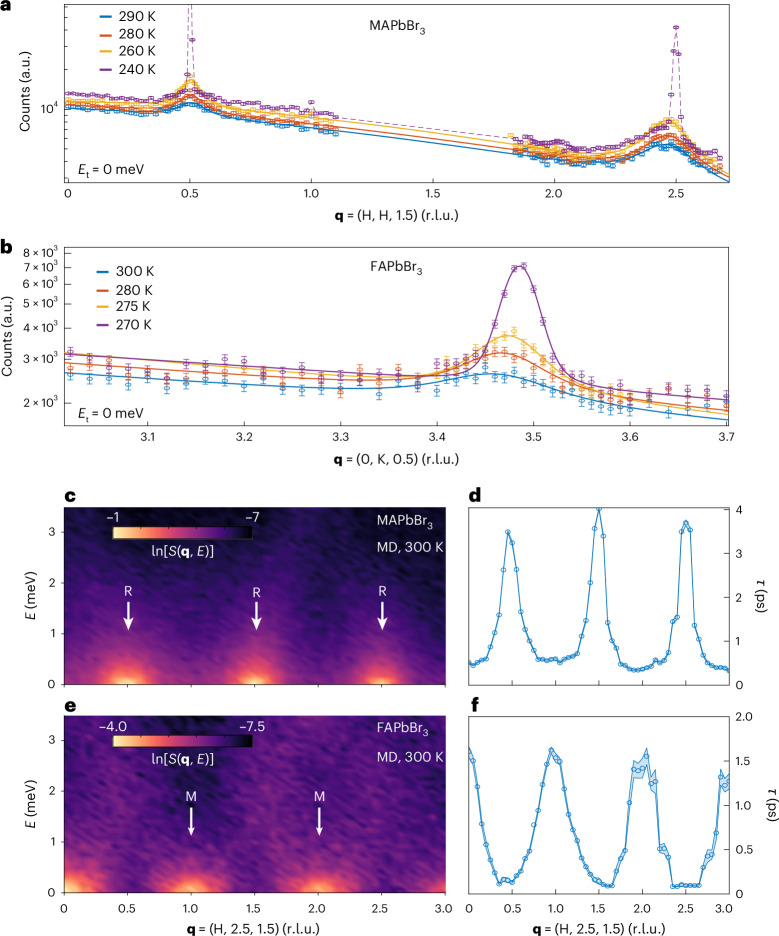
Elastic neutron scattering and MD simulations confirm the presence of quasi-elastic dynamic local octahedral nanodomains within cubic MAPbBr_3_ and FAPbBr_3_. **a**, Elastic scans with Taipan (zero neutron energy transfer, *E*_t_ = 0) across the [H, H, 1.5] direction in the reciprocal space of MAPbBr_3_ for various temperatures in the average cubic phase. **b**, Elastic scans with Taipan across the [0, K, 0.5] direction in the reciprocal space of FAPbBr_3_ for various temperatures in the average cubic phase. The experimental data in **a** and **b** are presented as $n\pm \sqrt{n}$, where $n$ is the number of detected neutrons and the error bars represent the Poisson standard deviation $\sqrt{n}$. The incoherent elastic scattering background was modelled using a Gaussian centred at **q** = 0, and coherent quasi-elastic signals with Gaussians centred at the M or R points, in an attempt to extract the corresponding correlation lengths (Supplementary Fig. 39). **c**, MD-computed *S*(**q**, *E*) for MAPbBr_3_ at 300 K for **q** = (H, 2.5, 1.5), which corresponds to a horizontal cut of the (H, K, L = 1.5) plane at K = 2.5. **q** with integer/non-integer H values correspond to *M*/*R* points in the Brillouin zone. Each vertical cross-section of **c** manifests as a quasi-elastic Lorentzian, centred at zero energy transfer. In **d**, the lifetimes associated with these quasi-elastic Lorentzian lines at every **q** are derived. The shaded area around the data points represents 95% confidence intervals of the performed fits. Panels **e** and **f** show the analogous plots for FAPbBr_3_ MD-computed *S*(**q**, *E*) at 300 K.

We used inelastic neutron spectroscopy with the Sika (cold) and Taipan (thermal) triple-axis spectrometers, to probe the local structural dynamics. By energy filtering the scattered neutrons, we measured the QEDS and observed peaks at the *R* points in MAPbBr_3_ (Fig. 3a) and the *M* points in FAPbBr_3_ (Fig. 3b) over a range of temperatures in the average cubic phase. Sika’s higher energy resolution confirmed the same QEDS signals (Supplementary Fig. 40). Although background elastic incoherent scattering from the A-site hydrogen atoms prevents the precise extraction of correlation lengths (Supplementary Note 14), the sharpening of peaks near the phase transition (Supplementary Fig. 39) suggests that the correlation lengths increase with cooling.

To estimate the lifetimes of the dynamic nanodomains, we performed quasi-elastic energy-resolved scans at the same R and M points (Supplementary Fig. 38). However, the incoherent scattering from the stochastic wobble of A-site cations overwhelms the coherent QEDS signal (Supplementary Fig. 37), preventing direct lifetime assignment. To overcome this limitation, we used MD-derived *S*(**q**, *E*) to estimate the lifetimes. Figure 3c,e shows that the MD-derived *S*(**q**, *E*) along the R–M directions in reciprocal space originates from low-energy, quasi-elastic scattering, in MAPbBr_3_ and FAPbBr_3_, respectively. By fitting vertical cross-sections of *S*(**q**, *E*) in Fig. 3c,e at every **q** to Lorentzian functions, we calculate the lifetimes of dynamic nanodomains as 1/FWHM (FWHM, full-width at half-maximum; Fig. 3d,f). Across both compositions, the lifetimes exhibit modulation along the R–M line, with MAPbBr_3_ showing sharp peaks in **q** with a maximum of 4 ps at the R point, and FAPbBr_3_ presenting broader peaks with a maximum of 1.5 ps at the M points. These are the lifetimes of pure out-of-phase (in-phase) spatially correlated octahedrally tilted nanodomains in MAPbBr_3_ (FAPbBr_3_), which we associate with QEDS peaks in **q** space at the R (M) points. These nanodomain modes exhibit the largest spatial and longest temporal correlations. Other excitations along the R–M line exhibit finite lifetimes and lack spatial correlations, representing a thermal bath of short-lived, overdamped phonons that minimally contribute to nanodomain formation. This interpretation extends previous analyses of CsPbBr_3_ and MAPbI_3_ (refs. ^20,21^), providing a more comprehensive understanding of local structural dynamics in hybrid LHPs.

### Dynamic nanodomains dictate optoelectronic response

We observed a gradual increase in the experimentally derived correlation lengths (Supplementary Fig. 39) and MD-derived lifetimes (Supplementary Fig. 27) of locally tilted nanodomains within the cubic phase of MAPbBr_3_ as the system approached the average tetragonal phase. This trend suggests that the phase transition is driven by the progressive growth and slowing down of these dynamic nanodomains. At the transition point, these nanodomains evolve into macroscopic static twins, indicating that the ferroelastic twins observed in the tetragonal phase of MAPbBr_3_ are seeded from these dynamic precursors (Supplementary Note 16). This is supported by the observation that the twinning laws in the average tetragonal phase reflect those of the dynamic nanodomains in the average cubic phase. Even in the average tetragonal *I*4/*mcm* phase, QEDS remains evident, indicating the continued presence of dynamic nanodomains (Supplementary Note 5b). These nanodomains are suppressed only when the structure transitions to an incommensurately modulated orthorhombic *Pnma* phase (Supplementary Fig. 3). By contrast, FAPbBr_3_ transitions from the cubic $Pm\bar{3}m$ to cubic $Im\bar{3}$ phase (Supplementary Note 12), accompanied by the complete disappearance of diffuse scattering. Although we cannot directly measure the nanodomain lifetimes, the striking agreement between the MD simulations and our experimental diffuse scattering data strongly supports that these nanodomains are dynamic (Supplementary Note 11 provides the full clarification). Future work using direct, time-resolved probes of structural and electronic fluctuations is expected to further confirm these findings.

**Fig. 4:**
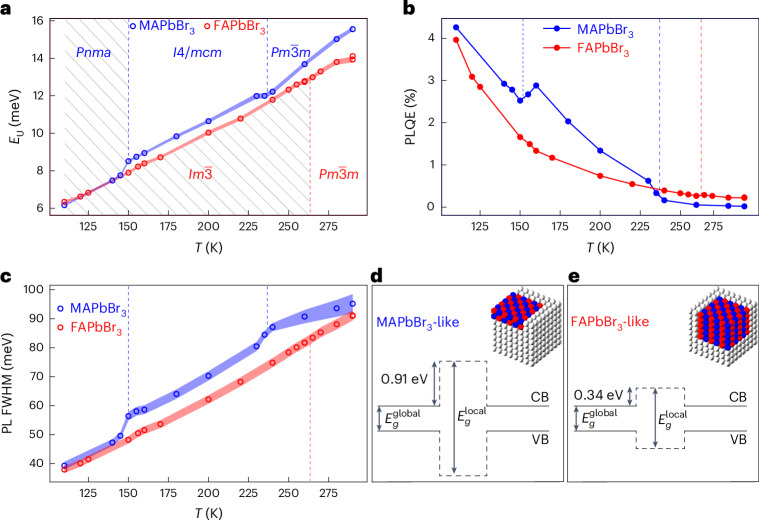
Temperature-induced structural modulation of dynamic nanodomains reveals their impact on macroscopic optoelectronic properties. **a**, Temperature-dependent Urbach energy derived by fitting the full PL spectrum to a generalized Planck’s law equation (Methods). The shaded areas around the data points represent 95% confidence intervals of the performed fits. The dashed blue lines indicate the temperature points corresponding to relevant phase transitions in MAPbBr_3_, whereas the dashed red lines represent those for FAPbBr_3_. These lines define the temperature regions for each space group of the average phase, as shown. Some of these regions include diagonal lines, indicating phases in which the local structure is absent. **b**, External PLQE as a function of temperature. **c**, FWHM of the Gaussian fits of the PL spectra as a function of temperature. Similar to that in **a**, in both **b** and **c**, the dashed blue lines indicate the temperature points corresponding to the relevant phase transitions in MAPbBr_3_, whereas the dashed red lines represent those for FAPbBr_3_. **d**,**e**, Band diagrams illustrating the local electronic structure of CsPbBr_3_ for two types of nanodomain, characteristic of MAPbBr_3_ (**d**) and FAPbBr_3_ (**e**), derived from DFT calculations^33^. CB, conduction band; VB, valence band. The insets show the structural representations of the nanodomains characteristic of MAPbBr_3_ and FAPbBr_3_, which were used to simulate the electronic structure response. Regions in which octahedra are not tilted exhibit a bandgap value of ${E}_{{\rm{g}}}^{{\rm{global}}}$, identical to that of the infinitely ordered cubic phase. Regions with locally tilted octahedra result in approximately equal conduction band offset (CBO) and valence band offset (VBO) of ~0.91 eV in MAPbBr_3_ and of ~0.34 eV in FAPbBr_3_, which results in ${E}_{{\rm{g}}}^{{\rm{local}}} > {E}_{{\rm{g}}}^{{\rm{global}}}$. The presence of these offsets computed in the static picture combined with our crystallographic local structure analysis confirms that spatial and temporal fluctuations in charge-carrier barriers within the high-symmetry average phases are a notable source of dynamic electronic disorder.

With a comprehensive understanding of the temperature-dependent structural evolution of dynamic nanodomains, we now turn to correlating this local structural information with the temperature-dependent optoelectronic response. Electronic disorder is most readily observed macroscopically by measuring sub-bandgap absorption, quantified by the Urbach energy, which comprises contributions from both static and dynamic electronic disorder^31^. We measured the temperature-dependent PL and extracted Urbach energy in MAPbBr_3_ and FAPbBr_3_ using three independent methods (Methods), all yielding consistent results (Supplementary Fig. 48). In Fig. 4a, we observe systematically higher Urbach energies in MAPbBr_3_ compared with FAPbBr_3_, as independently confirmed at room temperature by photothermal deflection spectroscopy (PDS) measurements (Supplementary Fig. 49). Following the tetragonal to orthorhombic phase transition in MAPbBr_3_, where dynamic nanodomains are suppressed, the Urbach energies of the two compounds converge. This convergence suggests that static contributions dominate at this stage, indicating comparable defect densities in both crystals. Thus, we conclude that at higher temperatures, the differences in Urbach energies are primarily attributed to dynamic electronic disorder. MAPbBr_3_ exhibits greater dynamic electronic disorder than FAPbBr_3_, which persists until the dynamic nanodomains in MAPbBr_3_ disappear near 150 K. In MAPbBr_3_, distinct changes in the slope of the Urbach energy as a function of temperature are observed near the cubic $Pm\bar{3}m$ to tetragonal *I*4/*mcm* and tetragonal *I*4/*mcm* to orthorhombic *Pnma* phase transitions. These changes correlate with abrupt changes in the local structure at these temperature points. By contrast, the cubic $Pm\bar{3}m$ to cubic $Im\bar{3}$ phase transition in FAPbBr_3_ exhibits a smooth Urbach energy curve, suggesting that the dynamic nanodomains present in the cubic phase of FAPbBr_3_ contribute minimally to dynamic electronic disorder and are largely benign.

We also extracted the FWHM of the PL peak as a function of temperature (Fig. 4c), which inherently tracks the extent of electron–phonon interactions. Notable changes are again observed near the phase transitions in MAPbBr_3_ but not in FAPbBr_3_. Furthermore, the FWHM values in MAPbBr_3_ are consistently higher, converging with FAPbBr_3_ only after the disappearance of the local structure in MAPbBr_3_. This behaviour underscores the role of dynamic electronic disorder dictated by dynamic nanodomains, where the higher disorder in MAPbBr_3_ leads to increased FWHM values. Additionally, we tracked the external PL quantum efficiency (PLQE) as a function of temperature (Fig. 4b). FAPbBr_3_ shows superior performance, with consistently higher PLQE in the high-symmetry phases. In MAPbBr_3_, a sudden increase in PLQE occurs on the cubic to tetragonal phase transition, followed by a decrease near the tetragonal to orthorhombic phase transition. This behaviour shows that changes in the local structure of MAPbBr_3_ near the phase transitions lead to anomalous PLQE trends, whereas similar structural changes in FAPbBr_3_ have minimal impact on its PLQE. It therefore follows that dynamic nanodomains in FAPbBr_3_ do not induce pronounced electronic disorder, unlike in MAPbBr_3_, where local structural changes at the phase transitions strongly modulate electronic disorder, translating into large variations in the macroscopic PLQE response.

We performed large-scale density functional theory (DFT) calculations on snapshots of 8^3^ pseudo-cubic units extracted from MD simulations using our machine learning potentials. These calculations reveal the transient localization of wavefunctions within the supercell (Supplementary Fig. 30), which has also recently been observed in FAPbBr_3_ quantum dots^32^. These observations are consistent with DFT modelling based on the experimentally determined shape and symmetry of dynamic nanodomains^33^. To isolate the effects of nanodomains, the calculations of CsPbBr_3_ with tilt domain configurations designed to emulate those in MAPbBr_3_ and FAPbBr_3_ were performed (Fig. 4d,e, respectively)^33^. The results indicate that the electronic density within the volume of these dynamic nanodomains is reduced, forming barriers for electrons and holes and leading to wavefunction localization consistent with our DFT calculations on MD snapshots. These calculations^33^ suggest that pancake-like dynamic nanodomains, as found in MAPbBr_3_, result in substantially larger barriers for both electrons and holes compared with the spherical nanodomains in FAPbBr_3_ (Fig. 4d,e). The larger dynamic barriers in MAPbBr_3_ contribute to its greater dynamic electronic disorder, leading to higher Urbach energies, lower PLQE, reduced quasi-Fermi-level splittings (Supplementary Fig. 52) and broader PL spectral response compared with FAPbBr_3_. These findings highlight how the shape of dynamic nanodomains affects the local electronic structure, directly influencing the macroscopic optoelectronic properties.

## Conclusions

By describing all the experimental diffuse scattering features, we demonstrate that high-symmetry cubic halide perovskite structures consist exclusively of dynamically and spatially correlated octahedral tilts, experimentally confirming the presence of polymorphous networks previously predicted^14^. We explicitly identify these twinned dynamic nanodomains and rule out alternative local structural correlations. The symmetry, density and shape of these dynamic nanodomains are strongly dictated by the hydrogen bonding and stochastic reorientation of the A-site cation, where the A-site cation serves as a coupling element for out-of-plane local octahedral tilts. Strong coupling and highly directional distribution of A-site orientations are required to transform default planar and highly anisotropic nanodomains into more isotropic configurations.

Through DFT calculations, we show that these dynamic nanodomains locally reduce the conduction and valence band wavefunction densities, creating transient local barriers for electrons and holes and resulting in a local increase in the bandgap. The nanodomain shape strongly influences these barrier heights, being substantially higher in anisotropic, planar nanodomains in MAPbBr_3_ compared with more isotropic, spherical nanodomains predominant in FAPbBr_3_. These dynamically evolving electronic structure landscapes fluctuate on picosecond timescales, consistent with previously proposed mechanisms^24,34^. Similar transient wavefunction localization in FAPbBr_3_ quantum dots leads to dynamic quantum confinement, facilitating efficient single photon emission^32^, underscoring how local structural dynamics both dictate bulk optoelectronic behaviour and open pathways to quantum-technology devices. Our demonstration that the cubic phases consist of ferroelastic precursor nanodomains (that is, dynamic twins) implies presence of transient strain fields^35^, suggesting why strain has emerged as such a powerful lever for controlling the properties of these unconventional semiconductors as evidenced by the recent surge in strain engineering strategies^36^. Further experiments are required to understand how these nanodomains impact heat conduction^37^, ion migration^21^ and potentially even the nucleation of performance-limiting hexagonal phases^38^.

Experimentally, we find that FAPbBr_3_ demonstrates superior optoelectronic properties relative to MAPbBr_3_, including higher PLQE, higher charge-carrier diffusion coefficients (Supplementary Fig. 47), and reduced Urbach energies and PL FWHMs. These enhancements result from the less-perturbed dynamic electronic landscape in FAPbBr_3_. Overall, our work experimentally clarifies how anharmonicity, often ambiguously described in the halide perovskite literature, concretely manifests as the correlated dynamic disorder in the form of nanodomains with well-defined local symmetry. These insights provide a foundation for the deliberate engineering of dynamic local structure to optimize perovskite-based photovoltaics, optoelectronics, X-ray detection and quantum light sources.

## Methods

### Synthesis of perovskite single crystals

MAPbBr_3_: a mixture of 1-M PbBr_2_ and 1-M MABr was dissolved in 1-ml DMF. To ensure complete dissolution, the solution was stirred vigorously at 25 °C for 6 h and then filtered with a 0.45-μm filter head before use. After adding a small MAPbBr_3_ crystal to the filtered solution, the solution was transferred to an oven. To make the crystals larger, the solution was further heated to 85 °C at a rate of 10 °C per 30 min, and the crystal reached its full size after 24 h. The obtained crystals were separated and dried to obtain MAPbBr_3_.

FAPbBr_3_: a mixture of 1-M PbBr_2_ and 1-M FABr was dissolved in 1-ml DMF/GBL (1:1). To ensure complete dissolution, the solution was stirred vigorously at 25 °C for 6 h and then filtered with a 0.45-μm filter head before use. After adding a small FAPbBr_3_ crystal to the filtered solution, the solution was transferred to an oven. To make the crystals larger, the solution was further heated to 55 °C at a rate of 10 °C per 30 min, and the crystal reached its full size after 24 h. The obtained crystals were separated and dried to obtain FAPbBr_3_.

FAPbBr_3_ grown at Colorado State University: CH(NH_2_)_2_CH_3_COO and HBr were obtained from Sigma-Aldrich. PbBr_2_ and other solvents were procured from VWR and used without further purification. In a typical preparation, approximately 0.4 g of CH(NH_2_)_2_CH_3_COO was dissolved in 8 ml of hydrobromic acid (47% v/v) at 80 °C for 15 min. Subsequently, 1.1 g of PbBr_2_ (1.25:1.0 mole ratio of CH(NH_2_)_2_CH_3_COO:PbBr_2_) was added, and the solution was stirred until all the powder had dissolved. CH(NH_2_)_2_PbBr_3_ was precipitated using ethanol as an antisolvent, and the powder was washed with ethanol. Single crystals were grown using an antisolvent method. A small vial containing 0.5 ml of a filtered 1-M solution of CH(NH_2_)_2_PbBr_3_ in a 1:1 mixture of dimethylformamide and γ-butyrolactone by volume was placed in a sealed, larger vial containing approximately 5 ml of ethanol. Crystal growth reactions were carried out over three days.

### Single-crystal X-ray diffuse scattering

We have performed various single-crystal diffraction experiments, using the following beamlines: MX1 (Australian Synchrotron), I19-1 (Diamond Light Source) and P21.1 (Deutsches Elektronen-Synchrotron); molybdenum (UNSW) and copper (Oxford) lab X-ray sources were also used. A brief summary of the methods is given below; Supplementary Note 2 provides the complete details.

Single-crystal perovskite samples were selected under a polarizing microscope (Leica M165Z) and picked up on a MicroMount (MiTeGen) consisting of a thin polymer tip with a wicking aperture. X-ray diffuse scattering measurements on MAPbBr_3_ and FAPbBr_3_ at 300 K and 200 K were carried out on the MX1 beamline at the Australian Synchrotron using X-rays of 12.9 keV with an X-ray flux of 36 × 10^11^ s^−1^ incident on an area of 120 μm × 120 μm. X-ray diffuse scattering measurements on MAPbBr_3_ and FAPbBr_3_ at 300 K and 200 K were carried out at the I19-1 beamline at the Diamond Light Source using X-rays of 18 keV with an X-ray flux of 1.542 × 10^13^ s^−1^ incident on an area of 100 μm × 100 μm. X-ray diffuse scattering measurements on MAPbBr_3_ and FAPbBr_3_ were conducted at the P21.1 beamline at the Positron-Elektron-Tandem-Ring-Anlage (PETRA III) facility, Deutsches Elektronen-Synchrotron (the data from these measurements is presented in Fig. 1a,b). An X-ray beam with an energy of 101.45 keV (*λ* = 0.1222 Å) and a size of 0.35 × 0.35 mm^2^ was used, delivering a flux of 2.5 × 10^10^ photons s^–1^. Crystals were prepared with dimensions of approximately 500 × 500 × 500 μm^3^. X-ray diffuse scattering measurements on FAPbBr_3_ at 300 K were carried out on a Rigaku Synergy S diffractometer fitted with a Dectris EIGER2 R 1M detector under copper radiation at the University of Oxford. Single-crystal diffraction measurements on MAPbBr_3_ at 200 K and MAPbI_3_ at 100 K were carried out on a Bruker D8 Quest single-crystal diffractometer with a PHOTON III detector using an IμS Incoatec Microfocus source with Mo Kα radiation (*λ* = 0.710723 Å) at UNSW.

The single crystals, mounted on the goniometer using a cryo-loop for intensity measurements, were coated with immersion-oil-type NVH and then quickly transferred to a nitrogen stream generated by an Oxford Cryostream 800 series. CrysAlisPro^39^ was used for indexing, determination and refinement of the orientation matrix. In the process of data analysis, precession images were first unwrapped using CrysAlisPro. Detailed examination of the diffraction patterns revealed that the diffuse scattering adhered to Laue symmetry. Accordingly, Laue symmetry averaging was then applied to the data, also using CrysAlisPro.

### MD simulations

To perform large-scale MD simulations of MAPbBr_3_ and FAPbBr_3_, Allegro^40,41^ machine learning force fields were trained. The training, validation and test sets were constructed based on DFT using an on-the-fly structure selection process implemented in the Vienna ab initio simulation package^42,43^, where a Gaussian-approximation-potential-style potential is fit on the fly, and training structures are picked from the MD simulations based on Bayesian error prediction^44^. Structure selection runs were performed using the NPT ensemble in MD for each of the materials using 2 × 2 × 2 pseudo-cubic supercells at six separate temperatures, namely, 100 K, 160 K, 210 K, 270 K, 350 K and 450 K. The r^2^SCAN exchange–correlation functional^45^, a plane-wave basis set with a cut-off energy of 500 eV and a 2 × 2 × 2 Γ-centred *k*-point grid were adopted. The energy threshold for electronic convergence was set to 10^−5^ eV, and a Gaussian smearing with a width of 50 meV was applied for the smearing of the electronic band occupancy. To avoid issues related to the incomplete basis set when large volume changes occur during the on-the-fly MD runs, an additional single-point DFT calculation was performed on all the structures, and these recalculated forces, energies and stresses made up the final training, validation and test sets. The full sets consisted of 2,985 and 2,640 structures for MAPbBr_3_ and FAPbBr_3_, respectively.

Separate Allegro machine learning force fields were trained for MAPbBr_3_ and FAPbBr_3_, using radial cut-offs of 6.5 Å, 2 layers and 32 tensor features with full *O*(3) symmetry and *l*_max_ = 2, a two-body latent multilayer perceptron with dimensions of [64, 128, 256, 512] and later latent multilayer perceptron with a dimension of [512], both with Sigmoid Linear Unit (SiLU) nonlinearities and a single-layer final edge-energy multilayer perceptron with a dimension of 128 and no linearity. Atomic distances were embedded using trainable Bessel functions. The models used the efficient mixed precision scheme described in ref. ^41^.

The training and validation sets contained 2,388 and 299 structures for MAPbBr_3_ and 2,112 and 264 structures for FAPbBr_3_, respectively, and were shuffled after each epoch. The training was performed using the Adam optimizer in PyTorch^46^ for 1,858 and 2,141 epochs for MAPbBr_3_ and FAPbBr_3_, respectively, using a batch size of 5 and a learning rate of 0.001. A loss function with a 1:1:1 weighing of the Allegro per atom mean squared energy, force and stress terms, respectively, was used. The trained models achieved root mean squared errors on energies, force components and stress tensor components of 0.2 meV per atom, 10 meV Å^–1^ and 0.5 kbar on hold out test sets containing 298 structures for MAPbBr_3_ and corresponding values of 0.2 meV per atom, 7 meV Å^–1^ and 0.3 kbar on hold out test sets containing 264 structures for FAPbBr_3_. Parity and error distribution plots are provided in Supplementary Note 9.

The Allegro MD simulations were performed using the Large-scale Atomic/Molecular Massively Parallel Simulator (LAMMPS) package^47^, with the pair_allegro patch^48^. Large simulation cells constructed as 20 × 20 × 20 pseudo-cubic unit cells were used for both materials and a 0.5-fs time step was used to integrate the classical equations of motion. For each material, two initial configurations were constructed, one with randomly oriented FA/MA molecules and one with perfectly aligned molecules. These initial configurations were equilibrated using fixed-shape NPT dynamics at 400 K for 200 ps, except for FAPbBr_3_ with aligned molecules where 150-ps equilibration time was used. Then, for a specific temperature of interest, a further 50 ps of equilibration was performed before running 0.5 ns of NVE dynamics, resulting in 1 ns of production NVE trajectory data for each material at each temperature. Results were cross-checked between the runs with different initial configurations and no qualitative changes were observed, indicating that the structures had been sufficiently equilibrated with respect to the molecular orientations. On the basis of these MD trajectories, real space structural dynamics analysis was performed with the PDynA package^25^.

To connect to the single-crystal X-ray diffuse scattering measurements, the dynamical structure factor *S*(**q**, *E*) was calculated from the MD trajectories using the pynamic structure factor package^49^. For each material and initial configuration, the 0.5-ns trajectories were divided into 10 blocks of 50 ps. *S*(**q**, *E*) was then averaged over these blocks and (in fractional reciprocal lattice units) over the two initial configurations. We extracted *S*(**q**, *E*) for 0 ≤ H, K, L ≤ 5 and expanded these values to the whole plane by mirroring in the coordinate axes. Also, *q*-dependent atomic form factors, approximated as a sum of Gaussians, were used as described elsewhere^21^ and implemented in the pynamic structure factor package^49^.

### Inelastic neutron scattering

Constant-energy, variable-**q** (momentum) scans were performed using the thermal triple-axis spectrometer Taipan at the Australian Nuclear Science and Technology Organisation (ANSTO). Taipan was aligned with o–40′–40′–o collimation, in a configuration in which the incident neutron energy was varied with a fixed final scattered neutron energy of 14.87 meV. A graphite filter was used on the scattered side to remove higher-order scattering. The two large single crystals (about 1 cm^3^ in volume), MAPbBr_3_ and FAPbBr_3_ were aligned in the [H, H, L] and [H, K, 0] planes, respectively.

The cold triple-axis spectrometer SIKA at the ANSTO was aligned with o–60′–60′–60′ collimation using a large double-focusing pyrolytic graphite monochromator and analyser, which were oriented with a fixed final energy of 5 meV. This allowed an energy resolution of approximately 0.195 meV FWHM. The cooled Be filter was used to remove unwanted higher-order reflections. Samples were wrapped in a thin Teflon tape to prevent perovskite crystal surface from reacting with Al, and then mounted on an Al plate before being inserted into the cryostat. Counting times were approximately 5 min per point at the incident flux density of 1.5 × 10^6^ neutrons cm^−^^2^ s^−^^1^.

### Hyperspectral PL microscopy

Wide-field, hyperspectral PL measurements were conducted using a Photon etc. IMA system. A ZEISS Plan-Neofluar objective lens with a numerical aperture (NA) of 0.75 and ×63 magnification was used for all the measurements. To reduce degradation caused by oxygen and humidity, samples were stored in a nitrogen-filled glovebox until just before measurement. For the temperature-dependent experiments, the samples were fixed with silver paste to the cold finger of an Oxford HiRes Microstat cooled with liquid helium. The sample was held at the set temperature for at least 15 min before every measurement. The samples were cooled down/heated up with a maximum rate of 1° min^–1^ to avoid any unexpected thermal stress effects. A reference sample was used to calibrate the apparatus and determine the post-processing parameters necessary for correcting the image distortion caused by the optical elements in the detection path. Chromatic aberrations were mitigated by automatically changing the *z* position (focus) of the sample for every collected central wavelength based on a previously performed calibration measurement.

A 400-nm picosecond pulsed laser operating at a repetition rate of 100 kHz and a fluence of 0.68 μJ cm^−2^ with a top-hat profile (size, 150 μm × 150 μm) was used as the excitation source for temperature-dependent PL measurements. This repetition rate was selected as the highest value at which no laser-induced decrease in PL intensity was observed in a vacuum, a common indicator of laser-induced damage. To ensure clean and consistent surface responses, single crystals were cleaved before each measurement. Using a microscope, PL was collected from a field of view of 85 μm × 85 μm, which was confirmed to be free of observable surface heterogeneities. Although hyperspectral microscopy enables the collection of PL spectra at every pixel, to improve the signal-to-noise ratio, particularly crucial at higher temperatures at which PL is weaker, the PL response was integrated across the entire field of view. This overall approach ensured that the measured PL represented the intrinsic properties of the material, minimizing contributions from surface morphology and contamination. This methodology is essential for accurately probing the material’s true optoelectronic response, as PL near rough surfaces or cracks can be substantially altered. Unlike macroscopic PL, which is usually collected from a larger area and lacks precise surface quality control, this approach provides a more reliable characterization of the intrinsic PL properties of single crystals.

The excitation laser was separated from the PL signal using a high-quality 405-nm Semrock dichroic mirror, effectively filtering the excitation light. The emitted light from the sample was then directed onto a volume Bragg grating, which dispersed it spectrally before being detected by a Hamamatsu ORCA-Flash4.0 V3 scientific complementary metal-oxide-semiconductor (sCMOS) camera. This camera features a 2,048 × 2,048 pixel^2^ array, with each individual pixel measuring 6.5 μm × 6.5 μm.

For each objective lens, a two-step calibration process was conducted to determine the absolute number of photons at each point. Initially, a calibrated white light lamp was directed through the objective lens into an integrating sphere. By comparing the measured lamp spectrum at each point with its known spectrum, the system’s relative sensitivity was established both spectrally and spatially. In the second step, a 657-nm laser with a known optical power was fibre-coupled and imaged through the microscope. A hyperspectral measurement of the fibre output allowed for the conversion between detected counts and photons at this specific wavelength. By combining this absolute calibration with the relative calibration obtained from the white light lamp and integrating sphere, we accurately quantified the number of photons emitted across the spectrum at each point on the sample. More precisely, we determined the number of photons eV^–1^ s^–1^ cm^–2^ sr^–1^ detected per pixel (sr, steradians).

The details about PL FWHM, Urbach energy (*E*_U_), quasi-Fermi level splitting (Δ*μ*) (Supplementary Fig. 52) and the external PLQE measurements are provided in Supplementary Note 17.

### Confocal PL diffusion measurements

Time-resolved PL diffusion measurements were performed using a confocal microscope setup (PicoQuant, MicroTime 200). Freshly cleaved crystals were excited by a 405-nm pulsed laser (PDL 828, PicoQuant, 10 MHz, 19–22 μJ per pulse, 11.6–13.4 μJ cm^–2^, ~100-ps pulse width) focused through an air objective (×100, 0.9 NA). The emission signal was collected by the same objective and separated from the excitation light using a 405-nm longpass dichroic mirror (Z405RDC, Chroma).

To obtain the diffusion maps, the emission signals were scanned pixel by pixel using a Galvano scanner as the excitation source remained at a fixed position. Time-resolved PL signals subsequently passed through a 450-nm longpass filter and a 50-μm pinhole before being focused onto a PMA Hybrid 42 detector for time-correlated single-photon counting (time resolution, 100 ps).

The diffusion coefficients were extracted by tracking the expansion of the spatial PL distribution as a function of time, where we fit a Gaussian function to the PL profile at different time points. Since the observed diffusion behaviour exhibits a linear relationship between the squared standard deviation (*σ*^2^) and time (*t*), we determined the diffusion coefficient (*D*) according to the linear mean squared displacement model, where

$${\sigma }^{2}(t)={\sigma }^{2}(0)+2Dt\,.$$

On the basis of this model, we found the diffusion coefficients to be 0.40 cm^2^ s^–1^ and 0.27 cm^2^ s^–1^ for FAPbBr_3_ and MAPbBr_3_, respectively. The raw data and the corresponding fits are provided in Supplementary Fig. 47.

### PDS

PDS measurements were conducted at room temperature using single crystal samples mounted inside a quartz cuvette filled with a thermo-optic liquid (3M Fluorinert FC-72). The excitation source was a halogen lamp paired with a 250-mm-focal-length grating monochromator, delivering tunable light-beam wavelengths for spectral scans. The light beam was modulated at 10 Hz using a mechanical chopper. The samples were excited using this monochromatic pump beam, generating heat through non-radiative recombination that induced an alternating temperature gradient at the sample surface. The PDS experiments were performed in the transverse configuration, with a continuous-wave probe laser beam (670 nm) passing parallel to the excitation area near the surface. The absorption-dependent deflection of this probe was detected using a quadrant silicon photodiode and measured synchronously using a lock-in amplifier (Stanford Research Systems SR830). This method provided a signal proportional to the absorbance, offering a high dynamic range and minimizing the scattering effects typically encountered in ultraviolet–visible spectroscopy. The *E*_U_ values were extracted from the absorption edge, at energies at which the absorption (*A*) is exponentially related to the photon energy according to

1 $$A(E)={A}_{0}\exp \left(\frac{E-{E}_{{\rm{g}}}}{{E}_{{\rm{U}}}}\right)\,,$$

where *A*_0_ is the optical absorption coefficient and *E*_g_ is the bandgap energy. For each crystal, the measurements were performed at three different spots to ensure accuracy.

### Low-temperature optical microscopy

Samples were measured in an Oxford HiRes Microstat cooled using liquid helium and kept in a vacuum during the measurement. The emergence of ferroelastic nanodomains was observed using the Photon Etc. IMA microscopy system. Here ×20 (Nikon TU Plan Fluor, 0.45 NA) and ×100 long-working-distance objective (Nikon TU Plan Fluor, 0.8 NA) with appropriate chromatic aberration corrections were used for all measurements. Single crystals were cleaved immediately before measurements to obtain a fresh and uniform surface and then mounted in an open-cycle liquid-helium cryostat. The samples were cooled down/heated up with a maximum rate of 1° min^–1^ to avoid any unexpected thermal stress effects. The images were taken using a high-sensitivity camera (Hamamatsu ORCA-Flash4.0 V3 sCMOS camera) with 2,048 × 2,048 pixels^2^ (each pixel, 6.5 × 6.5 μm^2^) that was thermoelectrically cooled to –10 °C.

### Differential scanning calorimetry

Differential scanning calorimetry measurements were performed on a Netzsch DSC Proteus 204 F1 device in an Al crucible under a N_2_ atmosphere. The samples were cooled and heated in the temperature range of 115–330 K with a scan rate of 10 K min^–1^. The masses of the MAPbBr_3_ and FAPbBr_3_ samples were 19.9 mg and 20.3 mg, respectively.

## Online content

Any methods, additional references, Nature Portfolio reporting summaries, source data, extended data, supplementary information, acknowledgements, peer review information; details of author contributions and competing interests; and statements of data and code availability are available at 10.1038/s41565-025-01917-0.

## Supplementary information

Supplementary information Supplementary Notes 1–17, Figs. 1–52 and Tables 1–9.

## Data Availability

The data that support the findings of this study are available to download at the University of Cambridge Apollo Repository at 10.17863/CAM.116346.

## Code availability

The MATLAB code used for the phenomenological model of the local octahedral tilts is publicly available at https://github.com/dubajicmilos/LocalOctaTilt. The machine learning interatomic potential model, Allegro, used for training and MD simulations, is available at https://github.com/mir-group/allegro and https://github.com/mir-group/nequip. MD simulations were performed using LAMMPS with the Allegro potential, accessible at https://github.com/lammps/lammps and https://github.com/mir-group/pair_allegro. The dynamic structure factor was computed from MD trajectories using the pynamic structure factor, available at https://github.com/tyst3273/pynamic-structure-factor. Real space analysis of MD trajectories was performed using PDynA, which can be accessed at https://github.com/WMD-group/PDynA. DFT calculations for the training data were performed using the Vienna ab initio simulation package (https://www.vasp.at), which is a licensed software package.
