# Supplementary material for: Dynamic nanodomains dictate macroscopic properties in lead halide perovskites
Source: Nat Nanotechnol. 2025 Jun 2;20(6):755–63. doi: 10.1038/s41565-025-01917-0 (PMC12181079; doi:10.1038/s41565-025-01917-0)
Supplement: Supplementary file 1 — Supplementary Notes 1–17, Figs. 1–52 and Tables 1–9. [file 41565_2025_1917_MOESM1_ESM.pdf]

nature nanotechnology

Supplementary information https://doi.org/10.1038/s41565-025-01917-0

Dynamic nanodomains dictate macroscopic properties in lead halide perovskites

In the format provided by the authors and unedited

TABLE OF CONTENTS

1. Phase transitions, average and local structure in halide perovskites 2

2. Single crystal X-ray diffuse scattering 3

3. Twinning observation using single crystal XRD 5

4. Modified Glazer notation for local octahedral tilting correlations 7

5. The phenomenological model of local octahedral tilting 7
   a. Implementation of the phenomenological model for quasi-elastic diffuse scattering simulations 7
   b. Local structure in the average tetragonal phase. 11
   c. Comparison between experimental diffuse scattering patterns and those generated by the phenomenological model. 12

6. Molecular dynamic simulations 19
   a. Comparison of experimental and MD computed diffuse scattering patterns 19
   b. The origin of quasi-elastic diffuse scattering at X points 20
   c. Effect of different A-sites on dynamic structure 25

7. Procedure to separate TDS and QEDS in the experimental data and the physical interpretation of their relative intensities 26

8. The quantitative analysis of spatial correlations from MD data 29

9. Verification of the Allegro Machine Learned Force Fields 35

10. Analysis of electronic structure from MD data 36

11. Arguments in favour of the dynamic nature of nanodomains 36

12. The assignment of lower temperature phase of $FAPbBr_3$ 38

13. The influence of beam damage on local structure 40

14. Inelastic neutron scattering 42

15. Direct observation of presence of ferroelastic twin domains in $MAPbBr_3$ and their absence in $FAPbBr_3$ single crystals 47

16. Interplay between local octahedral tilting and ferroelastic phase transtion 48

17. Optoelectronic characterization of single crystals 49

References 56

1. PHASE TRANSITIONS, AVERAGE AND LOCAL STRUCTURE IN HALIDE PEROVSKITES

We used differential scanning calorimetry (DSC) to determine phase transition temperatures in our materials. DSC is a thermal analysis technique employed to determine the heat flow into or out of a sample by simultaneously applying heat to both the sample and a reference material whilst recording their respective temperatures. The internal energy changes in the sample can subsequently be inferred from the measured temperature difference [1]. DSC is a highly effective tool for identifying the temperature points at which structural phase transitions occur [2–4]. As the lattice adopts a new configuration during the phase transition, heat is exchanged with the environment, resulting in a peak on the DSC curve. DSC measurements were carried out on $MAPbBr_3$ and $FAPbBr_3$ single crystals. The corresponding results are presented in Supplementary Figure 1 **a** and **b**, respectively. Temperature points indicative of potential phase transition events are depicted above the corresponding DSC curve peaks in Supplementary Figure 1.

DSC peaks in the heating cycle at $T = 235$ K, $T = 155$ K, and $T = 150$ K can be assigned to cubic-tetragonal (tetragonal $I4/mcm$ shown in Supplementary Figure 2 **b**), tetragonal-incommensurate and incommensurate-orthorhombic [5–7] phase transitions in $MAPbBr_3$, based on prior DSC investigations [8–11]. Numerous efforts have been made to identify transition temperatures and space groups for each phase of $FAPbBr_3$ [9, 12–16]. While a consensus exists in the literature on space groups, temperature transition points remain ambiguous. $FAPbBr_3$ undergoes a series of structural phase transitions with decreasing temperature. Consequently, the $T = 264$ K transition (as seen in Supplementary Figure 1 **b**) has previously been assigned to the cubic-tetragonal transition (tetragonal $P4/mbm$ shown in Supplementary Figure 2 **b**), whereas it remains unclear which individual transitions correspond to the other observed DSC peaks (the orthorhombic $Pnma$ phase depicted in Supplementary Figure 2 **d** was most commonly identified at low temperatures). The measured DSC curves show a good agreement with previously reported DSC measurements in $MAPbBr_3$ [8–10] and $FAPbBr_3$ [9, 13, 17].

The data presented in Supplementary Figure 1 confirms that between $T = 300$ K and $T = 200$ K in these materials, only one phase transition occurs. As we refined the space groups at those two temperatures the DSC data supports our claims from the main text that $MAPbBr_3$ transitions from cubic $Pm\bar{3}m$ to twinned $I4/mcm$ at 235 K while $FAPbBr_3$ transitions from cubic $Pm\bar{3}m$ to cubic $Im\bar{3}$ at 264 K.

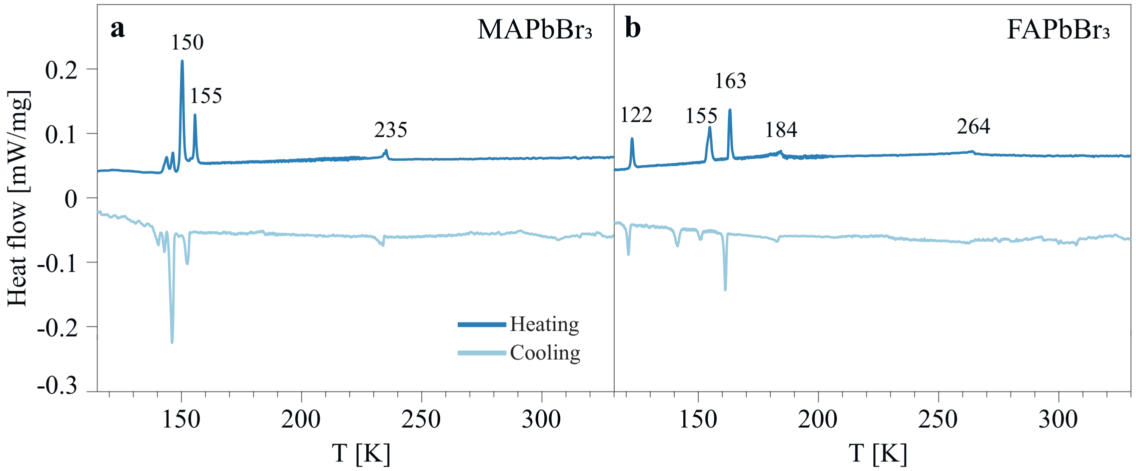

Supplementary Figure 1: **Differential scanning calorimetry (DSC) measurements.** DSC heating and cooling cycles of (**a**) $MAPbBr_3$ (**b**) and $FAPbBr_3$.

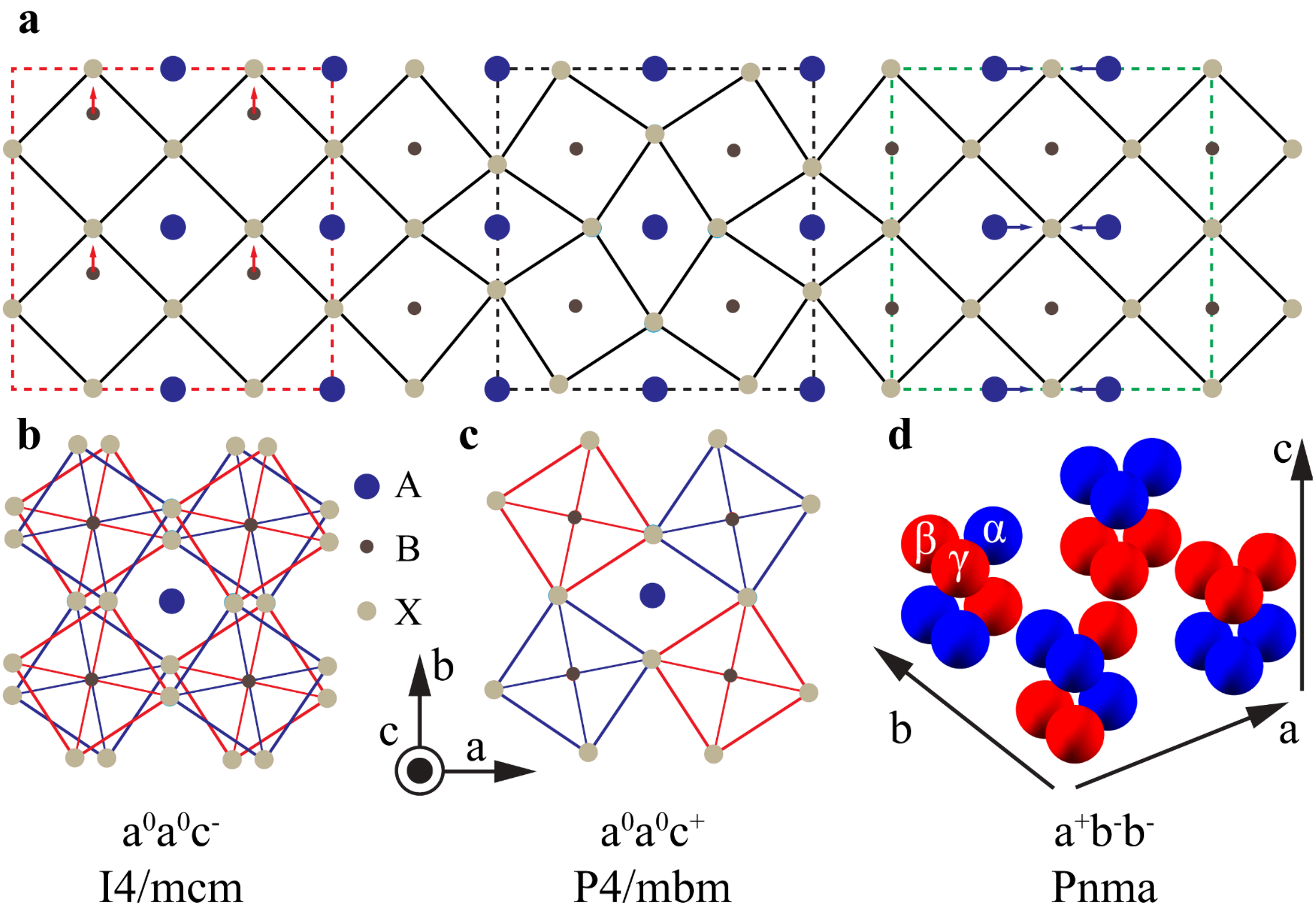

Supplementary Figure 2: **Graphical illustration of local and average structures in perovskite-type** ABX_3 **lattice.** (**a**) Depiction of various short-range atomic correlations. From left to right: B-site off-centering within the red rectangle, octahedral tilt correlations within the black rectangle, and A-site correlations within the green rectangle. Octahedral tilt patterns across various space groups with corresponding Glazer notation given in brackets (**b**) tetragonal $I4/mcm$ ($a^0a^0c^-$), (**c**) tetragonal $P4/mbm$ ($a^0a^0c^+$), (**d**) orthorhombic $Pnma$ ($a^+b^-b^-$). In (**b**) and (**c**) octahedron edges are depicted in blue and green and indicate positive and negative tilts along the c direction. In (**d**), each octahedron is depicted with three spheres in a triangular arrangement, with colours representing the tilt angle's direction: blue for positive and red for negative, along the a (α), b (β), and c (γ) axes.

2. SINGLE CRYSTAL X-RAY DIFFUSE SCATTERING

Single crystal perovskite samples were selected under a polarizing microscope (Leica M165Z) and picked up on a MicroMount (MiTeGen, USA) consisting of a thin polymer tip with a wicking aperture. X-ray diffuse scattering measurements on $MAPbBr_3$ and $FAPbBr_3$ at 300 K and 200 K were carried out on MX1 beamline at Australian Synchrotron using X-rays of 12.9 keV with X-ray flux of $36 \times 10^{11}\,s^{-1}$ incident on an area of 120 µm x 120 µm. The detector used was Dectris EIGER2 Si 9M (3108 × 3262 pixels, 75 μm × 75 μm pixel size) at a distance of 105 mm. The crystal dimensions measured for $MAPbBr_3$ were 200 µm x 70 µm x 50 µm, while those for $FAPbBr_3$ were 100 µm x 70 µm x 70 µm. The single crystals, mounted on the goniometer using a cryo loop for intensity measurements, were coated with immersion oil type NVH and then quickly transferred to the nitrogen stream generated by an Oxford Cryostream 800 series. CrysAlisPro [18] was used for indexing, determination and refinement of the orientation matrix. In the process of data analysis, precession images were first unwrapped using CrysAlisPro. Detailed examination of the diffraction patterns revealed that the diffuse scattering adhered to Laue symmetry. Accordingly, Laue symmetry averaging was then applied to the data, also using CrysAlisPro. The average structure of $FAPbBr_3$ at 300 K and 200 K was solved by program SHELXT (with intrinsic phasing) [19] and the full-matrix least-squares refinements were carried out using SHELXL-2014 [20] through the Olex2 [21] software suite.

X-ray diffuse scattering measurements on $MAPbBr_3$ and $FAPbBr_3$ at 300 K and 200 K were carried out at I19-1 beamline at Diamond Light Source using X-rays of 18 keV with X-ray flux of $1.542 \times 10^{13}\,s^{-1}$ incident on an area of 100 µm x 100 µm. Each diffraction image was collected with a Pilatus2M detector (1679 × 1475 pixels, 172 × 172 μm^2 pixel size). The single crystals, mounted on the goniometer using a cryo loop for intensity measurements, were coated with immersion oil type NVH and then quickly transferred to the nitrogen stream generated by an Oxford Cryostream 800 series. In the process of data analysis, precession images were unwrapped using CrysAlisPro. To enhance the signal-to-noise ratio while minimizing the X-ray dose received by the samples, we repeated the measurement between 10 to 25 times. Subsequently, we summed these frames in post-processing. This approach allowed us to extend the dynamic range of the measurement, enabling us to capture both Bragg peaks without saturating the detector and to detect weak diffuse scattering.

X-ray diffuse scattering measurements on $FAPbBr_3$ at 300 K were carried out on a Rigaku Synergy S diffractometer fitted with a Dectris EIGER2 R 1M detector at the University of Oxford. All data sets were collected under copper radiation (λ= 1.5406 Å). Crystals were mounted on a 0.2 mm diameter MiTeGen loop using Paratone-N oil as a cryoprotectant. An exposure time of 90 s ($\theta = 40°$) or 120 s ($\theta = 80°$) was needed to detect diffuse features at room temperature (300 K). Each measurement involved a full ϕ-scan carried out in a single run. CrysAlisPro was used for indexing, determination and refinement of the orientation matrix. For the diffuse scattering analysis the scattering data were reconstructed on a three-dimensional grid defined by -10 $\leq h, k, l \leq$ +20 with voxel sizes of $\Delta h = \Delta k = \Delta l = 0.05$ r.l.u., resulting in an array of 401 × 401 × 401 voxels. For this purpose, the crystal orientation as refined with CrysAlisPro was converted using a customized code to serve as input for the Meerkat program [22]. After careful inspection of the diffraction data it was observed that the diffuse scattering also follows Laue symmetry. The data were subsequently averaged for Laue symmetry using Meerkat.

X-ray diffuse scattering measurements on $MAPbBr_3$ and $FAPbBr_3$ were conducted at the P21.1 beamline at the Positron-Elektron-Tandem-Ring-Anlage (PETRA III) facility, Deutsches Elektronen-Synchrotron (DESY). An X-ray beam with an energy of 101.45 keV (λ = 0.1222 Å) and a size of 0.35 × 0.35 mm^2 was used, delivering a flux of 2.5×10^{10} photons/s. Crystals were prepared with dimensions of approximately 500 × 500 × 500 μm^3. Each crystal was mounted on the tip of an amorphous cactus needle using epoxy and secured to a goniometer head. The crystal was carefully aligned to ensure it remained at the center of rotation and within the X-ray beam during measurements. For temperature dependent measurements N-HeliX Open Flow Helium Gas Flow System from Oxford Cryosystems was used. For the calibration of beam parameters Standard Reference Material 1990, Al_2O_3 Single Crystal Diffractometer Alignment Standard from NIST was used. Diffraction images were collected using a Pilatus2M detector (1679 × 1475 pixels, 172 × 172 μm^2 pixel size) positioned at distances of 400 mm and 700 mm. A single 3D dataset was obtained by rotating the crystal through a full 360° along a single axis, with the detector continuously counting and reading out at regular intervals. Exposure time was 0.2 s per frame, producing either 1850 or 3700 total images per rotation (corresponding to 0.2° and 0.1° per image, respectively). For each sample at every temperature, three different 3D datasets were collected at varying detector positions, all at the same distance, to ensure coverage of the gaps between the detector chips. Data processing was carried out using a *Matlab* repository [23], which generated dynamic detector masks, determined crystal orientation (UB) matrices, and transformed raw detector images into reciprocal space. For diffuse scattering analysis, data were reconstructed onto a three-dimensional grid defined by $-7 \leq h, k, l \leq +7$, with voxel sizes of $\Delta h = \Delta k = 0.0274$ r.l.u. and $\Delta l = 0.0549$ r.l.u., resulting in an array of 512 × 512 × 256 voxels. After careful inspection of the diffraction data it was observed that the diffuse scattering also follows Laue symmetry. The data were subsequently averaged for Laue symmetry using a *Matlab* repository [23].

Single crystal diffraction measurements on $MAPbBr_3$ at 200 K and $MAPbI_3$ at 100 K were carried out on a Bruker D8 Quest Single Crystal diffractometer with PHOTON III detector at different temperatures using $I\mu S$ Incoatec Microfocus source with $Mo - K\alpha$ radiation ($\lambda = 0.710\,723$ Å). The single crystals, mounted on the goniometer using a cryo loop for intensity measurements, were coated with immersion oil type NVH and then quickly transferred to the nitrogen stream generated by an Oxford Cryostream 700 series. Symmetry-related absorption corrections using the program SADABS were applied and the data were corrected for Lorentz and polarisation effects using Bruker APEX3 software. Precession images were generated in CrysAlisPro software.

In this document, we present single-crystal X-ray diffuse-scattering data as two-dimensional slices through three-dimensional reciprocal space. To simplify notation, a cut taken at e.g. $L = 1.5$ (the $(H, K, L = 1.5)$ plane) is referred to as "HK1.5."

3. TWINNING OBSERVATION USING SINGLE CRYSTAL XRD

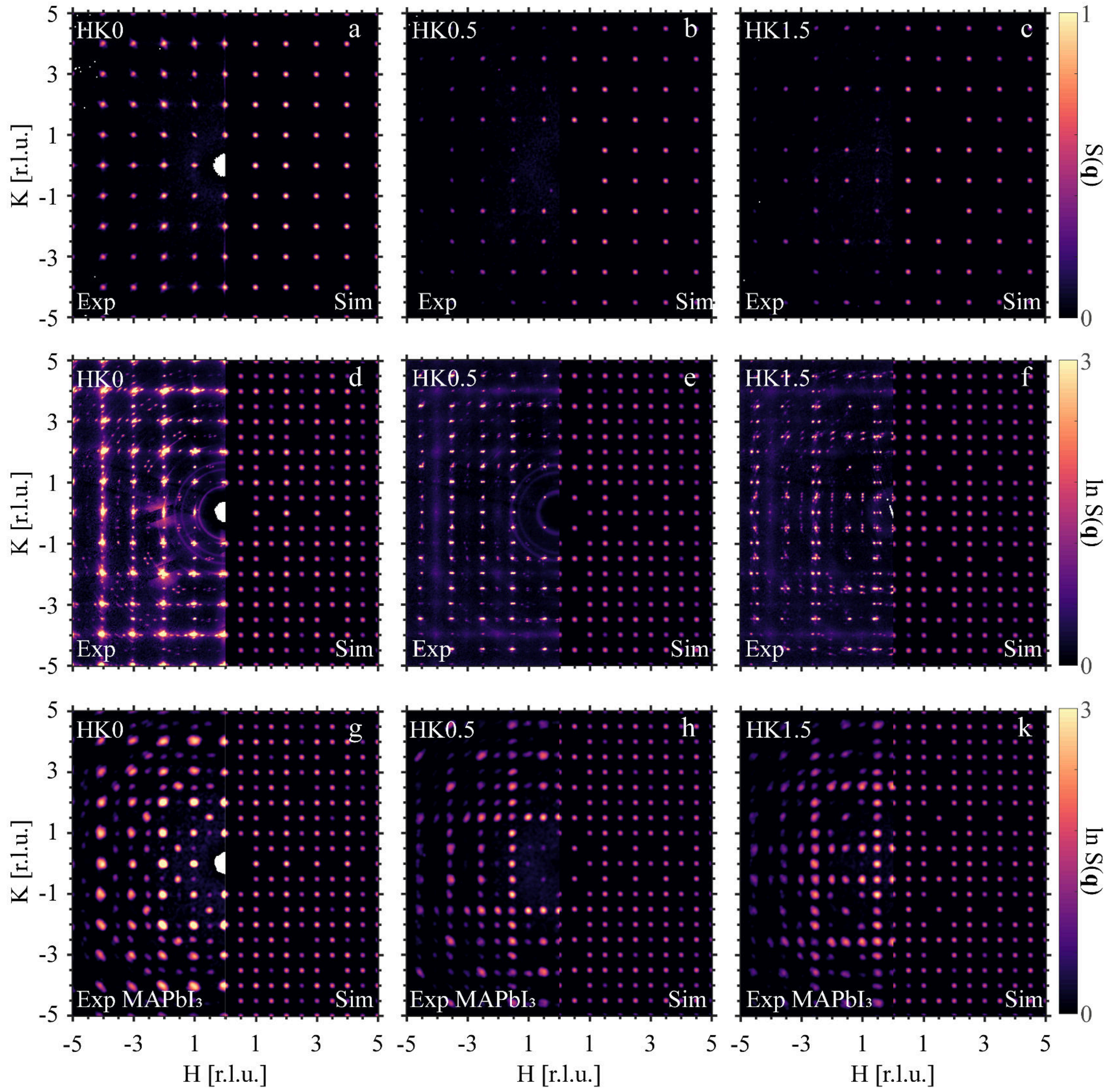

Supplementary Figure 3: **Non-Merohedral twinning and incommensurate modulation observed in reciprocal space.** The left panels in (**a**), (**b**), and (**c**) display the experimental scattering function $S(\mathbf{q})$ at $T = 200\,\mathrm{K}$, while the corresponding right panels illustrate the simulated twinned average $I4/mcm$ phase scattering function in the HK0, HK0.5, and $HK1.5$ planes of $MAPbBr_3$, respectively. Similarly, the left panels in (**d**), (**e**), and (**f**) showcase the experimental scattering functions at $T = 100\,\mathrm{K}$, with the corresponding right panels depicting the simulated twinned average $Pnma$ phase scattering function in the HK0, HK0.5, and $HK1.5$ planes of $MAPbBr_3$, respectively. The additional satellite peaks are present around superstructure $Pnma$ peaks that indicate the incommensurate modulation of $Pnma$ phase present at this temperature. The left panels in (**g**), (**h**), and (**k**) showcase the experimental scattering functions of $MAPbI_3$ at $T = 100\,\mathrm{K}$, with the corresponding right panels depicting the simulated twinned average $Pnma$ phase scattering function in the HK0, HK0.5, and $HK1.5$ planes, respectively. The absence of satellite peaks is noticeable indicating the absence of incommensurate modulation. A noticeable discrepancy between the simulated and experimental Bragg peak intensities is apparent. This arises due to the assumption in our simulation that all twin components have an equal likelihood of occurring within the crystal.

We observe non-merohedral twinning, a type of twinning where the twin domains are usually related to each other by a symmetry operation of the higher symmetry phase that is not a symmetry operation of the lower symmetry phase. This occurs during the ferroelastic cubic-to-tetragonal phase transition in $MAPbBr_3$ while this twinning is not present during the phase transition in $FAPbBr_3$. We have managed to capture this transition in $MAPbBr_3$ in both reciprocal space (Supplementary Figure 3 **a**, **b** and **c**) and in real space (Supplementary Figure 43 **a** and **b**). Based on X-ray scattering factors in reciprocal space we identified the symmetry relationships that define this non-merohedral twinning (given in Supplementary Table I and depicted in Supplementary Figure 4 **a**). It can be observed that the peak intensities of the experimental and simulated data do not exactly match. This is a result of our assumption that all twin components occur with the same probability, which is in general not the case. However, our simulated pattern does match the experimental X-ray Bragg pattern in all the planes presented in Supplementary Figure 3. This confirms that our twinning laws correctly capture the non-merohedral twinning which occurs during cubic-tetragonal and tetragonal-orthorhombic phase transitions in $MAPbBr_3$. We consistently observe additional satellite peaks below 150 K near the superstructure peaks corresponding to the *Pnma* space group (Supplementary Figure 3 **d-f**), indicating incommensurate modulation of the *Pnma* phase. This distinct incommensurate structure has been previously observed [5] and differs from the incommensurate modulation of the *Imma* phase, which occurs just above the orthorhombic phase transition [6, 7]. We note that, for example, this incommensurate modulation is not present at 100 K in $MAPbI_3$ and that the structure can be described as twinned *Pnma* (Supplementary Figure 3 **g-k**). Twinning laws are generally given through rotation operators that act on a reference twin component (in our case D_1) and we visualise them in Supplementary Figure 4.

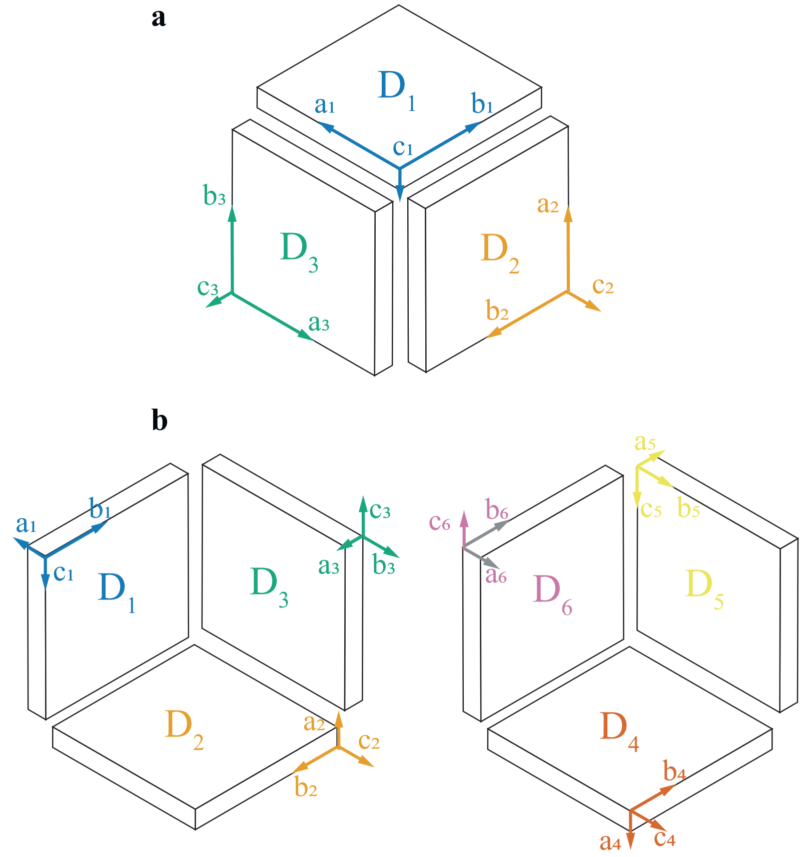

Supplementary Figure 4: **Illustration of twinning transformations.** (**a**) The diagram illustrates the relative orientations of the coordinate systems for three twin components that emerge during cubic to tetragonal ferroelastic phase transition in $MAPbBr_3$. The transformation operators can be found in Supplementary Table I. The same twin laws apply to the corresponding local structure, i.e., local tetragonal planar nanodomains in average cubic phase. (**b**) The diagram shows the relative orientations of the coordinate systems for six twin components that occur during the transition from a tetragonal to an orthorhombic phase in $MAPbBr_3$.

4. MODIFIED GLAZER NOTATION FOR LOCAL OCTAHEDRAL TILTING CORRELATIONS

The Glazer notation offers a systematic approach to describe octahedral tilting patterns in perovskite structures [24]. In the Glazer notation, the sequence of symbols directly corresponds to the pseudocubic crystallographic axes: the first symbol for tilts along a [100], the second along b [010], and the third along c [001]. The set of three letters (e.g., abc) is also used to symbolise the magnitudes of tilt about these axes, with identical letters indicating equal tilt amplitudes. Each symbol is accompanied by a superscript denoting the tilt's ordering across adjacent octahedral layers: '0' for no tilt, '+' in-phase ordering (where adjacent layers tilt in the same direction), and '-' anti-phase ordering (where adjacent layers tilt in opposite directions). For instance, $a^0a^0c^-$ indicates no tilt along the a and b axes and an infinite anti-phase ordering of c-axis tilts along the c axis. Due to the rigid octahedra rules, if one octahedron is arbitrarily rotated along e.g. the c axis, the adjacent octahedra always anti-phase order along a and b while the ordering along the c axis is not constrained [24].

An arbitrary rotation of one octahedron along the c axis induces tilting of neighbouring octahedra, resulting in the formation of a local structure. Consequently, there will be no infinite ordering along the a and b axes. Instead, a long-range ordering, denoted by the subscript L in the modified Glazer notation, will be present. In this example, the tilt perturbation along c will be significantly weaker due to the rigidity of the octahedra, resulting in short-range ordering along c, which we denote by the subscript S. This phenomenon can be described using the notation $a^0a^0c^{---}_{LLS}$ for short-range anti-phase ordering of c-axis tilts along the c axis. The local structure resembles $I4/mcm$ symmetry, observable in the average cubic phase of $MAPbBr_3$. The three subscripts under c indicate whether the range of ordering of c-axis tilts along the [100], [010] and [001] pseudocubic axes is long ('L') or short ('S'). Similarly, $a^0a^0c^{--+}_{LLS}$ corresponds to short-range in-phase ordering of c-axis tilts along the c axis, with a local structure akin to $P4/mbm$ symmetry, as found in the average cubic phase of $FAPbBr_3$.

Given that long-range ordered octahedra are always in-plane and always exhibit anti-phase ordering, we can exclude L subscripts and just denote the sign of short range (S) ordering. For infinite correlations we do not use L or S but just denote the ordering sign. Thus, $a^0a^0c^{---}_{LLS}$ can be reduced to $a^0a^0c^-_S$ and $a^0a^0c^{--+}_{LLS}$ to $a^0a^0c^+_S$.

5. THE PHENOMENOLOGICAL MODEL OF LOCAL OCTAHEDRAL TILTING

a. Implementation of the phenomenological model for quasi-elastic diffuse scattering simulations

We initially performed temperature-dependent single crystal diffraction on halide perovskites to capture all global crystallographic phases. Standard single crystal XRD structural refinements were subsequently carried out, and from the resulting CIF files, structure factors and Miller indices were extracted. The experimentally observed X-ray scattering function, $S(\mathbf{q})$, contains both Bragg peaks, arising from infinite correlations (global order), and diffuse scattering, originating from local spatial correlations. Our analysis revealed that the symmetry of the local structure within a global crystallographic phase generally corresponds to that of the next lower-temperature global crystallographic phase. Therefore, $S(\mathbf{q})$ can be expressed as the sum of the contributions from the high-symmetry global structure (infinitely ordered) and the lower-symmetry local structure (short-range ordered).

We will describe a procedure to generate 3D volumetric reciprocal space intensities for the global structure $S^{glob}(q^*)$, without any short-range order, using corresponding CIF file.

1. We first apply the transformation matrix to convert the structure factors F_{hkl} and corresponding Miller indices (h, k, l) from the CIF file, originally defined in the native space group, to their equivalents in pseudocubic notation.

2. To simplify the calculations, we assume that each reflection generates a 3D Gaussian profile in reciprocal space, with an instrument resolution-limited variance σ^2 (specific values for each instrument are provided in Supplementary Table II). This allows us to compute the 3D volumetric intensity distribution $I^{\mathrm{glob}}_{hkl}(\mathbf{q}^*)$ for each reflection:

$$I^{glob}_{hkl}(q^*_H, q^*_K, q^*_L) = |F^{glob}_{hkl}|^2 \exp\left(-\frac{(q^*_H - h)^2 + (q^*_K - k)^2 + (q^*_L - l)^2}{2\sigma^2}\right), \qquad \text{(S1)}$$

where $\mathbf{q}^* = (q^*_H, q^*_K, q^*_L)$ represents the components of the scattering vector $\mathbf{q}$ reduced to reciprocal lattice units, defined by $\mathbf{q}^* = \frac{a\mathbf{q}}{2\pi}$, where a is the pseudocubic lattice constant.

3. The intensity distributions of all reflections $I_{hkl}^{glob}(\mathbf{q}^*)$ are summed to obtain the volumetric intensity distribution of the global structure $S^{glob}(\mathbf{q}^*)$:

$$S^{glob}(\mathbf{q}*) = \sum_{h,k,l} I_{hkl}^{glob}(\mathbf{q}*) \,. \tag{S2}$$

To incorporate the local structure contribution to the volumetric intensity distribution, we assume that diffuse peak intensities follow a Gaussian distribution. The variance of the 3D elliptical Gaussian function is $\Delta\sigma_i$, which is inversely proportional to the correlation length ξ_i along a particular direction $i = H, K, L$ in reciprocal space. Thus, Eq. (S1) is modified into:

$$I_{hkl}^{\text{loc}}(\mathbf{q}*) = |F_{hkl}^{\text{loc}}|^2 \exp\left(-\frac{(q_H^* - h)^2}{2\Delta\sigma_H^2} - \frac{(q_K^* - k)^2}{2\Delta\sigma_K^2} - \frac{(q_L^* - l)^2}{2\Delta\sigma_L^2}\right) \,. \tag{S3}$$

The total intensity distribution, in a form of X-ray scattering function $S(\mathbf{q}^*)$ is expressed as a linear combination of these local and global structure components:

$$S(\mathbf{q}*) = \sum_{h,k,l,Rot} [AI_{hkl}^{\text{glob}}(\mathbf{q}*, \sigma) + BI_{hkl}^{\text{loc}}(\mathbf{q}*, \Delta\sigma_H, \Delta\sigma_K, \Delta\sigma_L)] + CI_{000}(\mathbf{q}*, \sigma_{bgr}) \,. \tag{S4}$$

In this expression, the term $\sum_{\text{h,k,l,Rot}}$ represents a summation over Miller indices (h, k, l) and over all possible rotational configurations of the local structure. These rotational configurations are captured through the twinning rules (Supplementary Figure 4) which also define the symmetry relationship between local structure configurations (Supplementary Table I). A and B are scaling constants. The term $CI_{000}(\mathbf{q}^*, \sigma_{\text{bgr}})$ represents a 3D Gaussian function centred at the reciprocal space origin, which corrects for imperfections in background subtraction during the X-ray measurement.

It is necessary to distinguish between Miller indices (h, k, l) and related structure factors F_{hkl}^{loc} and F_{hkl}^{glob} that correspond to local and global structure, respectively. This distinction is made by imposing specific conditions that the (h, k, l) triplets must satisfy.

To simulate the local $I4/mcm$ structure within the global $Pm\bar{3}m$ structure, we define the conditions for computing I_{hkl}^{glob} and I_{hkl}^{loc}. The components of I_{hkl}^{glob} contain Miller indices (h, k, l) that satisfy the following condition:

$$(h, k, l) \in \mathbb{Z}^3 \,, \tag{S5}$$

i.e. when Miller indices (h, k, l) are simultaneously integer values. All of the above Miller indices would be precisely contained in the global $Pm\bar{3}m$ structure. I_{hkl}^{loc} components contain Miller indices of the superstructure reflections that would arise when the $Pm\bar{3}m$ structure is transitioning to the $I4/mcm$ structure. The Miller indices of I_{hkl}^{loc} are those that do not satisfy the first condition, i.e., all Miller indices that are not simultaneously integer values.

We have previously established that the local structure symmetry follows the non-merohedral twinning symmetry. Thus, there are three components of the local $I4/mcm$ structure. The following table summarises the symmetry operations that were applied to calculate the total scattering intensity.

TABLE I: Components of the $I4/mcm$ and $P4/mbm$ local structure

Local symmetry	Glazer notation	D_1		D_2	D_3
		I_{hkl}	I_{hkl}^{loc}		
$I4/mcm$, $P4/mbm$	$a^0a^0c_S^-$, $a^0a^0c_S^+$	$(h, k, l) \in \mathbb{Z}^3$	other	$\mathcal{R}(\pi, [10\bar{1}])$	$\mathcal{R}(\pi, [01\bar{1}])$

In this table $\mathcal{R}(\pi, [10\bar{1}])$ denotes that the D_2 component was obtained by the rotation of the D_1 intensity component for 180° around $[10\bar{1}]$ direction in the reciprocal space. The relationship between correlation length ξ_i and $\sigma + \Delta\sigma_i$ can be derived by assuming the full width at half maximum (FWHM) of a Gaussian function is approximately equal to the FWHM of a Lorentzian function, which accurately represents the function arising from short-range order. This approximation is valid in our case (when instrument response broadening is on the order of Lorentzian linewidth)

according to Kielkopf's approximation formula [25] for a FWHM of a Voigt profile. The relationship between ξ_i and FWHM (when FHWM is measured in reciprocal lattice units) is:

$$\xi_i = \frac{2a}{\pi FWHM_i}. \tag{S6}$$

Note that this expression represents the correlation length diameter and is twice the correlation length radius which is obtained as:

$$\xi_i^r = \frac{a}{2\pi HWHM_i}, \tag{S7}$$

where HWHM is half width at half maximum. These expressions show how the correlation length and the width of reciprocal space functions are related. It is also possible to measure correlations in real space. The equivalent expression to Eq. (S7) but in real space is:

$$R(x) = \exp\left(-\frac{x}{\xi_i^r}\right), \tag{S8}$$

where x is a spatial coordinate and $R(x)$ is the spatial tilting correlation function [26]. We used this expression to derive correlation length from MD trajectory analysis in real space.

In Eq. (S6) we can express the FWHM in terms of standard deviation $\Delta\sigma_i$:

$$FWHM_i = 2\sqrt{2\ln 2}(\Delta\sigma_i). \tag{S9}$$

To find the relationship between correlation length ξ_i along certain real space direction and standard deviation $\Delta\sigma_i$ along corresponding reciprocal space direction:

$$\xi_i = \frac{a}{\pi\sqrt{2\ln 2}(\Delta\sigma_i)}. \tag{S10}$$

These three standard deviations $\Delta\sigma_i$ obey the following relationship: For $a^0a^0c^{---}_{LLS}$ ($a^0a^0c^-_S$): $\Delta\sigma_\perp = \Delta\sigma_L$ and $\Delta\sigma_\parallel = \Delta\sigma_H = \Delta\sigma_K$.

We assume that all components of the local structure are equally probable. We inherit the CIF file of the lower symmetry average phase and use those atomic positions to generate X-ray scattering factors of the local structure. It should be noted that this approximation could introduce discrepancies, as the actual degree of local octahedra tilt might not perfectly correspond to the inherited tilt from the low-temperature average phase. This deviation could then manifest as a minor disparity between the simulated structure factor intensities and the experimentally measured values.

TABLE II: Values of the fitting parameters and constants used to determine correlation lengths. $\Delta\sigma_\perp$, $\Delta\sigma_\parallel$, A, B, C and σ_{bgr} are fitting parameters, σ is the standard deviation of the instrument response function, a is the pseudocubic unit cell constant experimentally determined at temperature T and $\xi_\perp$ and $\xi_\parallel$ are correlation lengths determined from fitting parameters.

Sample	T [K]	Glazer Notation	σ [r.l.u.]	$\Delta\sigma_\perp$ [r.l.u.]	$\Delta\sigma_\parallel$ [r.l.u.]	A	B	C	σ_{bgr}	a [Å]	$\xi_\perp$ [Å]	$\xi_\parallel$ [Å]
$MAPbBr_3$	300	$a^0a^0c^-_S$	3e-3	0.256	0.078	8.265	0.021	15.228	1.024	5.941	6.264	20.506
$MAPbBr_3$	200	$a^0a^0c^-_S$	3.5e-3	0.217	0.0706	/	2.83e-5	0.012	/	5.921	7.362	22.657
$FAPbBr_3$	300	$a^0a^0c^+_S$	4e-3	0.114	0.0756	/	5e-4	1.136	100.0685	6.007	14.21	21.484
$FAPbBr_3$	200	$a^+a^+a^+$	6.4e-3	0	0	1	1	0	/	5.958	∞	∞

To finally estimate the correlation lengths of the local structure, we fit the experimental quasi-elastic diffuse scattering (QEDS) data to Eq. (S4). The obtained fitting parameters are shown in Supplementary Table II. We assess the quality of our fitting by first calculating 95 % confidence intervals of the fitting parameters. The standard error is determined by dividing the range of the confidence intervals by Z-score, which is for 95 % confidence level assuming normal distribution approximately 1.96. The standard errors are given in Supplementary Table III.

We also measured the resolution in Q-space by estimating the broadening of Bragg peaks as presented in Supplementary Table II in the column under σ . As the ΔQ was much smaller than the broadening of the diffuse peaks, we

TABLE III: Standard errors of the fitting parameters and derived correlation lengths.

Sample	T[K]	$\Delta\sigma_\perp$ [$r.l.u.$]	$\Delta\sigma_\parallel$ [$r.l.u.$]	A	B	C	σ_{bgr}	$\xi_\perp$ [Å]	$\xi_\parallel$ [Å]
$MAPbBr_3$	300	1.4e-3	4.6e-4	41.5e-2	1.2e-4	2.412	5e-2	3.5e-2	0.121
$MAPbBr_3$	200	1.1e-3	3.45e-4	/	1e-7	8e-5	/	0.11	0.03
$FAPbBr_3$	300	1.1e-3	7.5e-4	/	5.2e-6	8e-3	744.520	0.142	0.215
$FAPbBr_3$	200	/	/	/	/	/	/	/	/

did not convolve our model with a Gaussian function corresponding to the Q-resolution function. This approach is valid, as ΔQ is two orders of magnitude smaller than the FWHM of the diffuse scattering peaks. Performing such a convolution would make our model significantly more computationally expensive.

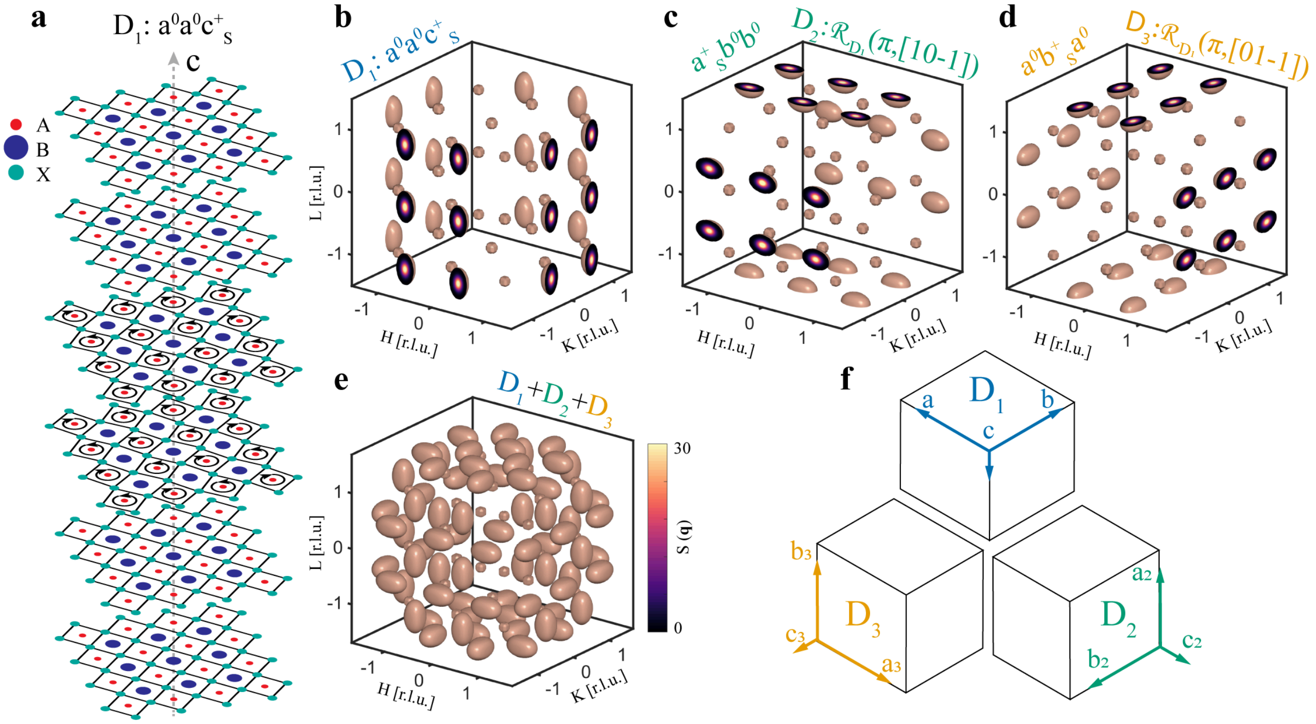

Supplementary Figure 5: **Components of local in-phase octahedral tilting with $P4/mbm$ symmetry in real and reciprocal space.** (**a**) Real-space depiction of the D_1 component of in-phase perovskite ABX_3 octahedral local structure, designated as $a^0a^0c^+_S$ in modified Glazer notation. The octahedra display non-zero tilt along the c axis, with short-range in-phase ordering (one $P4/mbm$ c axis unit) along c and long-range ordering along a and b. (**b**) Computed 3D volumetric X-ray scattering intensities in the reciprocal space of the structure in (**a**) represent the D_1 diffuse scattering component. (**c**) The reciprocal space of the second local structure component (D_2) is derived by rotating (D_1) in (**b**) 180° around the $[10\bar{1}]$ vector of the D_1 coordinate system. (**d**) The reciprocal space of the third local structure component (D_3) is achieved by rotating (D_1) in (**b**) 180° around the $[01\bar{1}]$ vector of the D_1 coordinate system. (**e**) The complete 3D diffuse scattering signal is the cumulative sum of all three components. (**f**) In real space, the local structure is characterized as the combination of three distinct local $P4/mbm$ planar components. The thickness of these components is determined by the degree of short-range anti-phase local octahedral tilting along the c axes of the local coordinate system for each component. The relationship between the axes is demonstrated in (**f**).

b. Local structure in the average tetragonal phase.

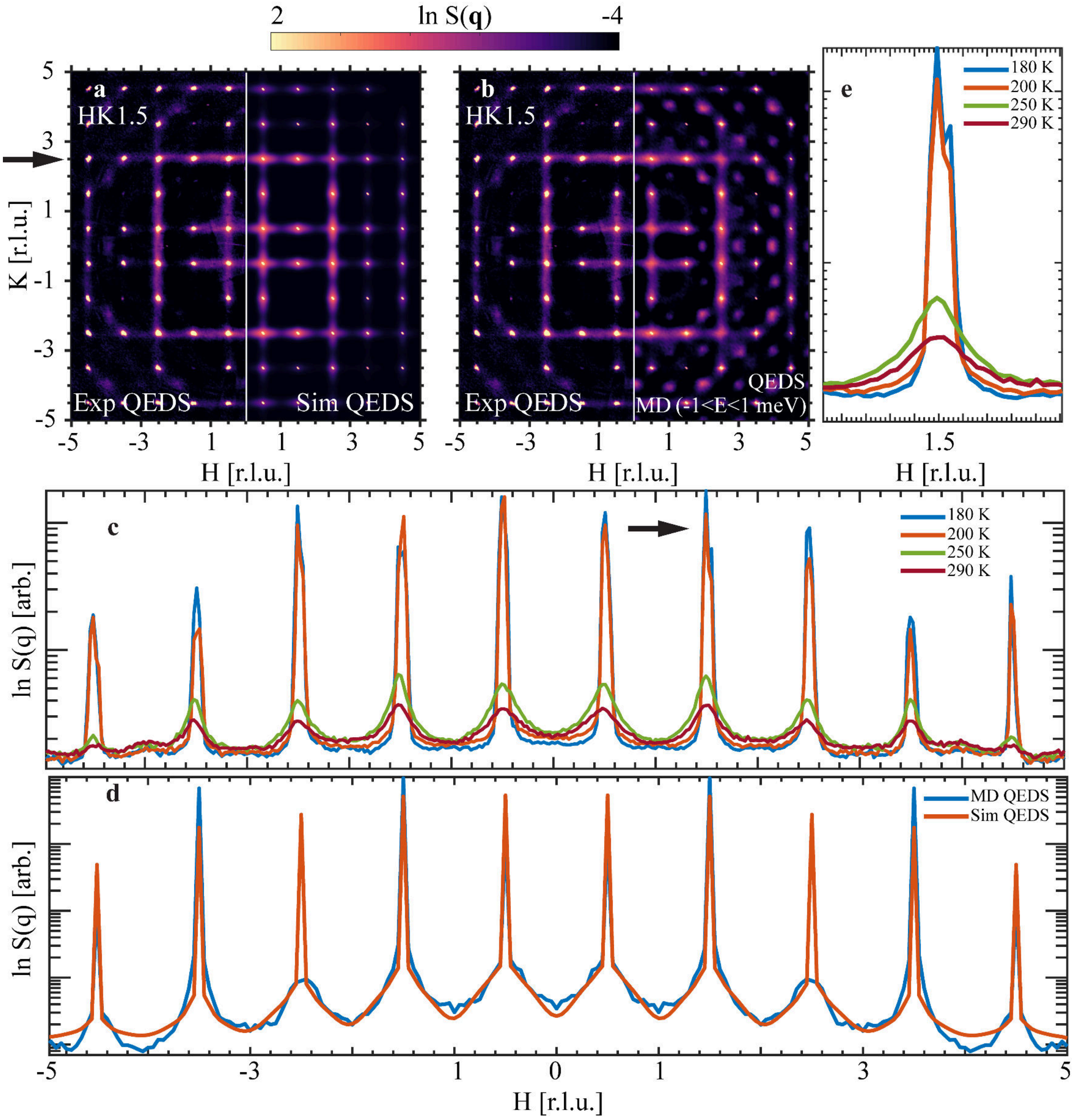

Supplementary Figure 6: **Local structure in the average tetragonal phase of $\mathbf{MAPbBr_3}$** (**a**) Experimental QEDS (left panel) at 200 K and simulated with a phenomenological model (right panel) $S(\mathbf{q})$ across the HK1.5 reciprocal space plane show great agreement. (**b**) Experimental QEDS $S(\mathbf{q})$ (left panel) and MD simulated $S(\mathbf{q}, E)$ integrated over $-1 < E < 1$ meV to obtain QEDS (right panel) across the HK1.5 reciprocal space plane at 200 K also show good agreement. (**c**) At $K = 2.5$ (indicated with black arrow in (**a**)), a cross-section of the experimental QEDS $S(\mathbf{q})$ at various temperatures is taken along the diffuse scattering rods. The horizontal arrow indicates the peaks at $H = 1.5$, which are outlined for clarity in (**e**). (**d**) Horizontal cross-section of MD QEDS $S(\mathbf{q})$ in the HK1.5 plane at $K = 2.5$, with corresponding fit (red line) used to extract correlation lengths of the local structure in the average tetragonal phase.

Surprisingly, we observe that the average tetragonal $I4/mcm$ phase with Glazer notation $a^0a^0c^-$ also exhibits a local structure of the same symmetry as the cubic phase, denoted by $a^0a^0c^-_S$. This implies that, while the c-axis tilts in $a^0a^0c^-$ are expected to be infinitely correlated along the c axis, they in fact exhibit short-range correlations of zero tilt. The equivalence in symmetry between the local and average structure is evident in the experimental QEDS data for $MAPbBr_3$ at 200 K, shown in the left panel of Supplementary Figure 6 **a**. Specifically, broad diffuse scattering peaks now align with sharp Bragg superstructure peaks that emerge during the cubic-tetragonal phase transition in the $HK1.5$ plane. This is confirmed in the right panel of Supplementary Figure 6 **a** through our phenomenological model, which shows excellent agreement between experimental and simulated data. Further support for this hypothesis comes from QEDS $S(\mathbf{q})$ calculations at 200 K derived from MD trajectories, which align well with experimental data (Supplementary Figure 6 **b**). Supplementary Figure 6 **c** presents horizontal cuts of experimental QEDS $S(q)$ at the $HK1.5$ plane, at $K = 2.5$ over various temperatures. The progressive sharpening of diffuse peaks at half-integer H positions (indicative of progressive increase of correlation lengths of dynamic nanodomains) into Bragg peaks below 235 K is clearly visible. Below 235 K, these Bragg peaks still exhibit background diffuse broadening within the average tetragonal phase, as shown in Supplementary Figure 6 **e**, where we highlight the peak at $H = 1.5$, $K = 2.5$, $L = 1.5$. This narrowing of the background diffuse peaks underneath the main Bragg peaks intensifies with cooling, as evident at 180 K. Since the diffuse peak's intensity is weak compared to the main Bragg peak, extracting correlation lengths directly from experimental data was unfeasible. However, this was achieved using MD data, which allowed us to estimate the parallel and normal correlations in the tetragonal phase at 200 K as 22.65 and 7.36 Å, respectively. The fitting parameters used for this MD data at 200 K are provided in Supplementary Table II. Cross-sectional horizontal cuts of the MD QEDS $S(q)$ and corresponding fits used to derive correlation lengths are shown in Supplementary Figure 6 **d**.

c. Comparison between experimental diffuse scattering patterns and those generated by the phenomenological model.

The presence of additional diffracted intensities within the half-integer 2D reciprocal space planes (Supplementary Figure 7) signifies a unit cell doubling, a characteristic indicative of a reduction in symmetry. In that context, the diffuse scattering rods and ellipsoids form due to broadened superstructure peaks within reciprocal space, with the degree of broadening serving as a metric for local order.

To accurately determine the symmetry of the local structure, it is crucial to fully understand the symmetry inherent to the diffuse scattering pattern. We establish that the symmetry of the Quasi-Elastic Diffuse Scattering (QEDS) within the average cubic phases of these materials mirrors the symmetry of the Bragg peaks (Laue symmetry) found in their average tetragonal phases. This Laue symmetry of the average tetragonal structure is however higher than the inherent symmetry of the crystal, which is common in the case of non-merohedral twinning (for details see Supplementary Note 3).

To test our phenomenological model we compare experimental and simulated QEDS $S(\mathbf{q})$ across $HK1.5$, $HK0.5$, $HK0$ reciprocal space planes as shown in Supplementary Figure 7, Supplementary Figure 8 and Supplementary Figure 9.

In Supplementary Figure 9 **a** and **b** we present experimental total diffuse scattering (left panels) and simulated QEDS (right panels) signals across HK0 reciprocal space planes in $MAPbBr_3$ and $FAPbBr_3$, respectively. It is evident that the QEDS simulation does not reproduce the rods of diffuse scattering linking the Bragg peaks in Supplementary Figure 9 **a**. This outcome is anticipated since the simulation excludes the TDS, which is responsible for these rods. Nonetheless, the QEDS simulation successfully identifies the emergence of additional broad reflections at the M points in reciprocal space, highlighted by white circles in Supplementary Figure 9 **a**, **b** and **c**. These QEDS features in the HK0 plane arise from the cross sections of R-M diffuse rods within the e.g. H0.5L and H1.5L planes, that are perpendicular to HK0 (note that all set of all perpendicular planes such as e.g. HKn, HnL 0Kn, where n are integer and half-integer values, are always identical in the average cubic phase even in the presence of diffuse scattering due to effective twinning of the dynamic local nanodomains as explained in detail in the main text). Conversely, the MD simulation depicted in Supplementary Figure 13 **e** and **f** encompasses both TDS and QEDS, thereby accurately mirroring the complete experimental diffuse scattering observed in the HK0 planes.

We quantitatively test our phenomenological model on several selected reciprocal space planes in $MAPbBr_3$ and $FAPbBr_3$ as shown in Supplementary Figure 10 and Supplementary Figure 11, respectively. The information about $\xi_\perp$ is predominantly captured in the horizontal cross-section of the diffuse rods across the R-M direction in the Brillouin

zone. Conversely, $\xi_{\parallel}$ is discerned from the vertical cross-section of these rods. Thus, in $MAPbBr_3$ we present a 1D cross-section of the 2D $S(\mathbf{q})$ HK1.5 at K = 2.5 (along the rod) and K = 1.5 (vertical to the rod), in Supplementary Figure 10 **e**. We observe great agreement between the experiment and simulation, which implies that the correlation length values we derive are accurate. The same is true for HK0.5 plane. Similar consideration can be found for $FAPbBr_3$ in e.g. Supplementary Figure 11 **e**, which shows 1D cross sections at certain K values of the HK1.5 plane. While the peaks in $FAPbBr_3$ are more isotropic, we can still compare cross sections along the rod $K = 3.5$ and vertical to the rod $K = 1.5$. The agreement between the simulation and experiment gives confidence in the accuracy of our estimated correlation lengths.

We conducted two independent experiments to characterise the local structure of $FAPbBr_3$ at 300 K. We used both laboratory X-ray source (CuKα radiation to avoid fluorescence from bromine) and two synchrotron radiation sources to verify that in both cases we observe the same diffuse scattering patterns. The results are presented in Supplementary Figure 12.

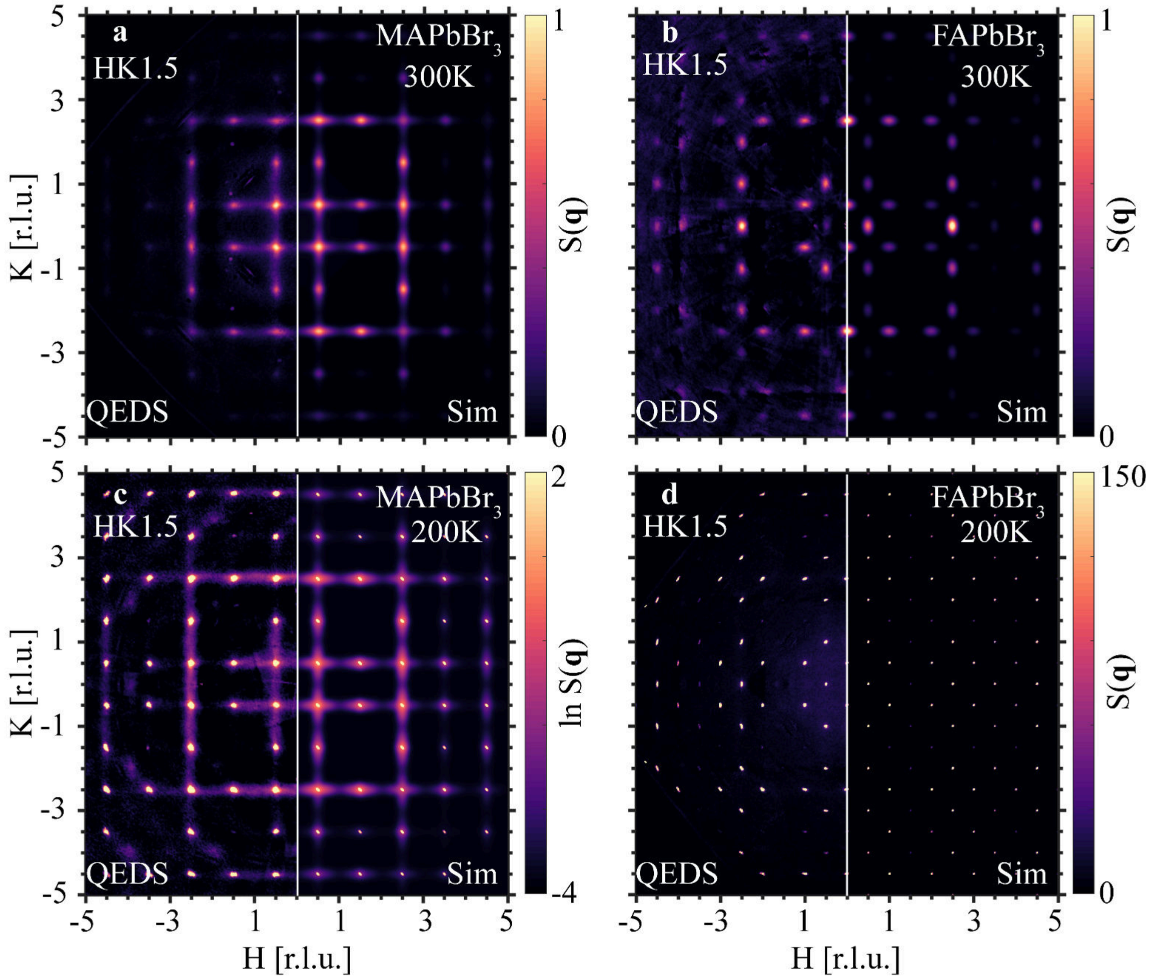

Supplementary Figure 7: **Relationship between phase transitions and diffuse scattering in HK1.5 planes.** Experimental QEDS (left panels) and simulated (right panels) $S(\mathbf{q})$ across HK1.5 reciprocal space planes show great agreement. For simulation we used a phenomenological model. (**a**) $S(\mathbf{q})$ of $MAPbBr_3$ HK1.5 plane and (**b**) $FAPbBr_3$ at $T = 300\,\text{K}$ in average cubic $Pm\bar{3}m$ phase but with local planar $I4/mcm$ and spherical $P4/mbm$ nanodomains, respectively. (**c**) $S(\mathbf{q})$ of $MAPbBr_3$ HK1.5 plane and (**d**) $FAPbBr_3$ at $T = 200\,\text{K}$ in average tetragonal $I4/mcm$ (with local $I4/mcm$ nanodomains) and average $Im\bar{3}$ phase, respectively. The broad QEDS peaks in (**a**) and (**b**) narrow down to Bragg peaks in (**c**) and (**d**) upon the phase transition.

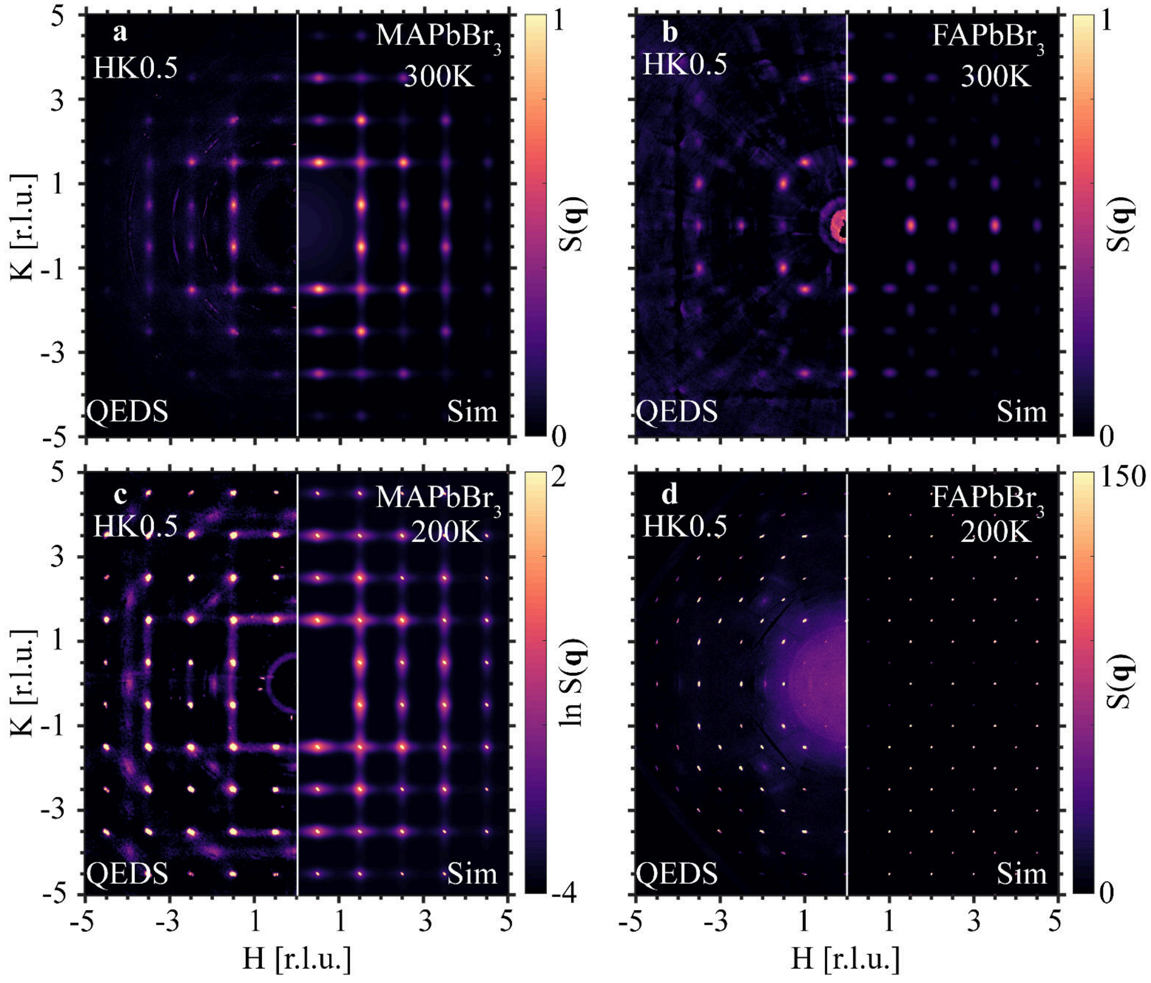

Supplementary Figure 8: **Relationship between phase transitions and diffuse scattering in HK0.5 planes.** Experimental QEDS (left panels) and simulated (right panels) signals across HK0.5 reciprocal space planes. For simulation we used a phenomenological model. (**a**) and (**b**) $MAPbBr_3$ and $FAPbBr_3$ at $T = 300\,K$ in average cubic $Pm\bar{3}m$ phase but with local planar $I4/mcm$ and spherical $P4/mbm$ nanodomains, respectively. (**c**) and (**d**) $MAPbBr_3$ and $FAPbBr_3$ at $T = 200\,K$ in average tetragonal $I4/mcm$ (with local $Pnma$ nanodomains) and average $Im\bar{3}$ phase, respectively. The broad QEDS peaks in (**a**) and (**b**) narrow down to Bragg peaks in (**c**) and (**d**) upon the phase transition.

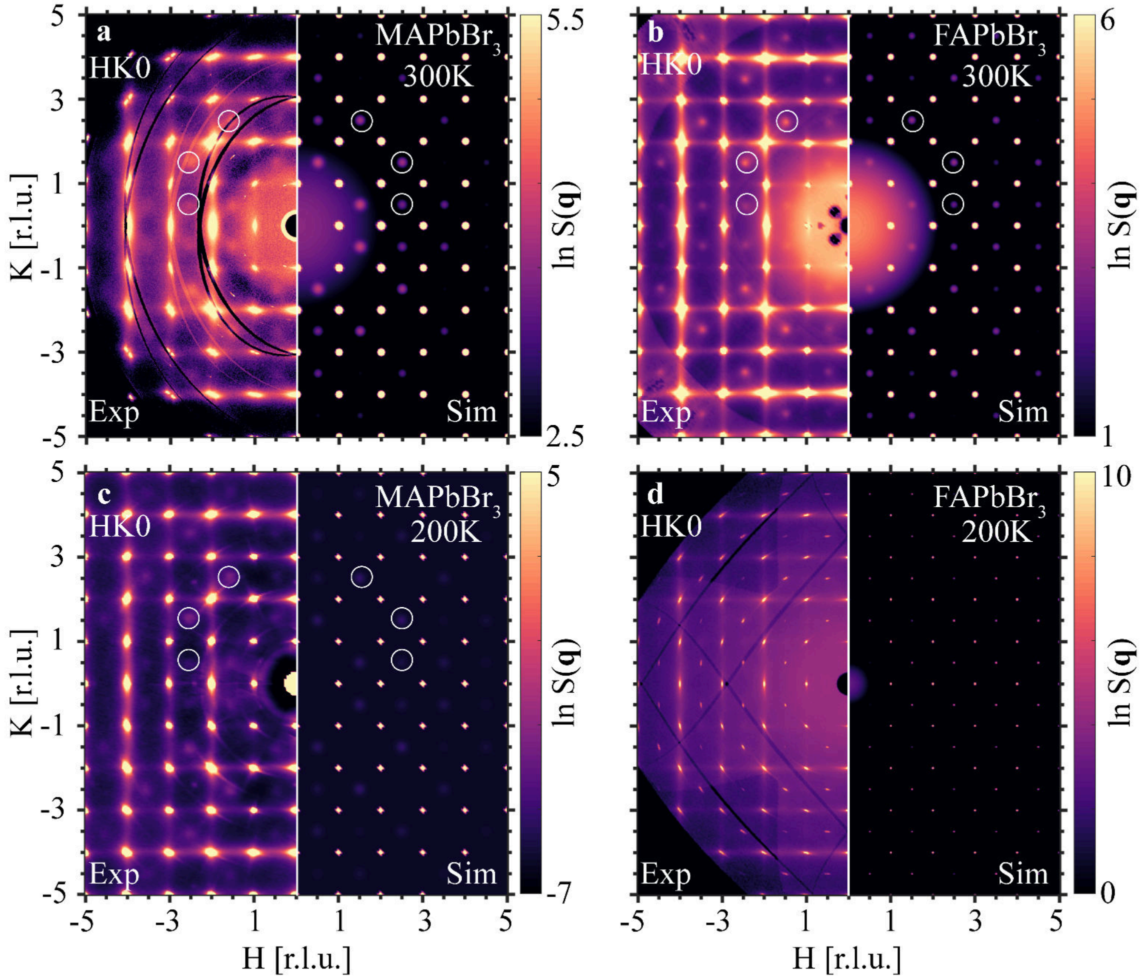

Supplementary Figure 9: **Relationship between phase transitions and diffuse scattering in HK0 planes.** For simulation we used a phenomenological model. Experimental total diffuse scattering (left panels) and simulated QEDS (right panels) signals across HK0 reciprocal space planes in $MAPbBr_3$ and $FAPbBr_3$ are shown in (**a**)-(**d**). Our simulations do not capture TDS signals that are present in HK0, however, the broadened QEDS superstructure reflections are captured in (**a**)-(**d**). Diffuse superstructure reflections at 300 K at M points in the Brillouin zone are circled in (**a**), (**b**) and (**c**).

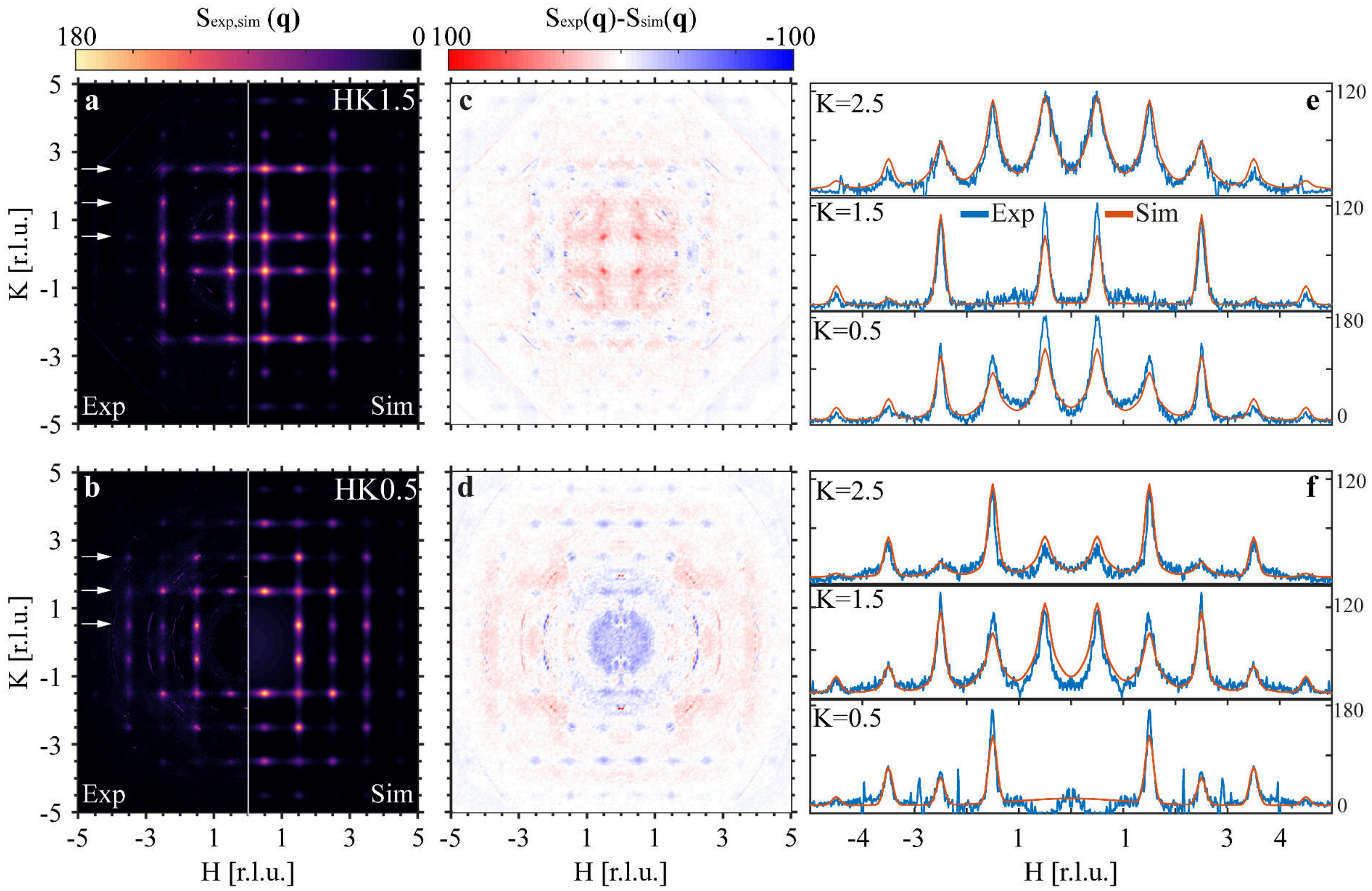

Supplementary Figure 10: **Comparison of experimental and modelled quasi-elastic diffuse scattering signals in $\mathbf{MAPbBr_3}$ at $T = 300\,\mathrm{K}$.** For simulation we used a phenomenological model. The panels on the left represent experimental scattering functions $S_{\mathrm{exp}}(\mathbf{q})$ and those on the right illustrate the simulated scattering function $S_{\mathrm{sim}}(\mathbf{q})$ for the $HK1.5$ (**a**) and HK0.5 (**b**) reciprocal space planes of $MAPbBr_3$. The difference between the experimental and simulated data for HK1.5, HK0.5 reciprocal space planes is displayed in (**c**) and (**d**), respectively. The simulation data was obtained by fitting the phenomenological model to the experimental data in the $HK1.5$ plane, achieving a root mean square (RMS) value of 5.072% and 6.17% relative to the maximum of the experimental data in $HK1.5$ and $HK0.5$ planes, respectively. Cross sections along H at constant $K = 2.5$, $K = 1.5$ and $K = 0$ in $HK1.5$ (**e**), HK0.5 (**f**) reciprocal space planes. The arrows in (**a**) and (**b**) indicate the directions of the displayed cross sections.

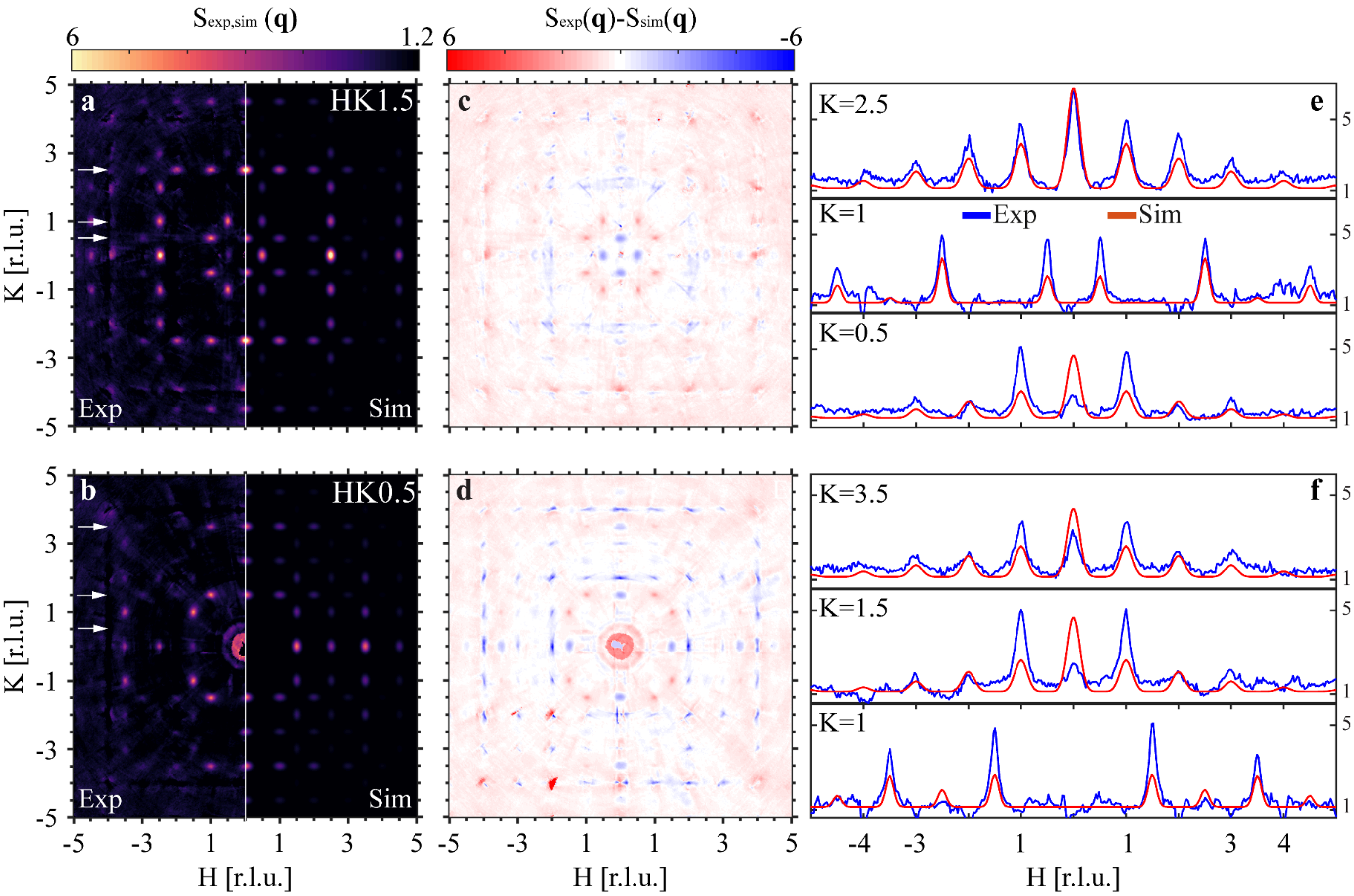

Supplementary Figure 11: **Comparison of experimental and modelled quasi-elastic diffuse scattering signals in $FAPbBr_3$ at $T = 300\,K$.** For simulation we used a phenomenological model. The panels on the left represent experimental scattering function $S_{\mathrm{exp}}(\mathbf{q})$ and those on the right illustrate the simulated scattering function $S_{\mathrm{sim}}(\mathbf{q})$ for the $HK1.5$ (**a**) and HK0.5 (**b**) reciprocal space planes of $FAPbBr_3$. The difference between the experimental and simulated data for HK1.5, HK0.5 reciprocal space planes is displayed in (**c**) and (**d**), respectively. The simulation data was obtained by fitting the phenomenological model to the experimental data in the $HK1.5$ plane, achieving a root mean square (RMS) value of 15.872% relative to the maximum of the experimental data in $HK1.5$ plane. Cross sections along H at constant K values in $HK1.5$ (**e**), HK0.5 (**f**) reciprocal space planes. The arrows in (**a**) and (**b**) indicate the directions of the displayed cross sections.

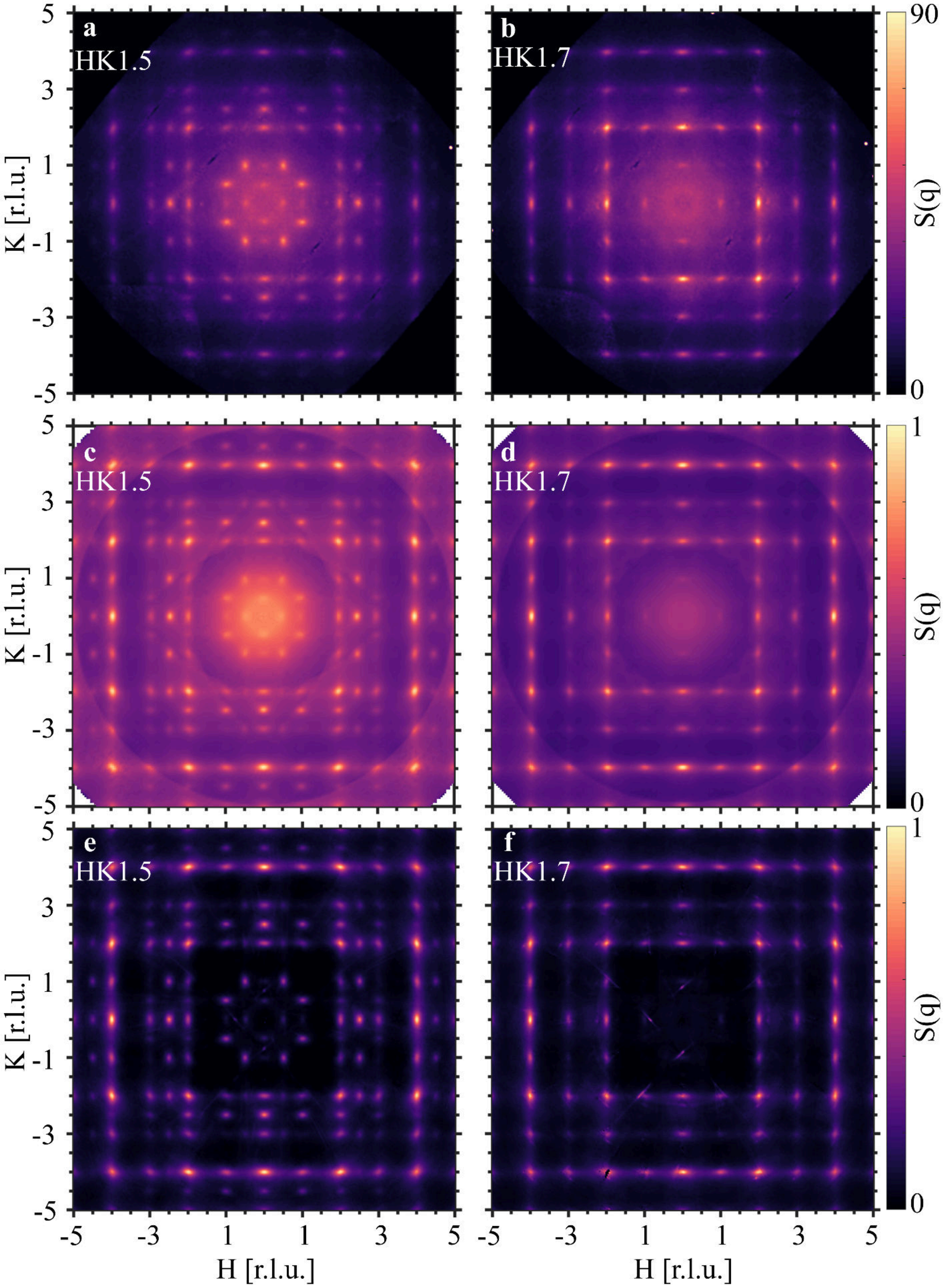

Supplementary Figure 12: **Three independent measurements confirm the presence of the same diffuse scattering patterns in $FAPbBr_3$ at** 300 **K.** (**a**) and (**b**), show $S(\mathbf{q})$ of HK1.5 and HK1.7 reciprocal space planes, respectively, obtained using synchrotron radiation at MX1 at Australian Synchrotron. (**c**) and (**d**), show $S(\mathbf{q})$ of HK1.5 and HK1.7 reciprocal space planes, respectively, obtained using a laboratory X-ray source (CuKα radiation). (**e**) and (**f**), show $S(\mathbf{q})$ of HK1.5 and HK1.7 reciprocal space planes, respectively, obtained using synchrotron radiation at P21.1 at Petra III, DESY.

6. MOLECULAR DYNAMIC SIMULATIONS

a. Comparison of experimental and MD computed diffuse scattering patterns

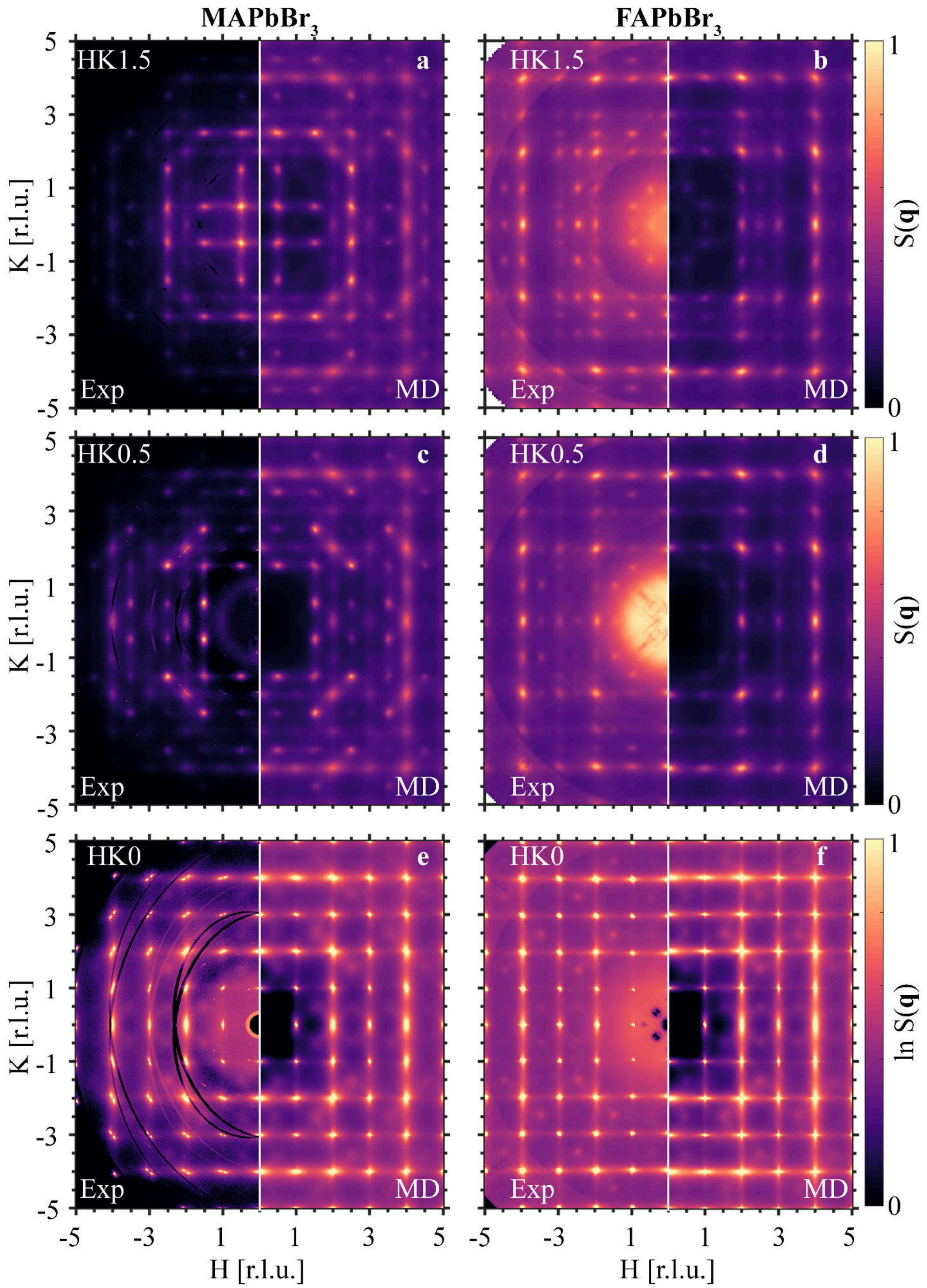

Supplementary Figure 13: **Comparison of experimental and MD computed diffuse scattering in HK**1.5 **planes.** Total experimental (left panels) and total MD simulated (right panels) $S(\mathbf{q})$ signals across HK1.5 reciprocal space planes show great agreement, in both $MAPbBr_3$ and $FAPbBr_3$. (**a**) and (**b**) comparison of experimental and MD $S(\mathbf{q})$ in HK1.5 in $MAPbBr_3$ and $FAPbBr_3$ at $T = 300\,K$, respectively. (**c**) and (**d**) comparison of experimental and MD $S(\mathbf{q})$ in HK0.5 in $MAPbBr_3$ and $FAPbBr_3$ at $T = 300\,K$, respectively. (**e**) and (**f**) comparison of experimental and MD $S(\mathbf{q})$ in HK0 in $MAPbBr_3$ and $FAPbBr_3$ at $T = 300\,K$, respectively. Note that $MAPbBr_3$ experimental data presented here is from MX1 at Australian Synchrotron and $FAPbBr_3$ data is collected at the University of Oxford. For a fairer comparison with the experimental diffuse scattering patterns at 300 K, MD simulations were performed at 340 K for $MAPbBr_3$ and 250 K for $FAPbBr_3$; see Supplementary Note 8 for further details.

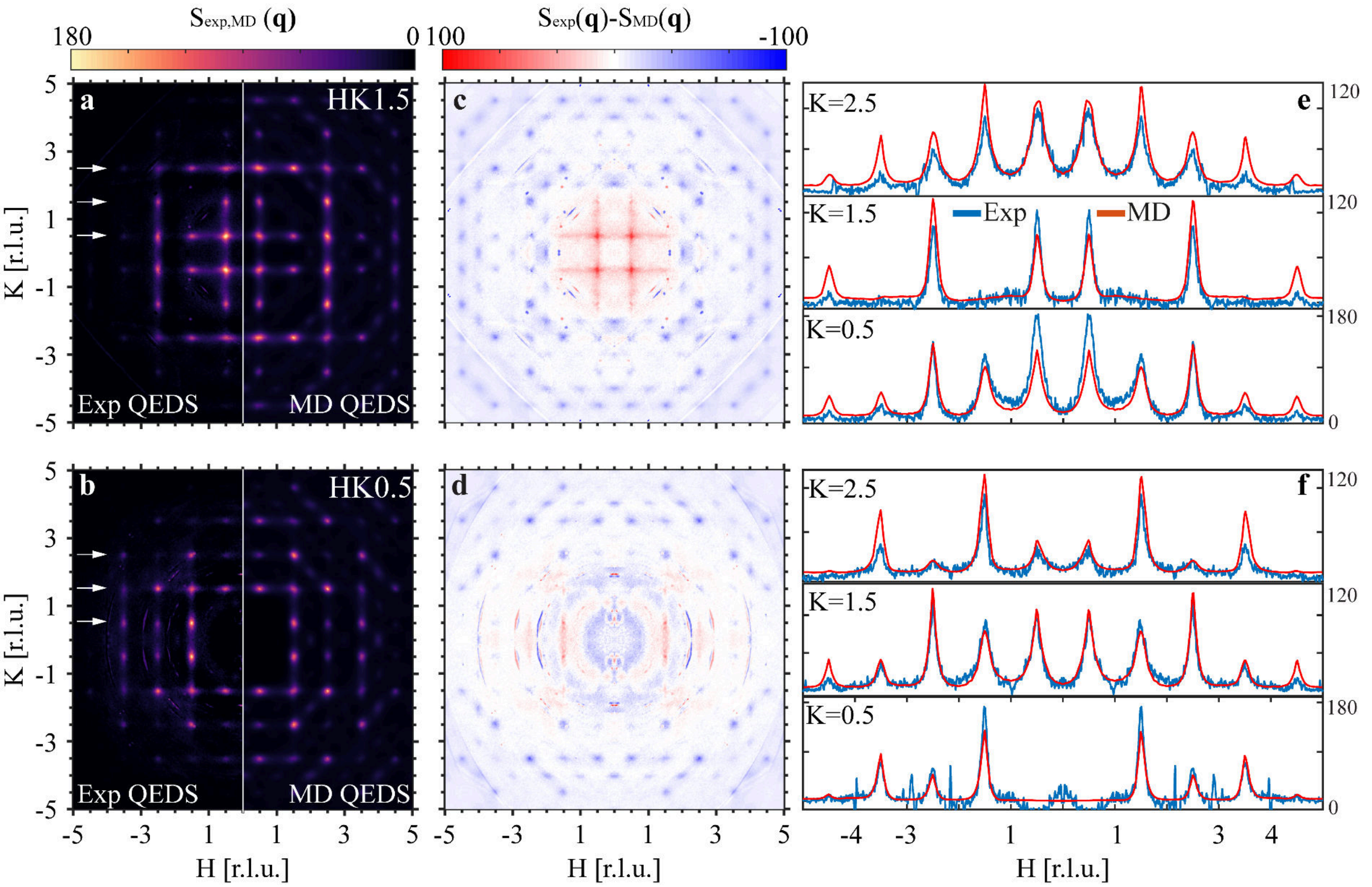

Supplementary Figure 14: **Comparison of experimental and MD computed QEDS in HK1.5 planes of $\mathbf{MAPbBr_3}$.** The panels on the left represent experimental QEDS scattering function $S_{\rm exp}(\mathbf{q})$ and those on the right illustrate the MD simulated QEDS scattering function $S_{\rm MD}(\mathbf{q})$ for the $HK1.5$ (**a**) and HK0.5 (**b**) reciprocal space planes of $MAPbBr_3$. The difference between the experimental and simulated data for HK1.5, HK0.5 reciprocal space planes is displayed in (**c**) and (**d**), respectively. Cross sections along H at constant K values in $HK1.5$ (**e**), HK0.5 (**f**) reciprocal space planes. The arrows in (**a**) and (**b**) indicate the directions of the displayed horizontal cross-sections. A root mean square (RMS) values of 5.54% and 5.48% relative to the maximum of HK1.5 and HK0.5 experimental data, respectively, indicate a great agreement between MD simulation and experiment. For a fairer comparison with the experimental diffuse scattering patterns at 300 K, MD simulations were performed at 340 K; see Supplementary Note 8 for further details.

b. The origin of quasi-elastic diffuse scattering at X points

In Supplementary Figure 15 **a-d**, we present total MD-simulated $S(\mathbf{q})$ ($S(\mathbf{q}, E)$ integrated over full E range) across two planes, HK1 and HK1.5, for $MAPbBr_3$ and $FAPbBr_3$ at 300 K, highlighting high-symmetry points across the Brillouin zones. To encompass all high-symmetry points, it is necessary to consider two parallel 2D planes separated by 0.5 reciprocal lattice units (r.l.u.), as depicted in Supplementary Figure 15 **e**, which illustrates the first Brillouin zone of a pseudocubic real unit cell. We determine that the quasi-elastic diffuse scattering (QEDS) in $MAPbBr_3$ originates from the broadened superstructure diffuse peaks at R-points, whereas in $FAPbBr_3$, these peaks are located at M-points, as shown in Supplementary Figure 15 **a-d**. Zone-edge transverse acoustic phonons at R-points are characterised by pure out-of-phase octahedral tilt modes, while at M-points, they indicate pure in-phase octahedral tilts, as illustrated in Supplementary Figure 15 **e**. These findings align with our conclusion that the local structure in $MAPbBr_3$ and $FAPbBr_3$, within the average cubic phase, consists of local $I4/mcm$ and local $P4/mbm$ phases, respectively.

In the Main Text Fig.1 MD simulations reveal that QEDS in HK1.5 planes of $MAPbBr_3$ and $FAPbBr_3$, energy-integrated from -1 to 1 meV shows pronounced R and M-point peaks. It is also observed that the patterns include

X-point peaks, which are significantly more pronounced in $FAPbBr_3$ than in $MAPbBr_3$. As we established that dynamic local nanodomains of lower symmetry fluctuating in higher symmetry phase scatter quasi-elastically with X-rays and give rise to R and M QEDS peaks, this would imply that there is also additional local order that gives rise to X-point QEDS scattering. To elucidate the origins of this X-point QEDS signal, we have performed an integration of the MD-computed $S(\mathbf{q}, E)$ over a narrow energy range from 0 to 0.05 meV, as illustrated in Supplementary Figure 16, to primarily capture the elastic scattering component. In Supplementary Figure 16 **b** and **d** we observe the same diffuse patterns as in Main Text Fig.1 **a** and **b**, bottom right quadrants, confirming that the X scattering has a pure elastic component.

In the H0L planes, as illustrated in Supplementary Figure 16 panels **a** and **c**, we observe rods of nearly elastic scattering originating from Γ points within the Brillouin zone (at Bragg peaks) extending along the $\Gamma - \mathrm{X} - \Gamma$ direction. Given that H0L planes intersect perpendicularly with HK1.5 planes, these $\Gamma - \mathrm{X} - \Gamma$ rods intersect the latter at X points, thereby creating observable peaks at these intersections, which are effectively cross-sections of the rods.

By examining two q-cross-sections in H0L centered at the (020) reflection, one vertical in a direction $[0+q, 2, 0]$ and the other horizontal in a direction $[0, 2+q, 0]$ (the directions are marked in Supplementary Figure 17 **b**) and plotting the corresponding $S(\mathbf{q}, E)$ for each, we observe distinct phonon dispersions presented in Supplementary Figure 17 **a** and Supplementary Figure 17 **e**, respectively. The vertical cross-section reveals the dispersion of longitudinal acoustic (LA) phonons, as shown in Supplementary Figure 17 **a**,while the horizontal cross-section exposes the dispersion of transverse acoustic (TA) phonons, as illustrated in Supplementary Figure 17 **b**. This distinction arises from the polarization selection rules in $S(\mathbf{q}, E)$, where the vertical cross-section, being longitudinal to the (020) Bragg peak, contains information solely about LA phonons. Conversely, the horizontal direction is associated with TA phonons.

As observed in Supplementary Figure 17 **b**, $\Gamma - \mathrm{X} - \Gamma$ rods emerge along the transverse directions. By integrating the $S(\mathbf{q}, E)$ over an energy range from 0 to 0.05 meV for both vertical and horizontal directions, we observe that while Bragg peaks at integer q values are sharp in the vertical direction, they exhibit broadening in the horizontal (transverse) direction, as shown in Supplementary Figure 17 **d**. The broadened Bragg peaks in the transverse direction create a 'valley' between them which we observe as rods of diffuse scattering along $\Gamma - \mathrm{X} - \Gamma$ trajectory. Let us now understand what causes the broadening of these Bragg peaks in q along the transverse direction. By definition, TA phonons are lower energy excitations compared to LA phonons. Thus if we select a very low energy near the zone centre like E_{min} in Supplementary Figure 17 **c**, the corresponding wavevector q of TA phonons is always going to be larger than q of LA phonons for the same E_{min}. As seen in Supplementary Figure 17 **d**, at Brillouin zone centre we expect a very sharp Bragg peak (as it corresponds to infinite correlations of atoms) and a contribution from low energy zone centre acoustic phonons. For TA phonons, the phonon peak will be displaced further away from the adjacent Bragg peaks compared to LA phonons, thus the Bragg peaks will appear as more broadened in the transverse direction. Consequently, zone centre TA phonons invariably broaden Bragg peaks more than LA phonons, leading to the formation of rods in the transverse direction. So, to conclude, zone centre acoustic phonons are responsible for the QEDS $\Gamma - \mathrm{X} - \Gamma$ rods and as a result, also are responsible for the emergence of QEDS X-point peaks in HK1.5 planes. While our analysis has elucidated the emergence of X point peaks using $MAPbBr_3$ as an example, this phenomenon is similarly observed in $FAPbBr_3$. It is important to highlight that $FAPbBr_3$ exhibits softer phonons compared to $MAPbBr_3$ (i.e. LA and TA phonons in $FAPbBr_3$ are always lower in energy than in $MAPbBr_3$), leading to a more pronounced broadening of Bragg peaks along the transverse direction. This can be clearly observed by comparing Supplementary Figure 16 **a** and **b**, where $\Gamma - \mathrm{X} - \Gamma$ rods are more pronounced in $FAPbBr_3$ than in $MAPbBr_3$. Consequently, this results in higher intensity X point peaks in $FAPbBr_3$, at HK1.5 planes.

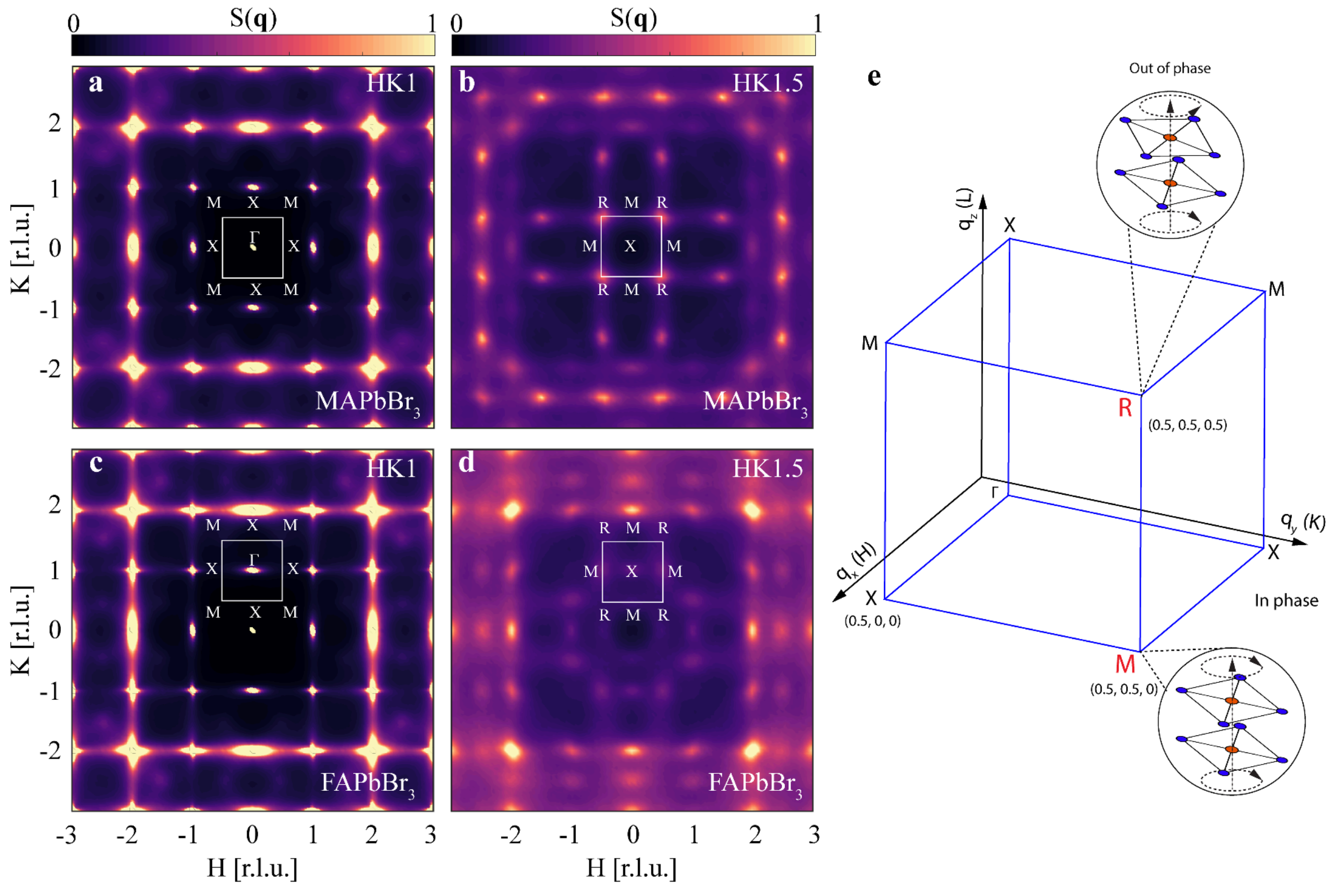

Supplementary Figure 15: **Visualization of high-symmetry points in the Brillouin zone and their relationship with diffuse scattering in halide perovskites.** (**a**) and (**b**) MD-simulated $S(\mathbf{q})$ across the HK1 and HK1.5 planes in $MAPbBr_3$ at 300 K, showing high-symmetry points across four Brillouin zones centered at (001). This visualization highlights the relationship between diffuse scattering and high-symmetry points. (**c**) and (**d**) MD-simulated $S(\mathbf{q})$ across the HK1 and HK1.5 planes in $FAPbBr_3$ at 300 K, showing high-symmetry points across four Brillouin zones centered at (011). (**e**) 3D representation of the first Brillouin zone of a pseudocubic real unit cell. In halide perovskites, zone-edge acoustic phonon modes at R-points correspond to pure out-of-phase tilts along the c-axis, whereas at M-points, they correspond to pure in-phase tilts along the c-axis.

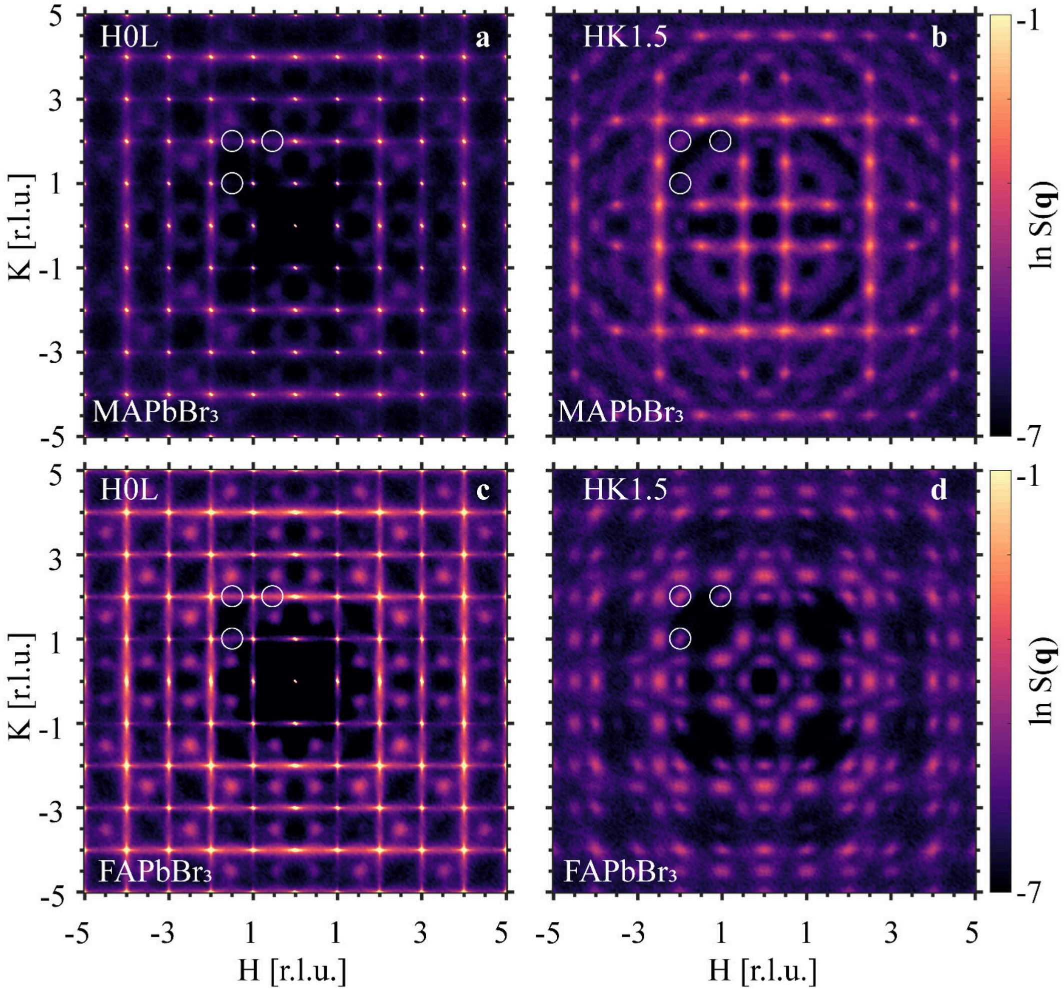

Supplementary Figure 16: **MD simulated $S(\mathbf{q})$ obtained by integrating $S(\mathbf{q}, E)$ over $0 < E < 0.05\,$meV.** (**a**) and (**b**) $S(\mathbf{q}, E)$ across H0L and HK1.5, respectively, integrated over $0 < E < 0.05\,$meV, for $MAPbBr_3$ at 300 K. (**c**) and (**d**) $S(\mathbf{q}, E)$ across H0L and HK1.5, respectively, integrated over $0 < E < 0.05\,$meV for $FAPbBr_3$ at 300 K. X points across the Brillouin zones are denoted with white circles.

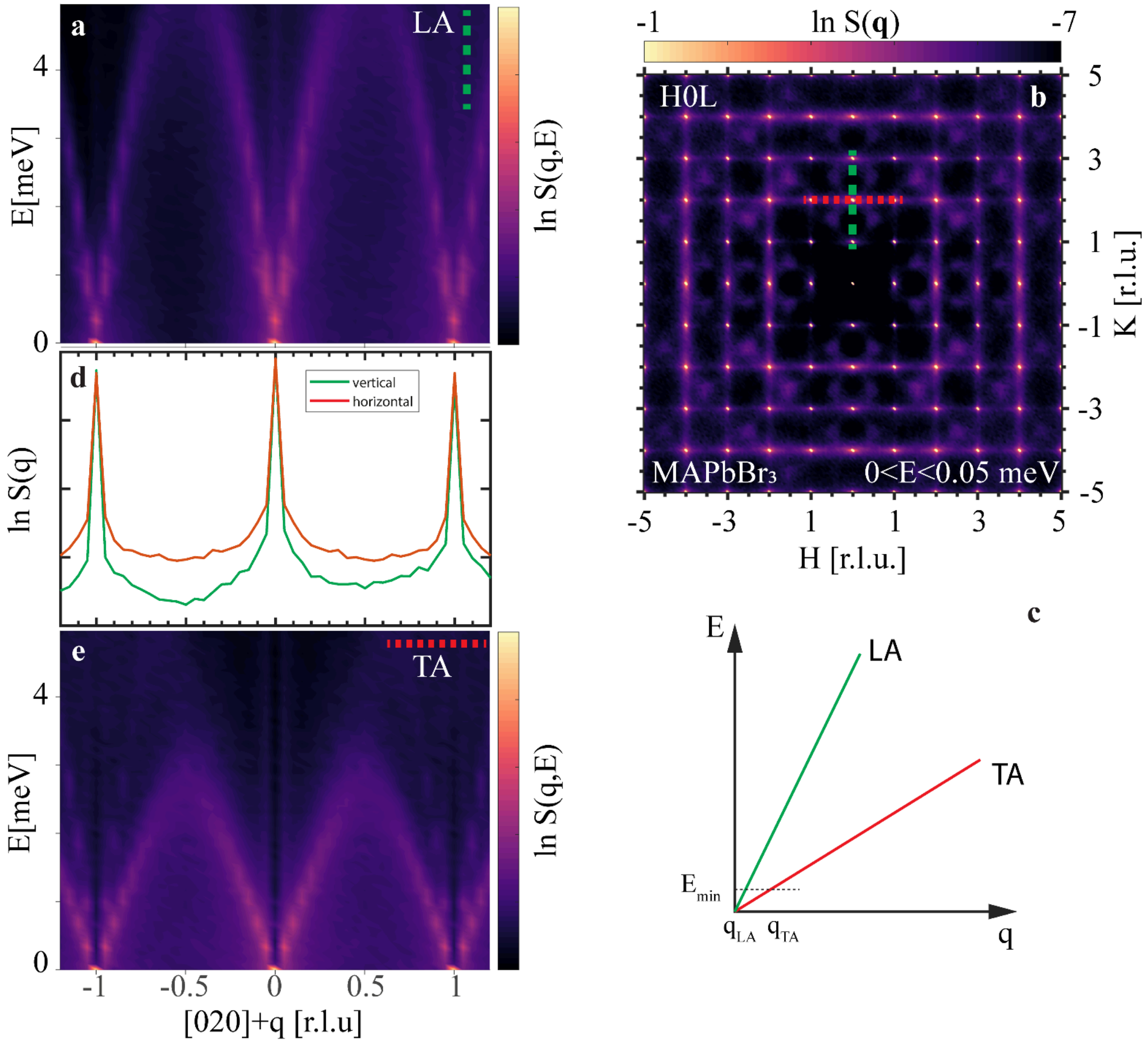

Supplementary Figure 17: **The origin of the QEDS scattering in H0L plane demonstrated on the example of MD $MAPbBr_3$ $S(\mathbf{q}, E)$ data.** (**a**) $S(q, E)$ where q is along $[0+q, 2, 0]$ direction. This map reveals the dispersion of LA phonons. (**e**) $S(q, E)$ where q is along $[0, 2+q, 0]$ direction. This map reveals the dispersion of TA phonons. The corresponding directions are denoted in **b** in H0L reciprocal space plane where $S(\mathbf{q})$ was obtained by integrating $S(\mathbf{q}, E)$ in energy in a range from 0 to 0.05 meV. (**d**) $S(q)$ along $[0, 2+q, 0]$ (horizontal) and $[0+q, 2, 0]$ (vertical) direction with E integrated from 0 to 0.05 meV. Intensities are shown in log scale. (**c**) Demonstration of zone centre LA and TA phonon dispersion. q_{TA} and q_{LA} are wavevectors of TA and LA phonons at E_{min}. q_{TA} is always higher than q_{LA}.

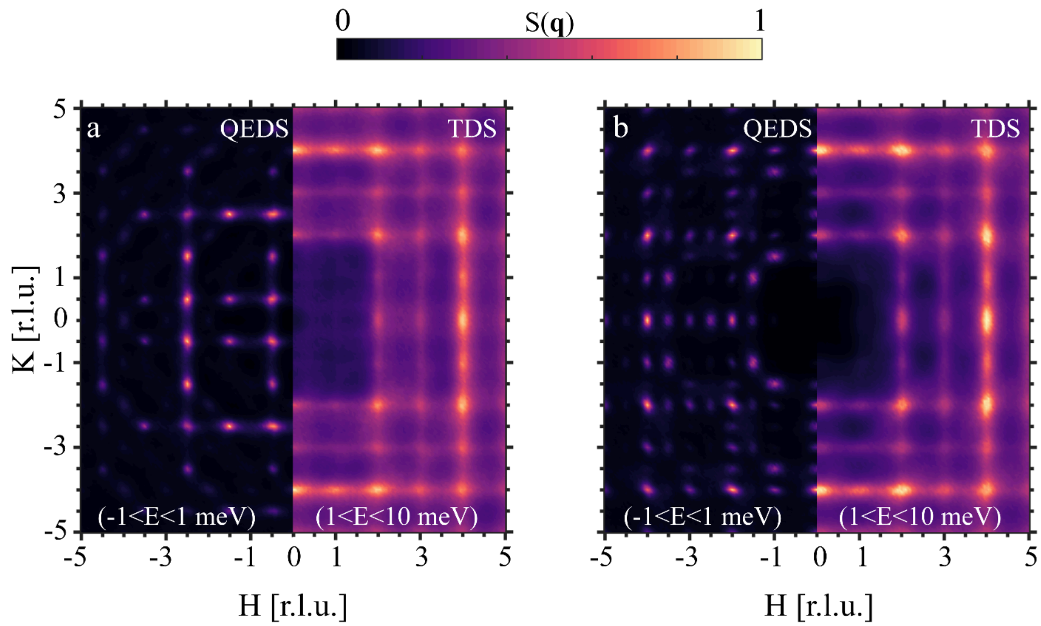

Supplementary Figure 18: **QEDS and TDS components obtained by integrating MD-derived $S(\mathbf{q}, E)$ in the HK1.5 plane over $0 < E < 1$ meV and $1 < E < 10$ meV, respectively.** (**a**), $MAPbBr_3$; (**b**), $FAPbBr_3$.

c. Effect of different A-sites on dynamic structure

The varying shapes of MA and FA molecules result in distinct roles in octahedral tilting and their spatial correlations. Here we quantitatively investigate the orientation of the molecules within the octahedral cavities and explore the reasons behind the divergent behaviors of the two systems. At 300K, the polarization vectors of FA molecules consistently point towards the (100) directions, with a restricted angular spread and their rotational degrees of freedom are similarly constrained as shown in Fig. 1 **g** in the main text. In contrast, MA molecules exhibit a broader range of allowed orientations, with only the (100) and (111) directions being prohibited as shown in Fig. 1 **h** in the main text. Furthermore, the pair distribution function of the hydrogen-bromine pairs obtained from MD simulations indicates stronger H-bonding in the local environment of FA molecules compared to MA (Supplementary Figure 19). Consequently, the stronger bonding between the A-sites and the octahedral framework in the FA system explains the longer correlation length of octahedral tilting, as FA plays a more significant role in conducting the tilting along the normal direction.

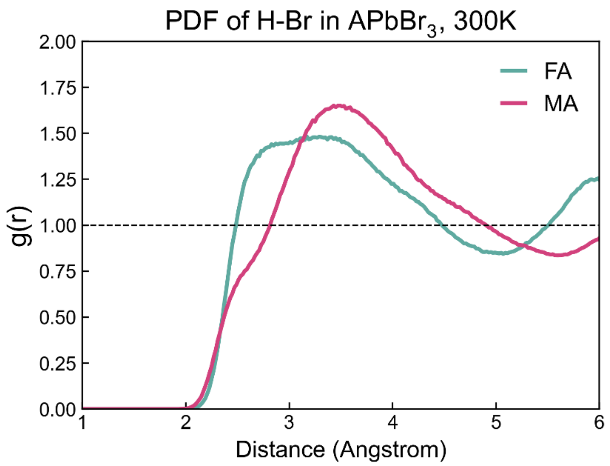

Supplementary Figure 19: **Pair distribution function of H-Br computed from MD trajectories.**

7. PROCEDURE TO SEPARATE TDS AND QEDS IN THE EXPERIMENTAL DATA AND THE PHYSICAL INTERPRETATION OF THEIR RELATIVE INTENSITIES

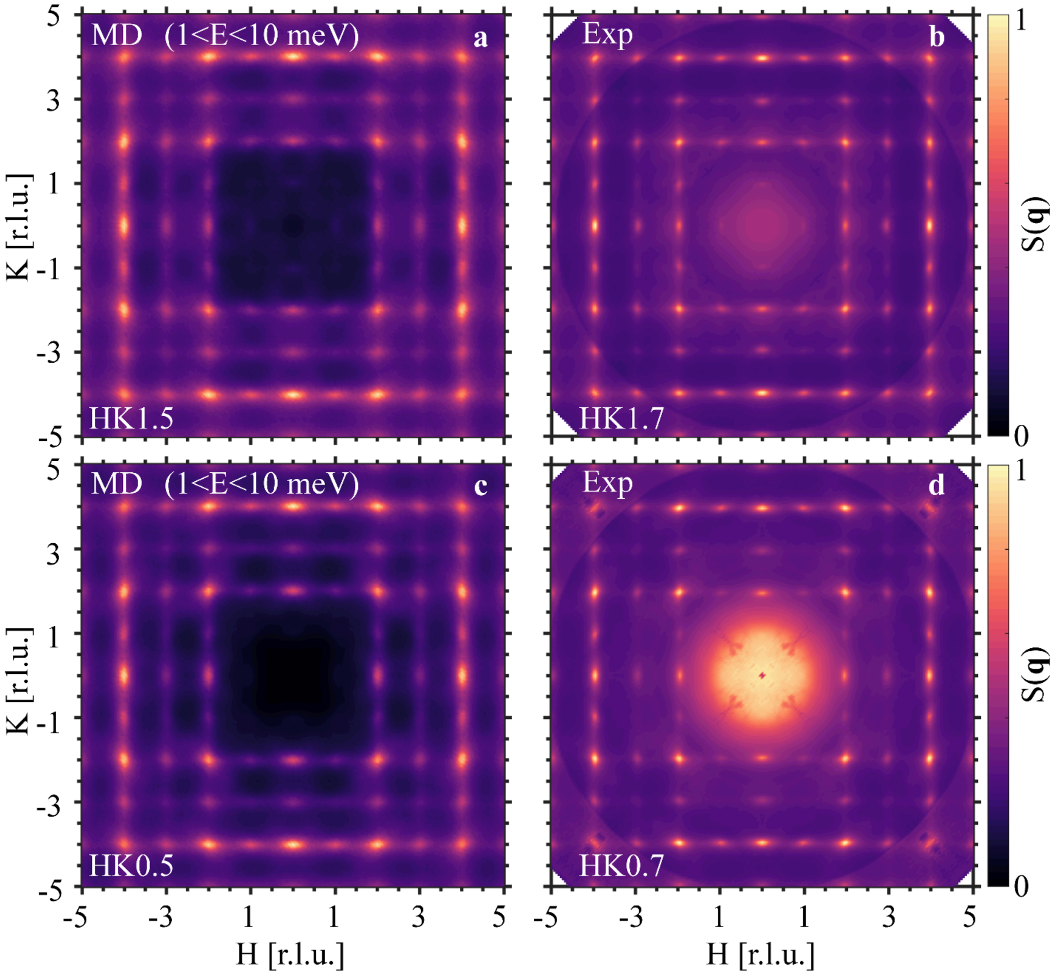

Supplementary Figure 20: **Comparison of MD simulated and experimental TDS in $FAPbBr_3$ at 300 K.** (**a**) and (**c**), show MD derived HK1.5 and HK0.5 $S(\mathbf{q})$ in $FAPbBr_3$ at 300 K obtained by integrating $S(\mathbf{q}, E)$ in energy range from 1 to 10 meV. (**b**) and (**d**), show experimental HK1.7 and HK0.7 $S(\mathbf{q})$ in $FAPbBr_3$ at 300 K.

In our analysis, we distinguish the quasi-elastic diffuse scattering (QEDS) component within the HKn plane of reciprocal space by subtracting the total diffuse scattering observed at HKn from the values measured at HKn + 0.2, where n is a half-integer. This approach assumes that the HKn + 0.2 plane is predominantly composed of Thermal Diffuse Scattering (TDS) signals. Utilising the $S(\mathbf{q}, E)$ derived from molecular dynamics (MD) trajectories, we validate this assumption for the materials under investigation. Supplementary Figure 20(**a**) and (**c**) present the MD-derived $S(\mathbf{q})$ for $FAPbBr_3$ at 300 K at HK1.5 and HK0.5, respectively, obtained by integrating $S(\mathbf{q}, E)$ over the energy range of 1 to 10 meV. This integration captures the TDS signal, attributed dominantly to acoustic phonons, while effectively isolating the QEDS components. The resemblance of these MD-derived diffuse scattering patterns to the experimentally collected patterns at HK1.7 and HK0.7, as shown in Supplementary Figure 20(**b**) and (**d**), confirms that the experimental HKn + 0.2 planes predominantly contain TDS. Thus, our subtraction method for isolating QEDS components is substantiated. TDS manifests across every two-dimensional cross-section of reciprocal space. In contrast, the local structural phenomena contributing to QEDS manifest predominantly in half-integer planes. Consequently, while a plane such as HK1.5 contains both QEDS and TDS, a plane like HK1.7, being sufficiently distant from HK1.5, is expected to encompass only TDS. We provide a further rationale for confidently attributing the scattering intensity at HKn + 0.2 specifically to TDS. In Supplementary Figure 21, we present comparative data of total diffuse scattering in the HK1.5 and HK1.7 planes at 300 K and 200 K. The QEDS signal observed at 300 K (Supplementary Figure 21 **a**) converges to Bragg peak scattering at 200 K (Supplementary Figure 21 **b**), while the

TDS patterns remain consistent through the phase transition as shown in (Supplementary Figure 21 **c** and **d**). The same TDS pattern is also evident in the HK1.5 plane at 200 K.

At lower temperatures, a reduction in TDS intensity is anticipated due to the diminishing phonon population, as evident in Supplementary Figure 21 **c** and **d**. Furthermore, the 1D cross-section at K = 2 for these two temperatures, shown in Supplementary Figure 21 **e**, corroborates this observation. Additionally, the quadratic dependence of $S(\mathbf{q})$ on $\mathbf{q}$ for harmonic phonons, characteristic of TDS, is evident in Supplementary Figure 21 **f**. Here, the cross-section at K = 2 in different 2D planes, separated by 0.2 in the L direction of reciprocal space, shows a variance in intensity, with a higher intensity at the elevated $\mathbf{q}$ value plane, aligning with the expected behaviour of TDS.

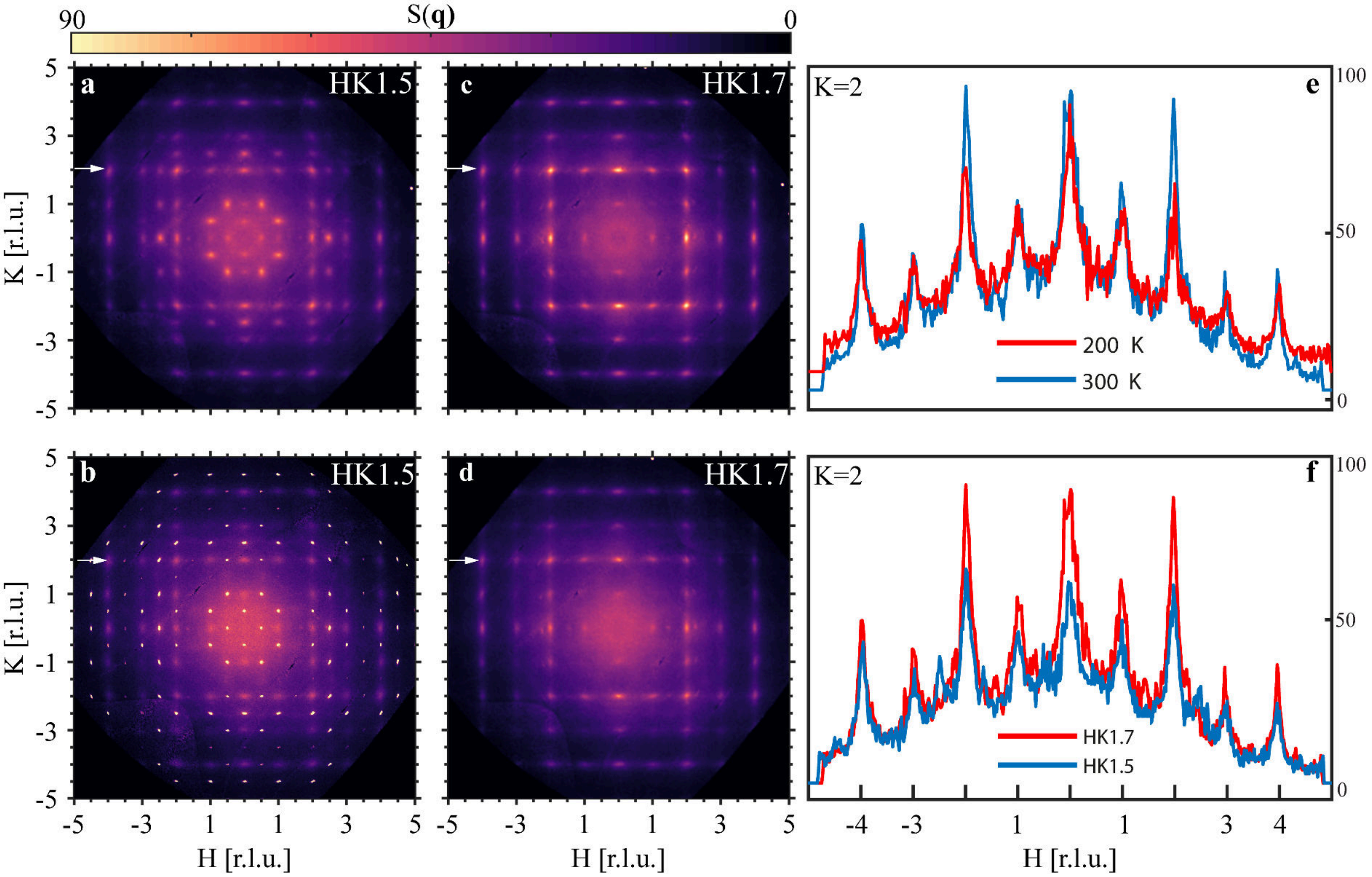

Supplementary Figure 21: **The observation of TDS in $\mathbf{FAPbBr_3}$.** (**a**) and (**b**) show $S(\mathbf{q})$ of HK1.5 reciprocal space planes at 300 and 200 K, respectively. (**c**) and (**d**), show $S(\mathbf{q})$ of HK1.7 reciprocal space planes at 300 and 200 K, respectively. (**e**) 1D cross sections at $K = 2$ of $HK1.7$ planes at 300 and 200 K. (**f**) 1D cross sections at $K = 2$ of $HK1.5$ $HK1.7$ planes at 300 K. The data is obtained using synchrotron radiation at MX1 at Australian Synchrotron.

In the investigation of diffuse scattering contributions, Inelastic Neutron Scattering (INS) plays a crucial role in classifying TDS and QEDS. INS enables the measurement of excitations' energies associated with diffuse scattering. Particularly, QEDS is attributed to near-static excitations, detectable in the elastic regime centered at zero energy transfer. In Supplementary Note 7 **a** and **b** we mark the directions in the reciprocal space indicated by white rectangles, in which we performed line scans using triple axis spectrometer, in both elastic and inelastic regime. With single crystal X-ray diffraction employed to generate these $S(\mathbf{q}, E)$ maps we are unable to differentiate elastic from inelastic scattering and as a result, both TDS and QEDS signals will be captured. However, INS selectively filters QEDS, as evidenced in Supplementary Note 7 **c** and **d** where the observed peaks at zero energy transfer correspond to QEDS identified in the X-ray 2D $S(\mathbf{q})$ maps. Supplementary Note 7 **e** and **f** demonstrate that X-ray scattering captures both TDS and QEDS peaks, corroborating this classification approach.

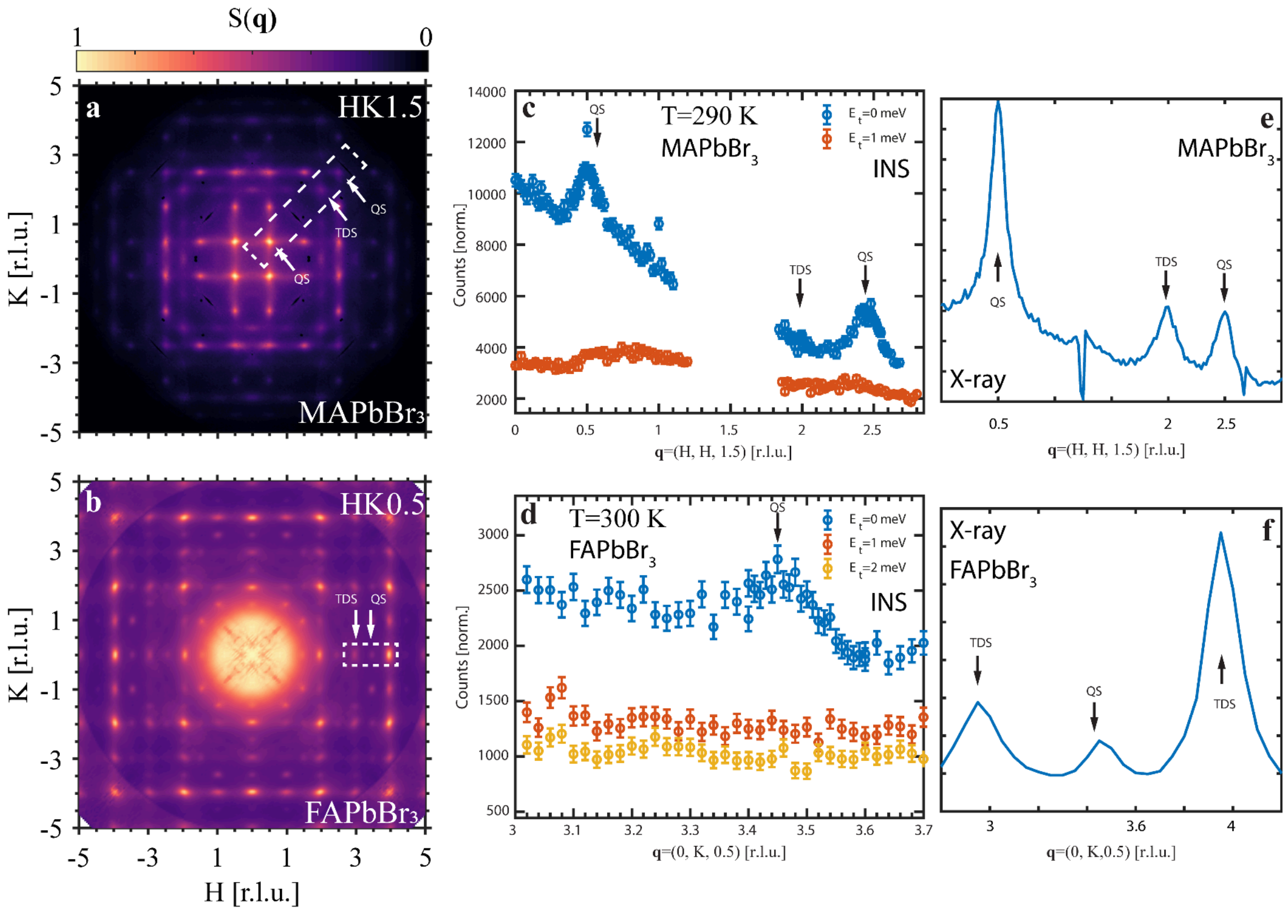

Supplementary Figure 22: **Comparison of TDS and QEDS from INS and X-ray scattering.** (**a**) shows $S(\mathbf{q})$ of HK1.5 $MAPbBr_3$ reciprocal space plane at 300. (**b**) shows $S(\mathbf{q})$ of HK0.5 $FAPbBr_3$ reciprocal space plane at 300. The regions which were scanned with INS are denoted with a white rectangle. Arrows show TDS and QEDS peaks. (**c**) Elastic ($E_t = 0\,\text{meV}$) and inelastic ($E_t = 1\,\text{meV}$) q scans across the $[-0.5, -0.5, L]$ direction of $MAPbBr_3$ at ($T = 290\,\text{K}$). (**d**) Elastic ($E_t = 0\,\text{meV}$) and inelastic ($E_t = 1\,\text{meV}$, $E_t = 2\,\text{meV}$) q scans across the $[0, \text{K}, 0.5]$ direction of $FAPbBr_3$ at ($T = 300\,\text{K}$). (**e**) The same 1D scan as in (**c**) but extracted from 2D X-ray $S(\mathbf{q})$ in (**a**). (**f**) The same 1D scan as in (**e**) but extracted from 2D X-ray $S(\mathbf{q})$ in (**b**). Data in (**c**) and (**d**) is presented as $\bar{n} \pm \sqrt{n}$, where $\bar{n}$ denotes the mean and $\sqrt{n}$ (depicted with error bars) represents the Poisson counting error for n detected neutrons.

In the main text, we claim that the probability of finding local dynamic nanodomains in $FAPbBr_3$ is lower than in $MAPbBr_3$. This claim is grounded in the observation that QEDS signals are significantly weaker in $FAPbBr_3$ than in $MAPbBr_3$. The contrasting values in TDS and QEDS intensity are evident when comparing the $S(\mathbf{q})$ between $MAPbBr_3$ and $FAPbBr_3$ in the top quadrants of Main Text Fig.1 **a** and **b**, where in both materials X point scattering approximates the TDS signal and R and M point scattering represent the QEDS signals in $MAPbBr_3$ and $FAPbBr_3$, respectively. The R diffuse peaks are significantly stronger than the X peaks in $MAPbBr_3$, whereas in $FAPbBr_3$, their intensities are comparable. Thus, the intensity of the QEDS signal can serve as a measure of disorder and dynamic local symmetry breaking in halide perovskite materials.

To facilitate a direct comparison of TDS and QEDS intensities between these two compounds, we have integrated $S(\mathbf{q})$ in a narrow $\mathbf{q}$ range originating in three distinct regions in reciprocal space each of which is expected to be dominated by either TDS, QEDS or Bragg scattering. We denote the integrated intensity with S. This integration has been performed for several representative peaks, and the results are detailed in Supplementary Table IV and Supplementary Table V. In our analysis, we introduced a column named "Ratio" in which we divide the obtained scattering function S by the average integrated intensity of representative Bragg peaks. This approach allows for a more equitable comparison between $MAPbBr_3$ and $FAPbBr_3$, considering potential variations in their structure factors and the total volumes of the crystals we measured. From the data presented in the two tables, we observe distinct trends in the relative strengths of QEDS and TDS for each compound. Specifically, in $MAPbBr_3$, QEDS

TABLE IV: Quantitative comparison between TDS and QEDS intensities in $MAPbBr_3$ at 300 K.

Plane	K and L	Type	S	Ratio
1.5KL	2.5 1.5	QEDS	200e3	8e-3
1.5KL	2 1	TDS	100e3	4e-3
1.7KL	2 1	TDS	200e3	4e-3
0KL	2 1	Bragg	2.5e7	

TABLE V: Quantitative comparison between TDS and QEDS intensities in $FAPbBr_3$ at 300 K.

Plane	K and L	Type	S	Ratio
HK1.5	2.5 1	QEDS	50e3	1.7e-3
HK1.5	2 2	TDS	70e3	2.3e-3
HK1.7	2 2	TDS	100e3	3.3e-3
HK0	2 2	Bragg	3e7	

appears to be approximately twice as strong as TDS. In contrast, for $FAPbBr_3$, TDS is about 1.35 times stronger than QEDS. QEDS intensity in $MAPbBr_3$ is around 4 times stronger than in $FAPbBr_3$, aligning with our hypothesis that the density of dynamic nanodomains in $MAPbBr_3$ is higher than in $FAPbBr_3$. The same analysis has been employed for a number of different peaks where the same trend was observed.

8. THE QUANTITATIVE ANALYSIS OF SPATIAL CORRELATIONS FROM MD DATA

Through a quantitative analysis of real-space MD trajectories, we have estimated the volumetric density of dynamic nanodomains within the simulated supercell. As depicted in Supplementary Figure 23 **a**, the distribution of tilt angles for $MAPbBr_3$ and $FAPbBr_3$ is collected over a specified period of 1 ns. Our empirical evaluation identifies tilt angles below 5 degrees as part of the octahedra's random tilt background, while angles above this threshold are indicative of dynamic local nanodomains exhibiting lower symmetry. By integrating the data presented in the histogram of Supplementary Figure 23 **a**, we calculate the volumetric density of octahedra up to a specific tilt angle threshold, represented on the x-axis in Supplementary Figure 23 **b**. This analysis reveals a consistently higher density of tilted octahedra in $MAPbBr_3$ compared to $FAPbBr_3$, across any chosen threshold. Employing a 5-degree threshold, we find that 19.1% of octahedra are tilted beyond this angle in $FAPbBr_3$ and 32.9% in $MAPbBr_3$. Consequently, the volumetric density of dynamic nanodomains is approximately 1.7 times greater in $MAPbBr_3$ than in $FAPbBr_3$.

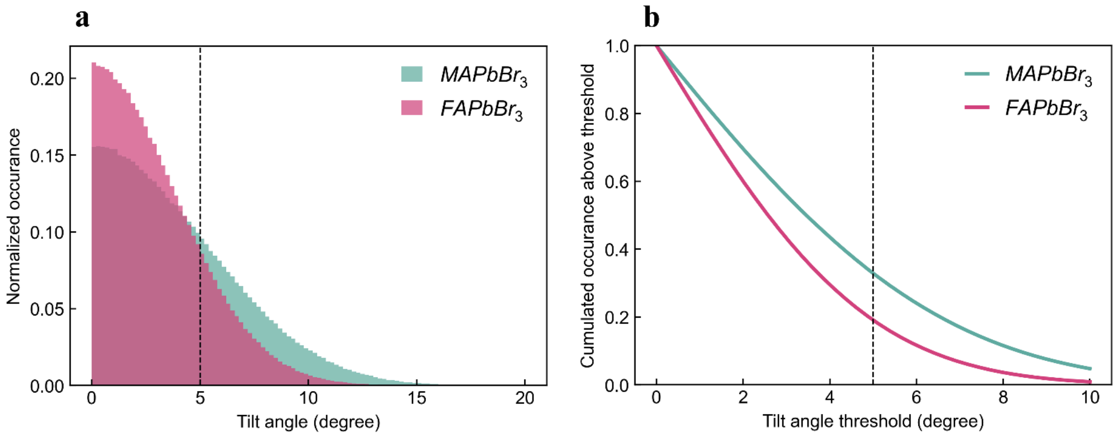

Supplementary Figure 23: **The estimation of volumetric density of dynamic nanodomains from MD real space trajectories. a** Histogram of octahedral tilt distributions in $MAPbBr_3$ and $FAPbBr_3$ at 300 K with vertical line denoting the threshold angle. **b** Fraction of octahedra that exhibit tilting as a function of threshold tilting angle in $MAPbBr_3$ and $FAPbBr_3$ at 300 K.

We further apply quantitative analysis to determine the shape and symmetry of these dynamic nanodomains. From MD trajectories we extract the spatial correlation functions and present them in Supplementary Figure 24.

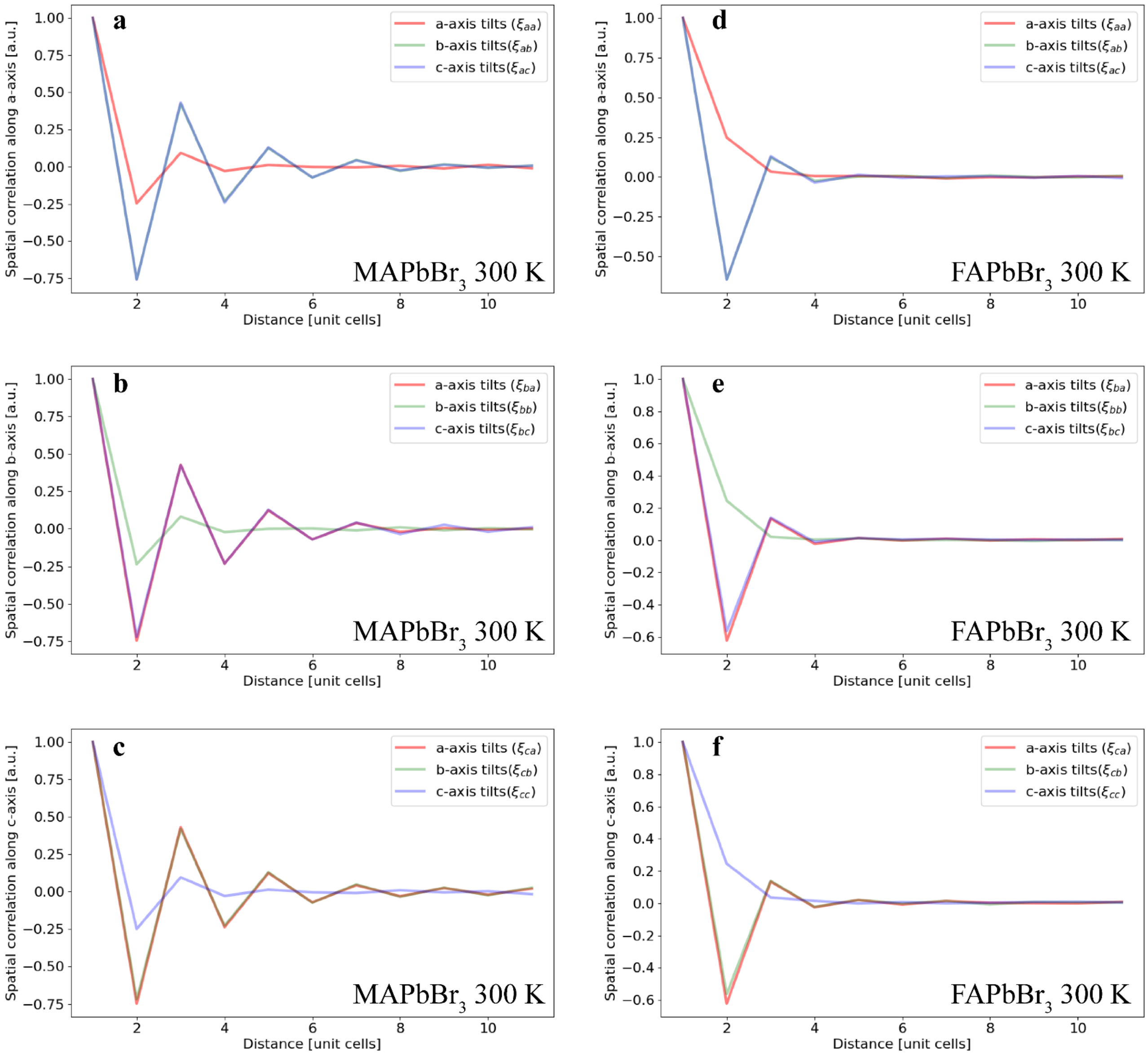

Supplementary Figure 24: **Spatial correlation functions determine symmetry and shape of dynamic nanodomains at 300 K.** (**a**)-(**c**) Spatial correlation functions of three types of octahedral tilts along three crystallographic axes for $MAPbBr_3$ at 300 K derived from MD trajectories. For example, in (**a**) we plot three spatial correlation functions. Each function measures spatial correlations of a certain type of octahedral tilt (either a, b or c-axis tilts) along a crystallographic axis. The corresponding correlation lengths derived from these curves by fitting them to exponentially decaying functions are thus ξ_{aa} ξ_{ab} ξ_{ac}. (**d**)-(**f**) Spatial correlation functions of three types of octahedral tilts along three crystallographic axes for $FAPbBr_3$ at 300 K derived from MD trajectories.

The negative correlation value in the plots corresponds to the case of out-of-phase tilting correlation while positive to the in-phase tilting correlation. From the plots, we observe that in $MAPbBr_3$ spatial correlation along axis n is always the shortest for the axis n tilts, while the other two axis tilts exhibit longer correlation. The correlation values alternate in a sign for each case in $MAPbBr_3$ and thus we can conclude that dynamic nanodomains possess local $I4/mcm$ $(a^0a^0c_S^-)$ symmetry. However, in the case of FAPbBr3, n axis tilt always exhibits a positive correlation while the two-axis tilts exhibit a negative correlation. This corresponds to local $P4/mbm$ symmetry or $(a^0a^0c_S^+)$ in modified Glazer notation. This analysis helps us confirm that the symmetry of the local structure aligns with our experimental results, derived by utilising a phenomenological model.

We fit each spatial correlation function in Supplementary Figure 24 to exponential decay function [26] to derive the spatial correlation length tensor Ξ. This is because three types of octahedral tilts are possible (rotation along a, b or c axis) and we need to track spatial correlations of three tilt types along all three crystallographic axes. The spatial correlation length tensor Ξ derived from spatial correlation functions of $MAPbBr_3$ at 300 K is given below. Each correlation length value is given as a number of unit cells.

$$\Xi = \begin{bmatrix} \xi_{aa} & \xi_{ab} & \xi_{ac} \\ \xi_{ba} & \xi_{bb} & \xi_{bc} \\ \xi_{ca} & \xi_{cb} & \xi_{cc} \end{bmatrix} = \begin{bmatrix} 0.682 & 2.169 & 2.166 \\ 2.116 & 0.683 & 2.123 \\ 2.094 & 2.148 & 0.696 \end{bmatrix} \tag{S11}$$

where: ξ_{aa}, ξ_{bb}, and ξ_{cc} are the diagonal components representing normal correlation length $\xi_\perp$ or the half the thickness of dynamic nanodomain disc, and the off-diagonal elements (ξ_{ab}, ξ_{ac}, etc.) correspond to $\xi_\parallel$, or the radius of the discs. The fact that all diagonal elements have nearly the same value and that also all off-diagonal elements have the same value confirms that these discs are oriented orthogonally relative to each other i.e. form dynamic local twins which we also show in the main text using the phenomenological simulation. Thus to derive average $\xi_\parallel$ and $\xi_\perp$ we average all the off-diagonal and diagonal elements, respectively. Finally, we present a table where we compare correlation length diameters (i.e. two times the MD correlation lengths above) derived from the real space analysis of MD trajectories, single crystal X-ray diffuse scattering.

We note that the cubic-tetragonal phase transition temperature we obtain from MD simulations in $MAPbBr_3$ is approximately 270 K (as shown in Supplementary Figure 25 **a** when values from heating and cooling cycles are averaged) which is around 35 K higher than the experimentally observed phase transition. Thus, to make a fair comparison we compare the correlation lengths from MD trajectories to the 300 K X-ray values both at the same absolute temperature (300 K) and at approximately the same offset from the phase transition temperature (340 K) $MAPbBr_3$, as presented in Supplementary Table VI.

We also observe that the diffuse scattering profiles we simulate from MD trajectories at 340 K in $MAPbBr_3$ match better with experimental observations. This is obvious from Supplementary Figure 26 where 340 K MD simulated $S(\mathbf{q})$ exhibit more pronounced rods along the R-M direction in the reciprocal space compared to 300 K MD simulated $S(\mathbf{q})$. This is in agreement with data presented in Supplementary Table VI where correlation lengths diameters at 340 K MD match better the experimentally derived diameters. Additionally, in Supplementary Figure 27 we show that dynamic nanodomain lifetimes are shorter at 300 K compared to lifetimes at 340 K, indicating that the dynamic nanodomains slow down as the tetragonal phase is approached by cooling.

TABLE VI: Comparison of MD and experimentally derived spatial correlation lengths for $MAPbBr_3$. $\xi_\perp$ and $\xi_\parallel$ are the thickness and diameter, respectively of the disc-shaped dynamic nanodomains. Ratio corresponds to $\frac{\xi_\parallel}{\xi_\perp}$. Note that, in the MD case, we calculate twice the correlation length to represent the diameter, rather than the radius, of the dynamic nanodomains.

$MAPbBr_3$	MD 300K	MD 340K	Exp X-ray 300K
$\xi_\perp$ [Å]	8.5892	5.752	6.264±0.03
$\xi_\parallel$ [Å]	23.86	20.59	20.506±0.1
Ratio	2.78	3.58	3.273

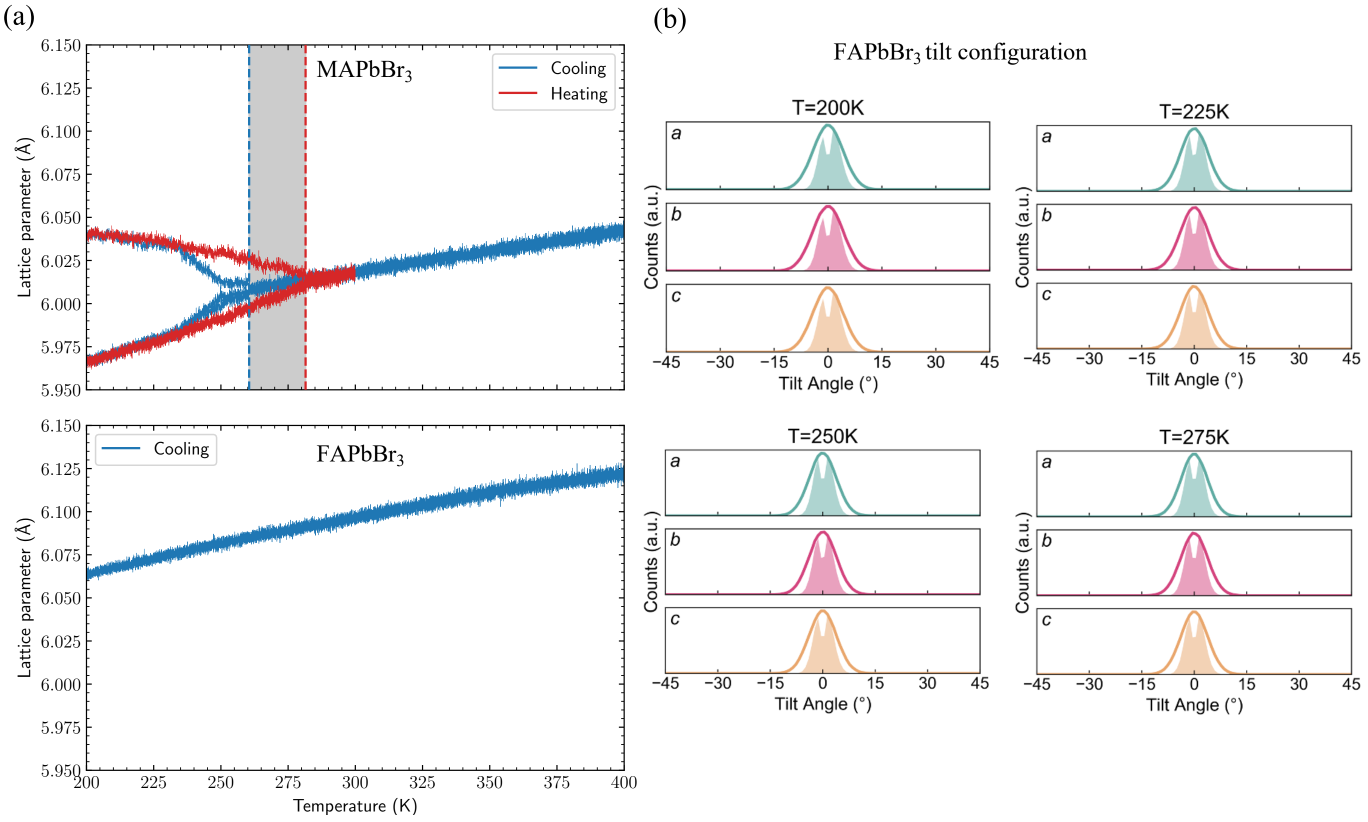

Supplementary Figure 25: **Phase transition temperatures from MD simulations.** (**a**) Lattice parameters in $MAPbBr_3$ and $FAPbBr_3$ during cooling (blue lines) and heating (red lines) NPT MD runs. The cooling/heating rates were 400 K/ns. As a result of the rapid cooling/heating rates, there is substantial hysteresis in the phase transition temperature of $MAPBBr_3$ (grey shaded area). The average of the cooling and heating simulations gives a phase transformation temperature of $\sim$ 270 K. No direct signature of phase transformation is visible from the lattice parameters in the $FAPbBr_3$ case. (**b**) To probe whether the phase transition occurs, we analysed NPT MD trajectories and quantified octahedral tilting in $FAPbBr_3$ at 200 K, 225 K, 250 K and 275 K. These simulations started from configurations at the corresponding temperature from the cooling run in (a), and were then ran for 100 ps. Each panel corresponds to one axis. The solid lines denote the dynamic distribution of tilting. The shaded area below the solid lines is the distribution of the tilt angle correlation with the next nearest neighbour along the same direction (see [26] for details). The corresponding global Glazer tilting pattern is $a^0a^0a^0$ at all temperatures. At 200 K, we observe a clear asymmetry in the tilt correlation towards positive values for all 3 axes, which is potentially a precursor of a transition to $a^+a^+a^+$.

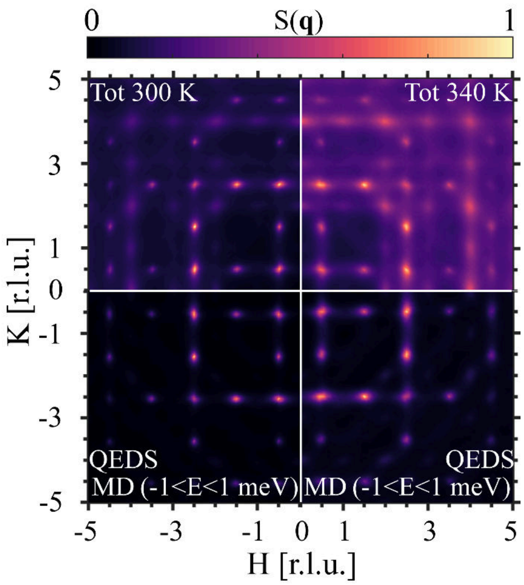

Supplementary Figure 26: **Comparison of 340 K and 300 K MD simulated diffuse scattering in $\mathbf{MAPbBr_3}$.** Left quadrants correspond to total $S(\mathbf{q})$ and QEDS component of $S(\mathbf{q})$ at 300 K, while right quadrants correspond to data simulated at 340 K.

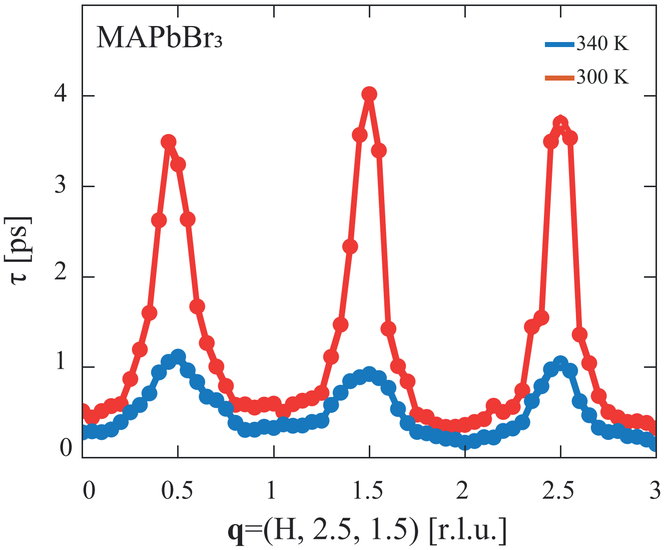

Supplementary Figure 27: **Comparison of dynamic nanodomain lifetimes along R-M diffuse rods at 340 K and 300 K from MD simulated $S(\mathbf{q}, E)$ in $\mathbf{MAPbBr_3}$.**

For $FAPbBr_3$, we observe no clear indication of the phase transition. At 200 K, we do see an asymmetry in the tilt correlation towards positive values for all 3 axes, this may indicate that the system is starting to transform towards the $a^+a^+a^+$. As the experimentally observed transition occurs at 265 K, it is thus likely that the MLFF underestimates the phase transition temperature, which could explain the discrepancy between experimentally observed and MD derived correlation length diameters as shown in Supplementary Table VII. However, observing phase transitions directly from the inherently limited simulation time accessible to MD is often difficult if there is a significant first-order character to the transition. Indeed, in this case, the system can remain in the phase it was initiated in ($a^0a^0a^0$ in this case) for a time that is significantly longer compared to the length of the MD simulation.

Nevertheless, regardless of the MD transition temperature, the ratio of the two correlation length diameters is

comparable with experimentally derived, which is also significantly lower than the ratio in $MAPbBr_3$. Thus, we conclude that $MAPbBr_3$ dynamic nanodomains form discs of pancakes as the ratio of normal correlation to parallel is significant, while in $FAPbBr_3$ nanodomains are more isotropic and exhibit lower ratio values.

TABLE VII: Comparison of MD and experimentally derived spatial correlation lengths for $FAPbBr_3$. $\xi_\perp$ and $\xi_\parallel$ are the thickness and diameter, respectively of the disc-shaped dynamic nanodomains. Ratio corresponds to $\frac{\xi_\parallel}{\xi_\perp}$. Note that in the MD case we compare two times the correlation lengths, to represent the diameter, rather than the radius, of the dynamic nanodomains.

$FAPbBr_3$	MD 300K	Exp X-ray 300K
$\xi_\perp$ [Å]	8.267	14.21±0.58
$\xi_\parallel$ [Å]	15.742	21.48±8.6
Ratio	1.91	1.51

In Supplementary Table VIII and Supplementary Table IX we show a direct comparison between experimental and MD-derived correlation lengths. For calculating MD correlation lengths, apart from using analysis in real space we also fitted MD-derived $S(\mathbf{q})$ to our phenomenological model to extract the correlation lengths from reciprocal space. We still note a slight misalignment between correlation lengths achieved with two different approaches (real space and reciprocal space analysis of MD data). A factor contributing to the discrepancy between real space and reciprocal space analysis of MD-derived correlation length diameters stems from the methodology employed to obtain correlation lengths. In reciprocal space analysis, correlation lengths are determined by fitting the MD-derived $S(\mathbf{q})$ at R or M points in the Brillouin zone to a Lorentzian function, thereby exclusively probing the correlation lengths of purely in-phase and out-of-phase tilted local nanodomains. Conversely, when analysing octahedral correlations in real space from MD data, we inherently include contributions from all possible tilt correlations, not just the in- and out-of-phase ones. As a result, the correlation lengths obtained using these two methodologies might differ, with correlation lengths derived from MD reciprocal space analysis expected to provide a more direct comparison with experimental values.

TABLE VIII: Comparison of correlation lengths obtained from MD simulations and experiments for $MAPbBr_3$. MD 340K rec space column shows correlation lengths and correlation lengths ratio(R) obtained by fitting MD $S(q)$ with the phenomenological model. MD 340K real space column shows correlation lengths and correlation lengths ratio (R) obtained by real space analysis of MD trajectories.

**Correlation length\Approach**	**MD 340K Rec space**	**MD 340K Real space**	**Exp 300K**
$\xi_\parallel$ [$\AA$]	20.539 ± 0.047	20.59 ± 0.331	20.506 ± 0.121
$\xi_\perp$ [$\AA$]	7.621 ± 0.019	5.752 ± 0.522	6.264 ± 0.035
R	2.696 ± 0.009	3.58 ± 0.33	3.273 ± 0.027
RMS [%]	2.152	/	5.072

TABLE IX: Comparison of correlation lengths obtained from MD simulations and experiments for $FAPbBr_3$. MD 250K rec space column shows correlation lengths and correlation lengths ratio (R) obtained by fitting MD $S(q)$ with the phenomenological model. MD 250K real space column shows correlation lengths and correlation lengths ratio (R) obtained by real space analysis of MD trajectories.

**Correlation length\Approach**	**MD 250K Rec space**	**MD 250K Real space**	**Exp 300K**
$\xi_\parallel$ [$\AA$]	20.6775 ± 0.5232	18.244 ± 0.3986	21.48362 ± 0.21476
$\xi_\perp$ [$\AA$]	14.5974 ± 0.3693	10.58 ± 0.189	14.21042 ± 0.14202
R	1.41652 ± 0.05069	1.724 ± 0.0486	1.51182 ± 0.02137
RMS [%]	12.2452	/	5.7171

9. VERIFICATION OF THE ALLEGRO MACHINE LEARNED FORCE FIELDS

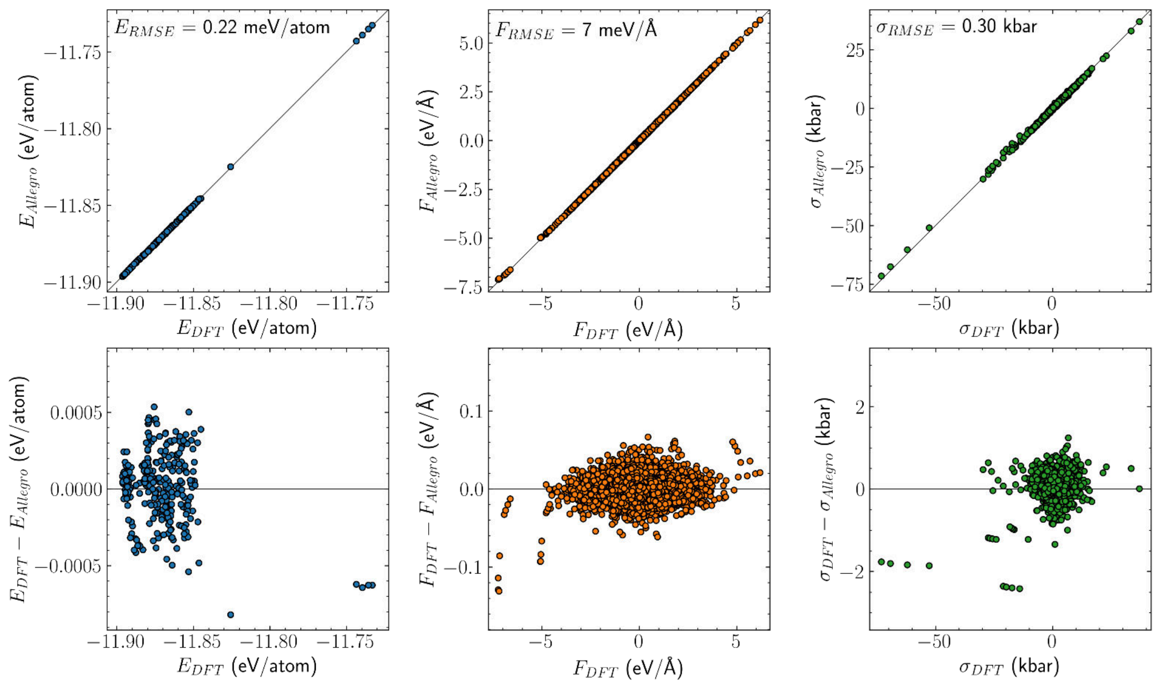

Supplementary Figure 28: Parity plot (top row) and error distribution (bottom row) for the $MAPbBr_3$ Allegro MLFF. Root mean squared errors (RMSEs) of energies, force components and stress components are given in the top row.

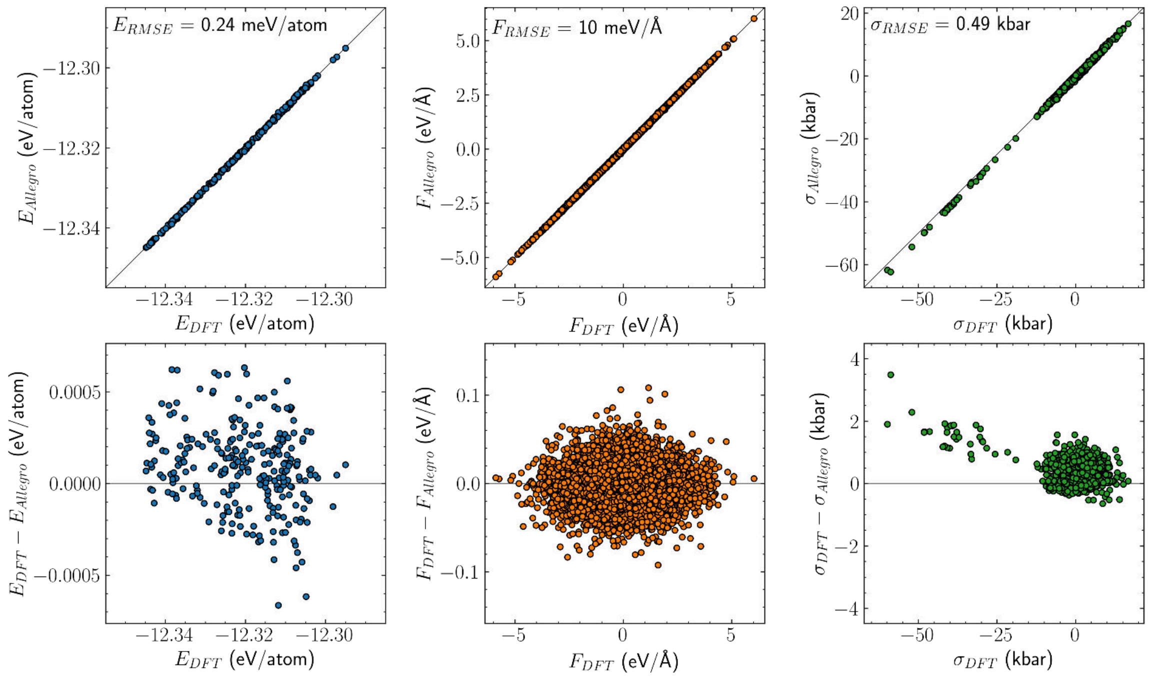

Supplementary Figure 29: Parity plot (top row) and error distribution (bottom row) for the $FAPbBr_3$ Allegro MLFF. Root mean squared errors (RMSEs) of energies, force components and stress components are given in the top row.

10. ANALYSIS OF ELECTRONIC STRUCTURE FROM MD DATA

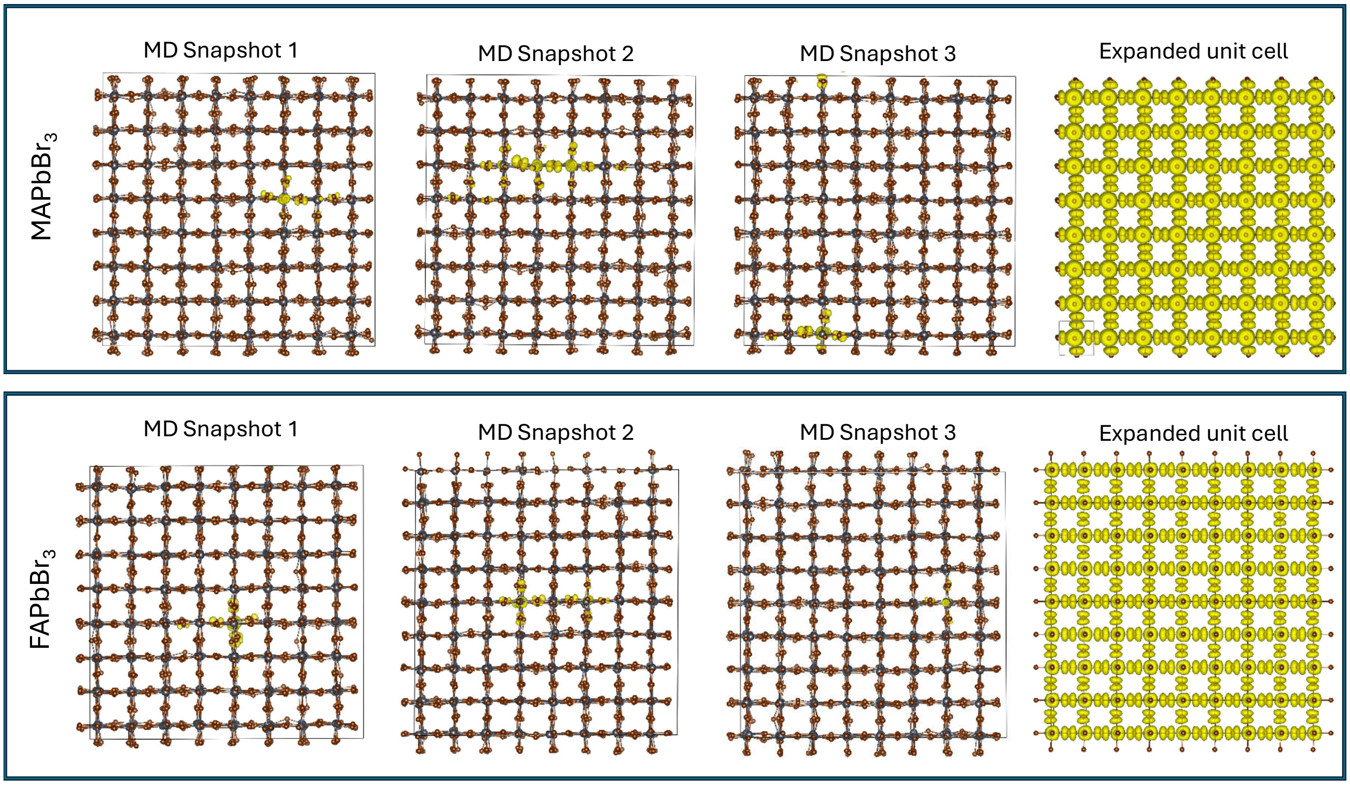

Supplementary Figure 30: Isosurfaces of DFT calculated partial charge density of the valence band maximum (VBM) in $MAPbBr_3$ (top row) and $FAPbBr_3$ (bottom row) for snapshots extracted from MD runs at 300 K with our Allegro MLIP at 300K in 8×8×8 supercells. The isosurface value is 10^{-3} $e/\text{Å}^3$ in all cases . For reference, the rightmost column shows the same (10^{-3} $e/\text{Å}^3$) isosurface of the VBM partial charge density calculated in a 1×1×1 pseudo-cubic cell, which is then expanded to match the size of the MD snapshots.

To analyse the impact of dynamics on the electronic structure in $MAPbBr_3$ and $FAPbBr_3$ we have performed DFT calculations of the partial charge density of the valence band maximum (VBM) in $MAPbBr_3$ (top row) and $FAPbBr_3$ (bottom row) for snapshots extracted from MD runs at 300 K with our Allegro MLIP at 300K in 8×8×8 supercells. To afford DFT calculations in these large supercells containing 6144 atoms, we use lower precision DFT settings than in other parts of this work. Specifically, we use the PBEsol exchange correlation functional, a 300 eV energy cutoff, energy threshold for electronic convergence was set to 10^{-4} eV and `PREC` = Normal.

It is evident from Fig. 30 that the charge density associated with the VBM is localized, different from the delocalized states found in the static unit cell. This echoes previous findings that the highly anharmonic vibrations in these materials have a strong impact on their electronic structure.

11. ARGUMENTS IN FAVOUR OF THE DYNAMIC NATURE OF NANODOMAINS

Due to incoherent scattering that is dominated by H atoms in the A-site cations of $MAPbBr_3$ and $FAPbBr_3$ we were unable to experimentally determine the lifetime of nanodomains from quasi-elastic neutron spectroscopy.

We note that properties of diffuse scattering exhibit strong temperature modulation:

- The correlation lengths of these nanodomains increase as the temperature decreases, supported by direct experimental data from X-ray diffuse scattering and inelastic neutron scattering.
- The volume fraction of the domains expands with temperature, demonstrated by the growth of the diffuse scattering peaks.
- The most significant modulation occurs near phase transitions, specifically the cubic-cubic phase transition in $FAPbBr_3$ and the tetragonal-orthorhombic phase transition in $MAPbBr_3$, where the dynamic domains vanish abruptly after the transition.

Although the observed temperature modulation of the diffuse scattering, such as increasing correlation lengths and a growth in the diffuse scattering intensity, suggests an evolving short-range order, these changes alone do not uniquely indicate a dynamic origin. In fact, static short-range order can also exhibit increasing correlation lengths and expanding volume fractions as the temperature decreases. Definitive evidence requires direct, systematic measurements of both the correlation length and the scattering intensity as functions of temperature, along with temporal resolution of the fluctuations.

Dynamic nanodomains have been reported in similar systems such as oxide perovskites and silicates (e.g., quartz and cristobalite). In displacive phase transitions, the softening of anharmonic acoustic phonon modes, associated with rigid octahedral tilting, leads to nonlinear phonon effects, often linked to critical scattering phenomena [27]. There have been a few reports that indicate the origin of diffuse scattering is dynamic [28, 29], all of which also relied on MD simulations to corroborate that finding. We have reported a striking agreement between experimentally observed total diffuse scattering patterns and MD simulations (Supplementary Figure 13). This includes both thermal diffuse scattering and quasi-elastic diffuse scattering features. Correlation lengths have been extracted from MD simulations in both real and reciprocal space (see Supplementary Note 8), all of which show excellent agreement with experimental values (see Supplementary Table VIII and Supplementary Table IX). If the correlation lengths exhibit such strong agreement and the diffuse scattering patterns align so well with experimental results, it would be highly coincidental for MD simulations to predict their dynamic nature with extremely low temporal accuracy while achieving such remarkable spatial accuracy. Therefore, we conclude that the striking correspondence between experimental observations and MD simulations, where MD simulations consistently predict the dynamic nature of these domains, serves as strong evidence that the nanodomains are indeed dynamic.

Furthermore, from the reported high resolution scanning TEM studies on thin films in the literature static disorder giving rise to the diffuse scattering we observe is not readily present, making it especially unlikely to occur in the single crystals we study [30, 31]. It is important to note that in 'image mode' in the TEM these studies will in effect be blind to the dynamic disorder (visible via diffraction) and only sensitive to static disorder as TEM images only probe the time averaged structure [32, 33]. Thus the absence of any diffuse scattering after Fast Fourier Transforming TEM images in high resolution mode indicates the absence of static disorder.

These collective findings strongly suggest the nanodomains are dynamic. We acknowledge we were not able to precisely determine their lifetimes experimentally and can only suggest the values based on MD simulations, which quantitatively agree with experimental results (correlation lengths (Supplementary Note 8) and $S(\mathbf{q})$ (Supplementary Figure 13)).

12. THE ASSIGNMENT OF LOWER TEMPERATURE PHASE OF FAPBBR$_3$

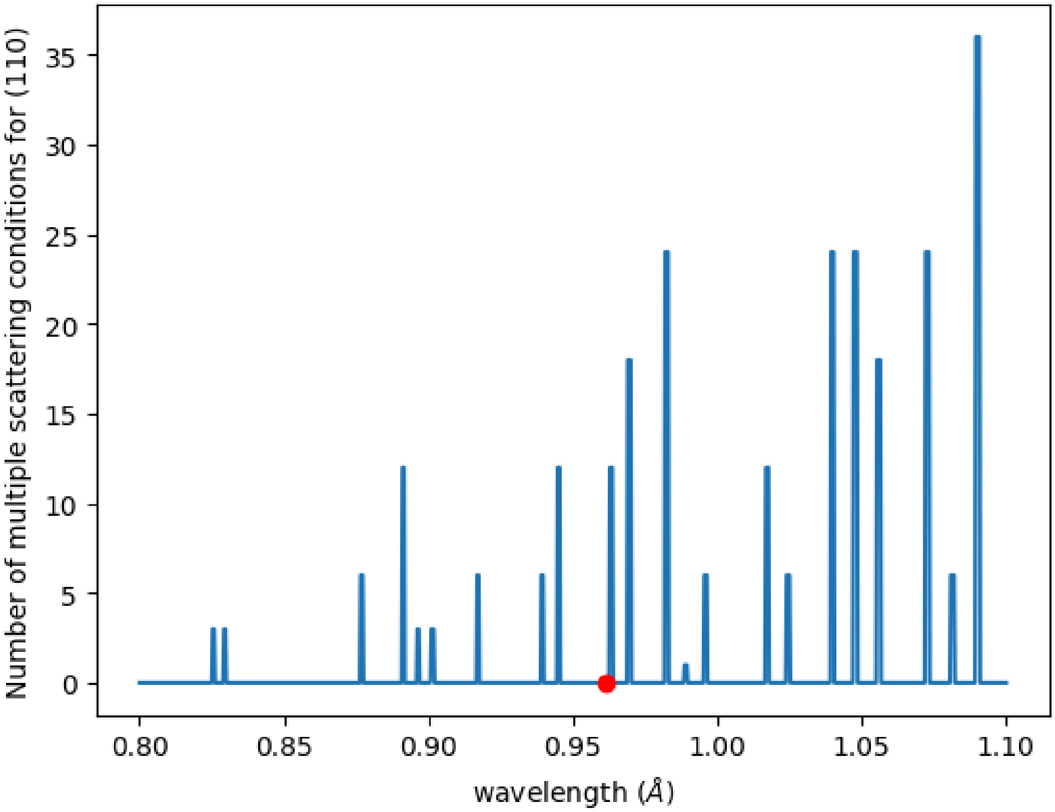

Supplementary Figure 31: **Number of multiple scattering events for (110) reflection of the $Im\bar{3}$ FAPbBr$_3$ supercell as a function of incident X-ray wavelength.** The red dot marks the position of 0.961121 Å.

Although the Bragg pattern produced by assuming three tetragonal $P4/mbm$ macroscopic twin components is almost identical to the Bragg pattern produced with one $Im\bar{3}$ component, the appearance of additional superstructure peaks that only exist in $Im\bar{3}$ and not in twinned $P4/mbm$ confirms the absence of non-merohedral twinning in this material (e.g., the (1.5 1.5 0) reflection referenced to the cubic cell). This also rationalizes the origin of a metrically cubic unit cell measured in high-resolution powder synchrotron X-ray and neutron diffraction experiments at the same temperature [13].

An alternative hypothesis for the origin of these superstructure peaks could be that of multiple scattering, especially given the heavy elements in FAPbBr$_3$. Multiple elastic scattering can occur when a reflected wave matches any other Bragg conditions (e.g., a sphere of radius $|\mathbf{k}_i|$ centered at $\mathbf{k}_i$ passes through reciprocal lattice points other than the origin). We calculated the possibility of matching such a condition for (hkl) reflections in the range, $-8 \leq h, k, l \leq 8$ with respect to the enlarged cubic supercell (a = 11.9171 Å). With the tolerances given by either the best (0.0094 Å^{-1}) or worst (0.0367 Å^{-1}) measured FWHM of the Bragg peaks and the experimental wavelength (0.961121 Å, no multiple scattering conditions were found for the observed (110) (as shown in Supplementary Figure 31) or (330) reflections of the $Im\bar{3}$ supercell. This analysis is equivalent to searching for half-integer reflections of the primitive, parent $Pm\bar{3}m$ unit cell.

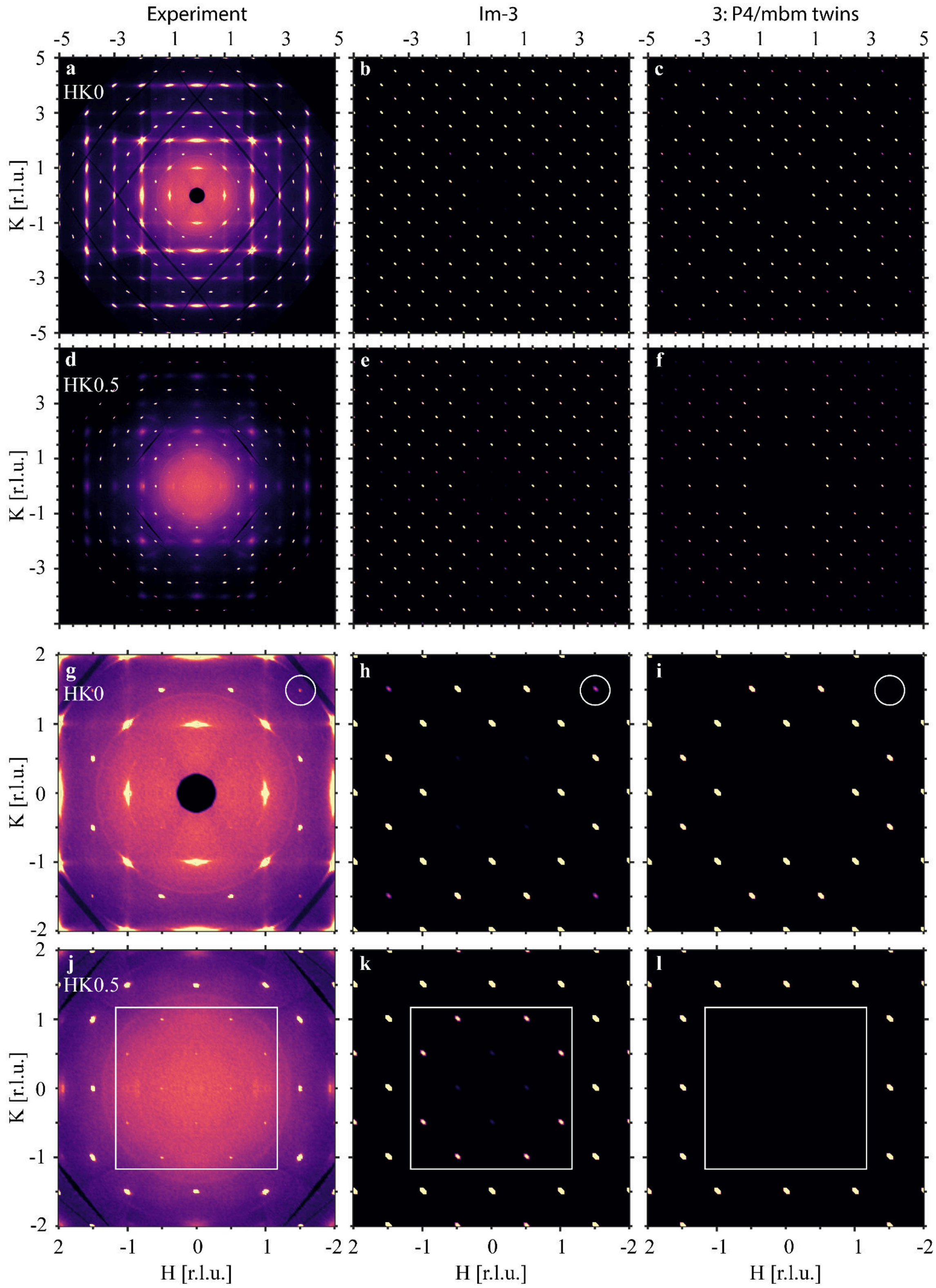

Supplementary Figure 32: **Accurate assignment of low symmetry phase in $FAPbBr_3$ at $T = 200K$.** In the first column 2D experimental diffraction patterns are shown. The second column shows the simulated patterns utilising $Im\bar{3}$ structure we obtained by refining the experimental data, while the third column shows the simulated patterns utilising the structure factors (and incorporating three twins) from $P4/mbm$ cif file which we obtained by refining data. $HK0$ reciprocal space planes are shown in (**a**)-(**c**) and (**g**)-(**i**) and HK0.5 reciprocal space planes are shown in (**d**)-(**f**) and (**j**)-(**l**). The positions of superstructure peaks expected in $Im\bar{3}$ space group are marked with white circles and rectangles.

13. THE INFLUENCE OF BEAM DAMAGE ON LOCAL STRUCTURE

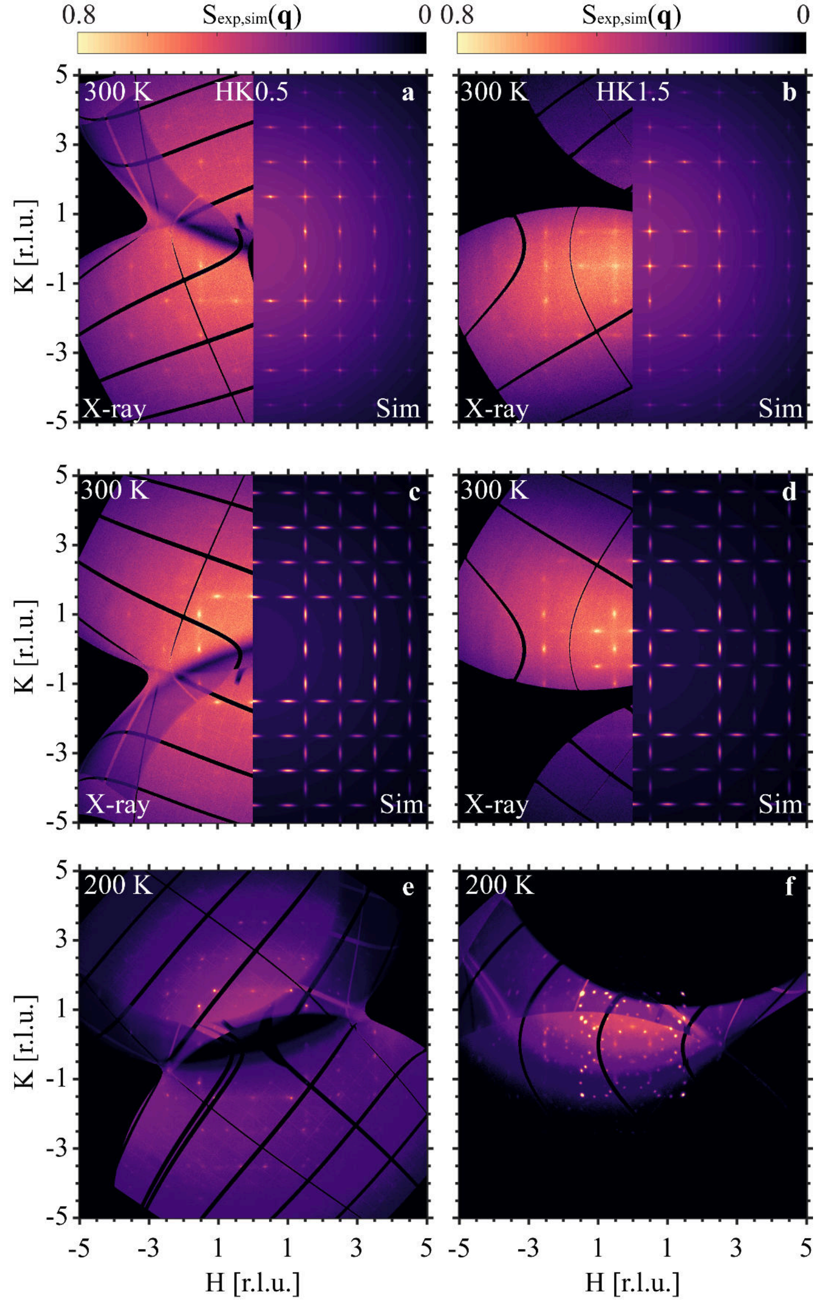

Supplementary Figure 33: **The effect of beam damage on experimental scattering function $S(\mathbf{q})$ in MAPbBr$_3$.** (**a**) and (**b**) represent 2D X-ray experimental scattering function (left panels) and simulated for local $I4/mcm$ structure (right panels). This data was collected at 18 keV within 35 min at X-ray flux of $1.542 \times 10^{13}\,\mathrm{s}^{-1}$ incident on an area of 100 µm x 100 µm. Repeating the experiment after the first exposure under the same conditions, we observe the change in diffuse scattering signals (**c** and **d**, left panels) which we assign to the formation of local $P4/mbm$ structure (as simulated in right panels). We repeated the experiment again but this time at $T = 200\,\mathrm{K}$, and we observed the appearance of additional intensities in the X-ray scattering function (**e** and **f**), which we assign to another beam damage mechanism. We confirm that the sample did not transition to average $I4/mcm$ phase as expected at $T = 200\,\mathrm{K}$.

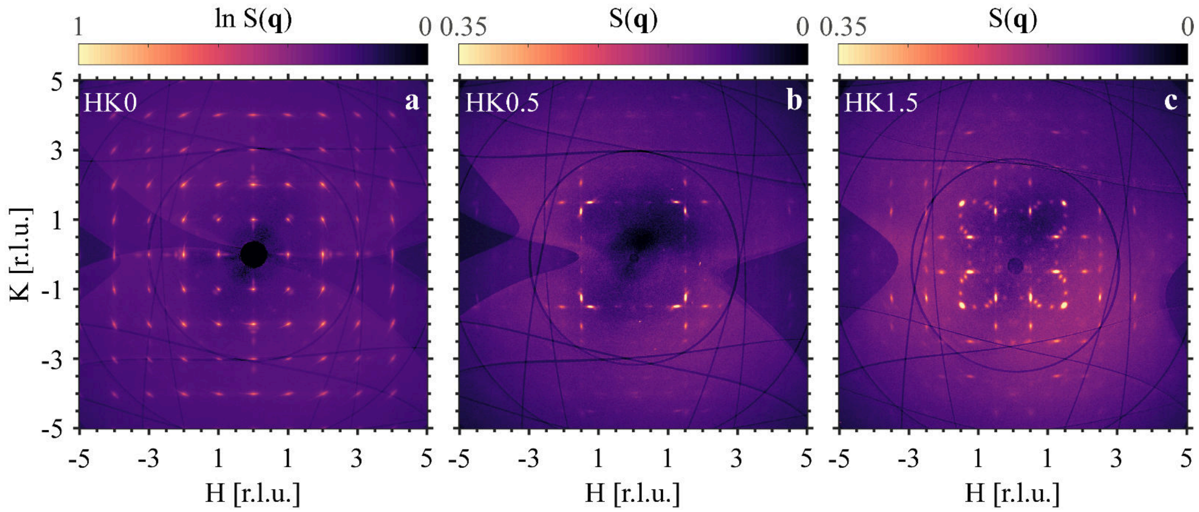

Supplementary Figure 34: **The effect of beam damage on experimental scattering function in $FAPbBr_3$.** Due to high symmetry, to improve signal-to-noise ratio, each image was obtained by averaging the intensities across HKn, nKL and HnL planes.

We observed that prolonged exposure to X-ray synchrotron radiation of $MAPbBr_3$ in the average cubic phase induces a transformation of the local out-of-phase octahedral tilting to in-phase, thus effectively changing the symmetry of the local structure from $I4/mcm$ to $P4/mbm$, whilst maintaining the same twin operators, as demonstrated in Supplementary Figure 33. It is important to note that this occurs without the significant loss of intensity of Bragg peaks, which is a common observation under beam damage. With further exposure, additional reflections appear in the majority of reciprocal space planes, accompanied by a flower-like diffuse scattering in the $HK1.5$ planes in both $MAPbBr_3$ (Supplementary Figure 33 **e** and **f**) and $FAPbBr_3$ (Supplementary Figure 34). Our observation is consistent with previous reports which suggested both electron and X-ray beam damage could lead to the appearance of superstructure reflections [34, 35].

14. INELASTIC NEUTRON SCATTERING

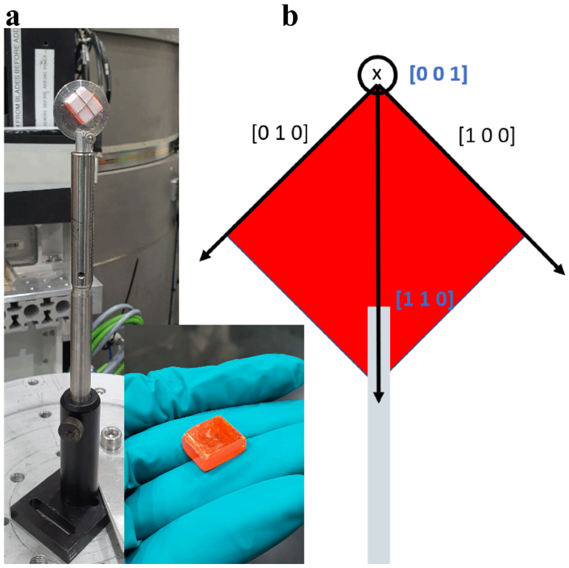

Supplementary Figure 35: **$MAPbBr_3$ single crystal mounting for inelastic neutron spectroscopy experiments.** (**a**) The crystal was mounted diagonally to access the HHL plane. (**b**) Schematic of the crystal orientation, where the crystallographic directions denoted in blue define the scattering plane accessible by the triple-axis spectrometer.

To precisely measure the dynamics of nanodomains, it is necessary to perform quasi-elastic neutron scattering at the R and M points in $MAPbBr_3$ and $FAPbBr_3$, respectively. For this purpose, we selected the cold triple-axis spectrometer Sika, which provides approximately one order of magnitude better energy resolution compared to the thermal triple-axis spectrometer Taipan. To determine the resolution, we performed a variable energy transfer scan using a vanadium standard sample, as presented in Supplementary Figure 36. The full-width at half-maximum (FWHM) of the Gaussian peak was found to be 0.195 meV. Using the relationship $\tau = \hbar/HWHM$, where $HWHM$ is the half-width at half-maximum [36], we determined that the maximum measurable lifetime is 3.37 ps. The dynamics slower than 3.37 ps will be indistinguishable from the instrument response.

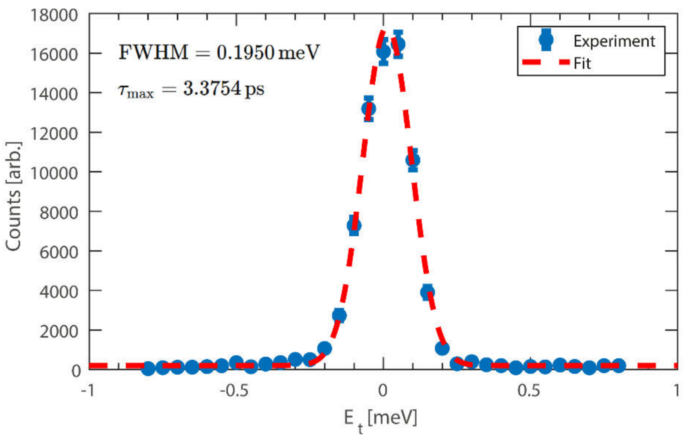

Supplementary Figure 36: **Variable energy transfer scan of vanadium standard sample to determine the instrument response function of cold triple axis spectrometer, Sika.** Data is presented as $\bar{n} \pm \sqrt{n}$, where $\bar{n}$ denotes the mean and $\sqrt{n}$ (depicted with error bars) represents the Poisson counting error for n detected neutrons.

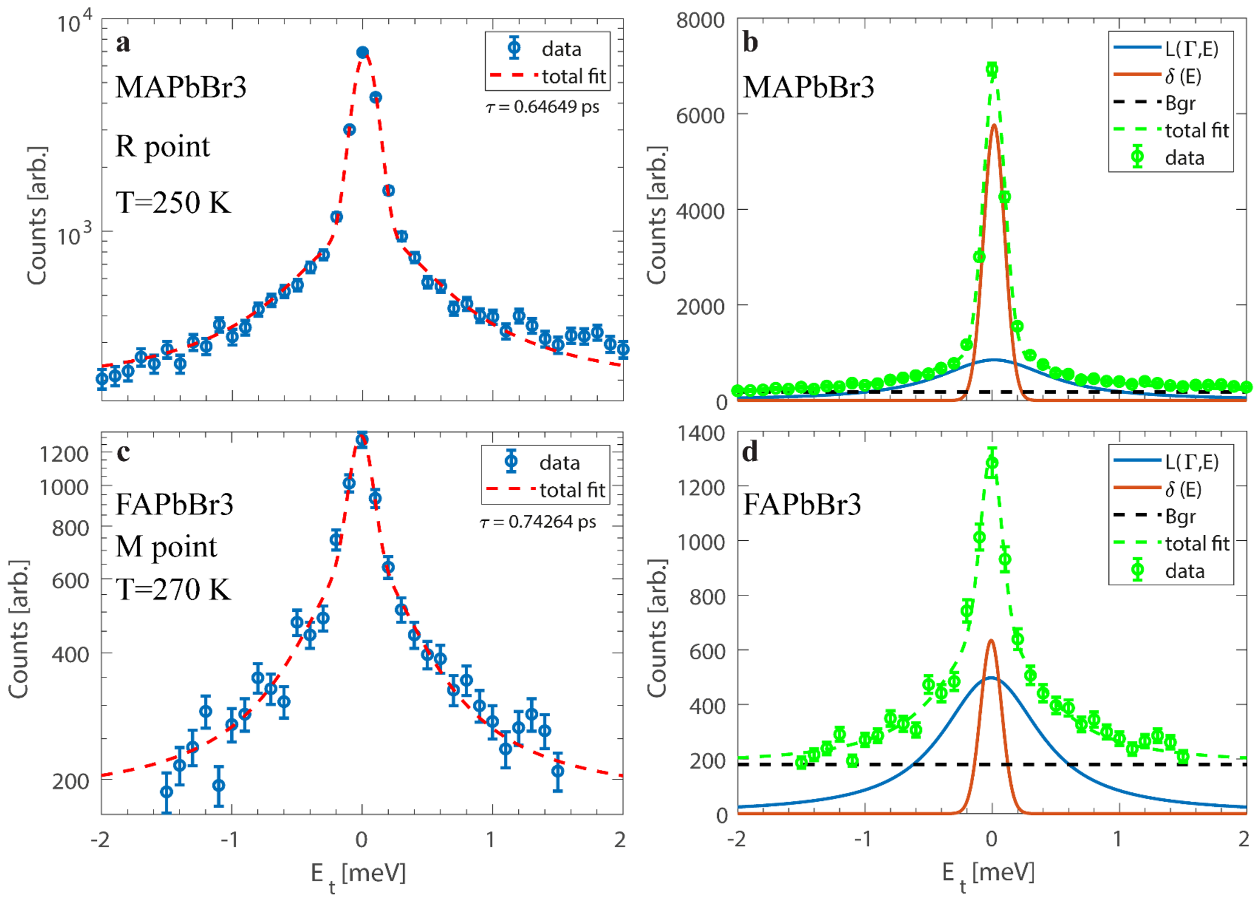

Supplementary Figure 37: **Examples of fitting of QENS data collected with Sika.** **a** Experimental QENS data and the corresponding fit for $MAPbBr_3$ at the R point $q = (-0.5, -0.5, 1.5)$ at $T = 250$ K, presented on a logarithmic scale. The lifetime of the wobble motion of the MA cation is indicated below the legend. **b** The same experimental data with the components from Eq. (S12) convolved with the instrument response function. The Lorentzian associated with flip motions of the A-site cation was not included in the fit. **c** Experimental QENS data and the corresponding fit for $FAPbBr_3$ at the M point $q = (0.5, 0.5, 2)$ at $T = 270$ K. The lifetime of the wobble motion of the FA cation is indicated below the legend. **d** The same experimental data with the components from Eq. (S12) convolved with the instrument response function. The Lorentzian associated with flip motions of the A-site cation was not included in the fit. Data is presented as $\bar{n} \pm \sqrt{n}$, where $\bar{n}$ denotes the mean and $\sqrt{n}$ (depicted with error bars) represents the Poisson counting error for n detected neutrons.

In an attempt to determine the lifetimes of dynamic nanodomains, we performed quasi-elastic scans using a cold triple-axis spectrometer, Sika. As we have outlined in the main text, the R point scattering in $MAPbBr_3$ in the average cubic phase carries information about dynamic tetragonal nanodomains which exhibit out-of-phase correlations of c-axis octahedral tilts along the c crystallographic axis. We fix our q point to the R point in $MAPbBr_3$ but perform a variable energy transfer scan in the quasi-elastic energy window. We repeat the scan for multiple temperatures as we cool down the sample to approach the average tetragonal phase as shown in Supplementary Figure 38 **a**. In Supplementary Figure 38 **b** we repeat the same measurement in $FAPbBr_3$ crystals at M points to capture in-phase dynamic octahedral tilts that are present in this material.

To extract lifetimes, this data must be fitted to an appropriate model. Quasi-elastic neutron scattering contains both incoherent and coherent contributions:

$$S(Q, E) = S_{\text{incoh}}(Q, E) + S_{\text{coh}}(Q, E)\,.$$

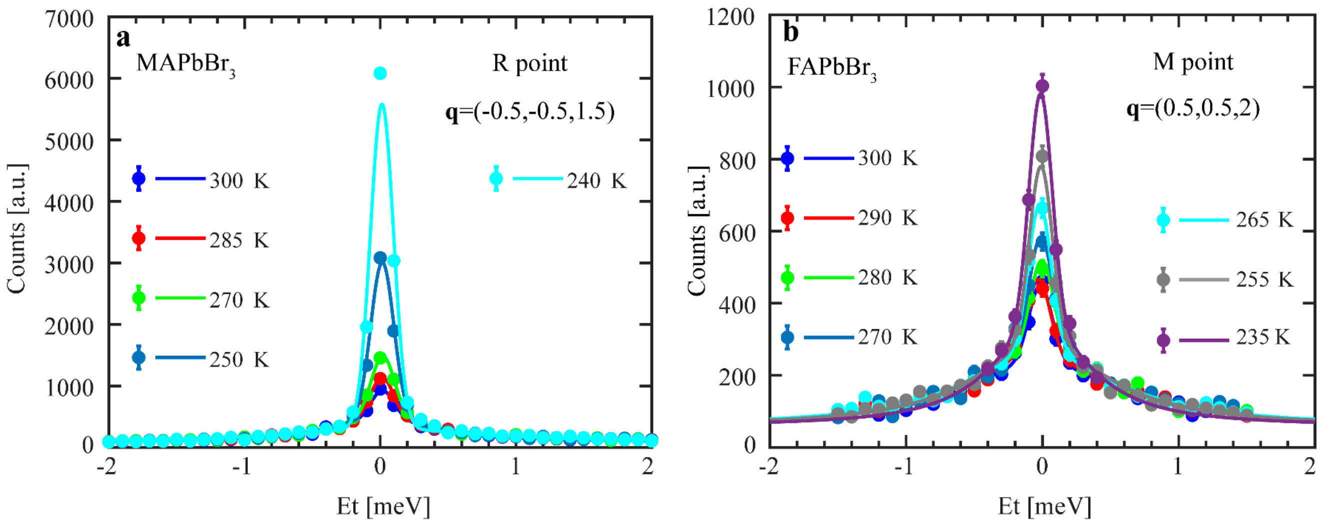

Supplementary Figure 38: **Constant q, variable energy scans of the quasielastic spectrum at the zone edge obtained using Sika.** (**a**) Temperature dependence of $MAPbBr_3$ quasi-elastic spectrum at R point. (**b**) Temperature dependence of $FAPbBr_3$ quasielastic spectrum at M point. Solid lines are best fits to the experimental data. Data is presented as $\bar{n} \pm \sqrt{n}$, where $\bar{n}$ denotes the mean and $\sqrt{n}$ (depicted with error bars) represents the Poisson counting error for n detected neutrons.

Incoherent scattering provides insights into stochastic processes, such as stochastic flips or reorientations of A-site cations in halide perovskites, or the diffusion of atoms. Coherent quasi-elastic scattering, on the other hand, can originate from temporally correlated short-range order or, in the case of halide perovskites, dynamic nanodomains. When hydrogen-containing molecules are present, incoherent scattering becomes order of magnitude stronger due to the very high incoherent scattering cross-section of hydrogen (e.g. incoherent scattering cross section for H is 80.27 barns compared to coherent scattering cross section of e.g. 5.8 for Br). Since our samples are not deuterated, we are subject to a strong incoherent scattering contribution from hydrogen atoms, which may overwhelm the coherent elastic scattering contribution from dynamic nanodomains. For halide perovskites, the incoherent quasi-elastic neutron scattering (QENS) can be expressed as:

$$S_{\text{incoh}}(Q,E) = \{A_{\Delta}\delta(E) + A_{\text{flip}}L[\Gamma(Q),E] + A_{\text{wobble}}L[\Gamma(Q),E]\} \otimes R(Q,E) + \text{Bgr}\,, \tag{S12}$$

where $\delta(E)$ captures the purely static (elastic) contributions, and $A_{\text{flip}}L[\Gamma(Q),E]$ and $A_{\text{wobble}}L[\Gamma(Q),E]$ are two Lorentzian terms that describe different stochastic modes of A-site cations in halide perovskites [37]. All of these terms have to be convoluted with $R(Q,E)$ that is instrument resolution function, usually Gaussian. Bgr is a constant background. Dynamic nanodomains contribute to the coherent scattering term, which can be written as:

$$S_{\text{coh}}(Q,E) = A_{\text{dyn}}L[\Gamma(Q),E]\,.$$

We attempted to fit the data using the full model $S(Q,E) = S_{\text{incoh}}(Q,E) + S_{\text{coh}}(Q,E)$. However, we found that the signal is dominated by $S_{\text{incoh}}(Q,E)$, and the data could be adequately fitted with only a single component, $A_{\text{wobble}}L[\Gamma(Q),E]$. The results of the fit are presented in Supplementary Figure 37, showing the corresponding lifetimes of A-site cation wobble motions, which are in excellent agreement with values reported in the literature [37]. The same fits were performed for all measured temperatures using the identical model leading to the consistent conclusions. Thus, we conclude that this broad Lorentzian component overwhelms all other contributions and effectively masks the coherent quasi-elastic neutron scattering signal. The latter, observed as quasi-elastic diffuse scattering (QEDS) peaks in X-ray single-crystal scattering, cannot be precisely resolved in our measurements. Consequently, we are unable to determine the lifetime of the dynamic nanodomains with the current dataset.

Using Sika, we performed elastic (at zero energy transfer) q-resolved scans, which revealed the presence of diffuse scattering peaks at the R and M points in $MAPbBr_3$ and $FAPbBr_3$, as shown in Supplementary Figure 40 **a** and **b**, respectively.

These peaks are effectively coherent quasi-elastic scattering superimposed on a broad incoherent quasi-elastic background. This background is predominantly governed by elastic incoherent scattering, which strongly depends on the Debye-Waller factor and decreases with Q^2 as shown in eq. 4.52 in [38]. To accurately extract correlation lengths, the

correct Q-dependence of this factor must, in principle, be known. However, achieving this is challenging; therefore, we approximated it using a Gaussian function. In the main text we present the analogous scans performed with Taipan in Fig. 3 **a** and **b**. Due to the same problem the extracted correlation lengths which we present in Supplementary Figure 39 may be subject to inaccuracies.

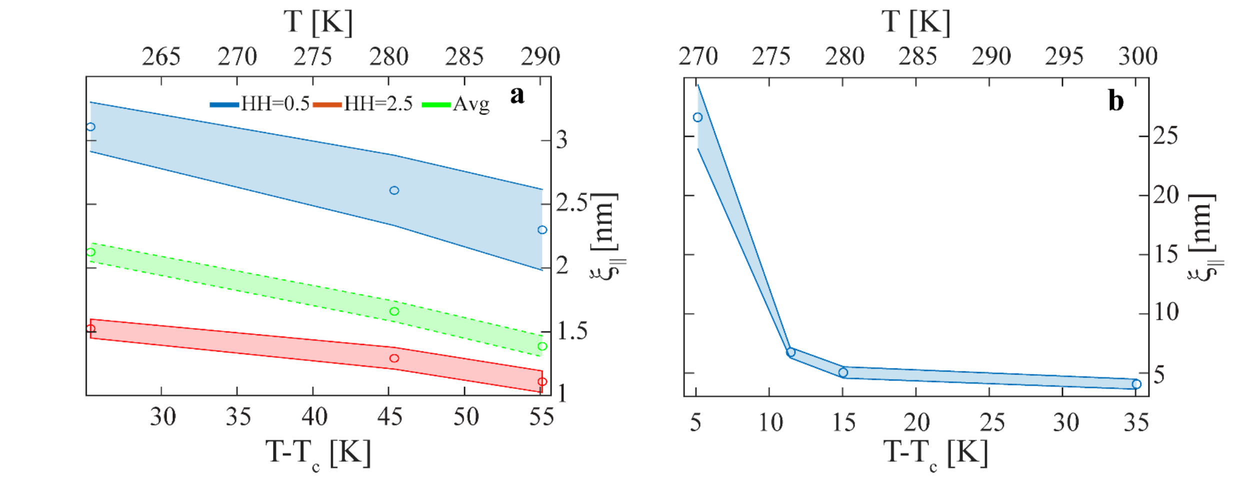

Supplementary Figure 39: (**a**) The correlation lengths obtained by fitting two diffuse scattering peaks at R points of $MAPbBr_3$, $\mathbf{q} = (0.5, 0.5, 1.5)$ and $\mathbf{q} = (2.5, 2.5, 1.5)$, shown in Fig 3. (**a**) of the main text. Their weighted average is depicted by the green curve. (**b**) The derived correlation lengths for the observed diffuse scattering at M point, $\mathbf{q} = (0, 3.5, 0.5)$ for $FAPbBr_3$. In (**a**) and (**b**) the shaded area around the data points represents 95% confidence intervals of the performed fits while T_c denotes the phase transition temperature. The data was collected with a thermal triple-axis spectrometer, Taipan.

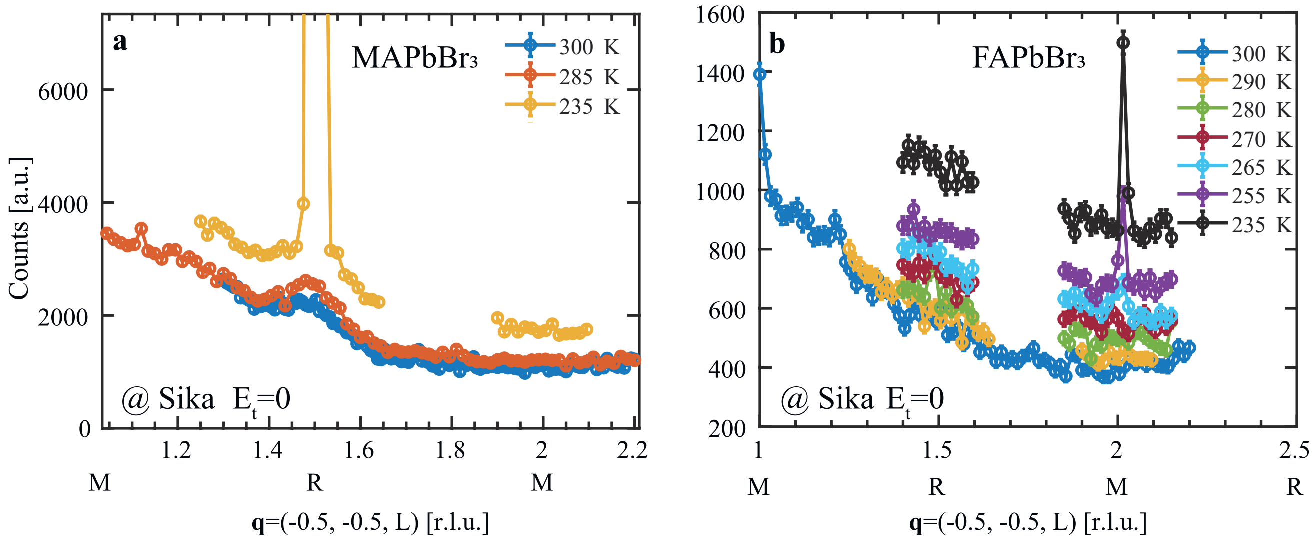

Supplementary Figure 40: **Elastic neutron scattering scans with Sika.** (**a**) Elastic scans (zero neutron energy transfer, $E_t = 0$) across the $[-0.5, -0.5, L]$ direction in the reciprocal space of $MAPbBr_3$ for various temperatures in the average cubic phase. (**b**) Elastic scans (zero neutron energy transfer, $E_t = 0$) across the $[-0.5, -0.5, L]$ direction in the reciprocal space of $FAPbBr_3$ for various temperatures in the average cubic phase. Data is presented as $\bar{n} \pm \sqrt{n}$, where $\bar{n}$ denotes the mean and $\sqrt{n}$ (depicted with error bars) represents the Poisson counting error for n detected neutrons.

To determine the inelastic energy range where scattering from local dynamic nanodomains occurs, we conducted additional scans using Taipan at fixed energy transfers of 1 meV and 2 meV, as seen in Supplementary Figure 41. Neither scan detected diffuse scattering peaks in the inelastic domain, confirming that the local dynamic nanodomain

scattering occurs quasi-elastically within energy windows below 1 meV. The same background from elastic incoherent scattering like with Sika is observed using Taipan at fixed energy transfers of 0 meV.

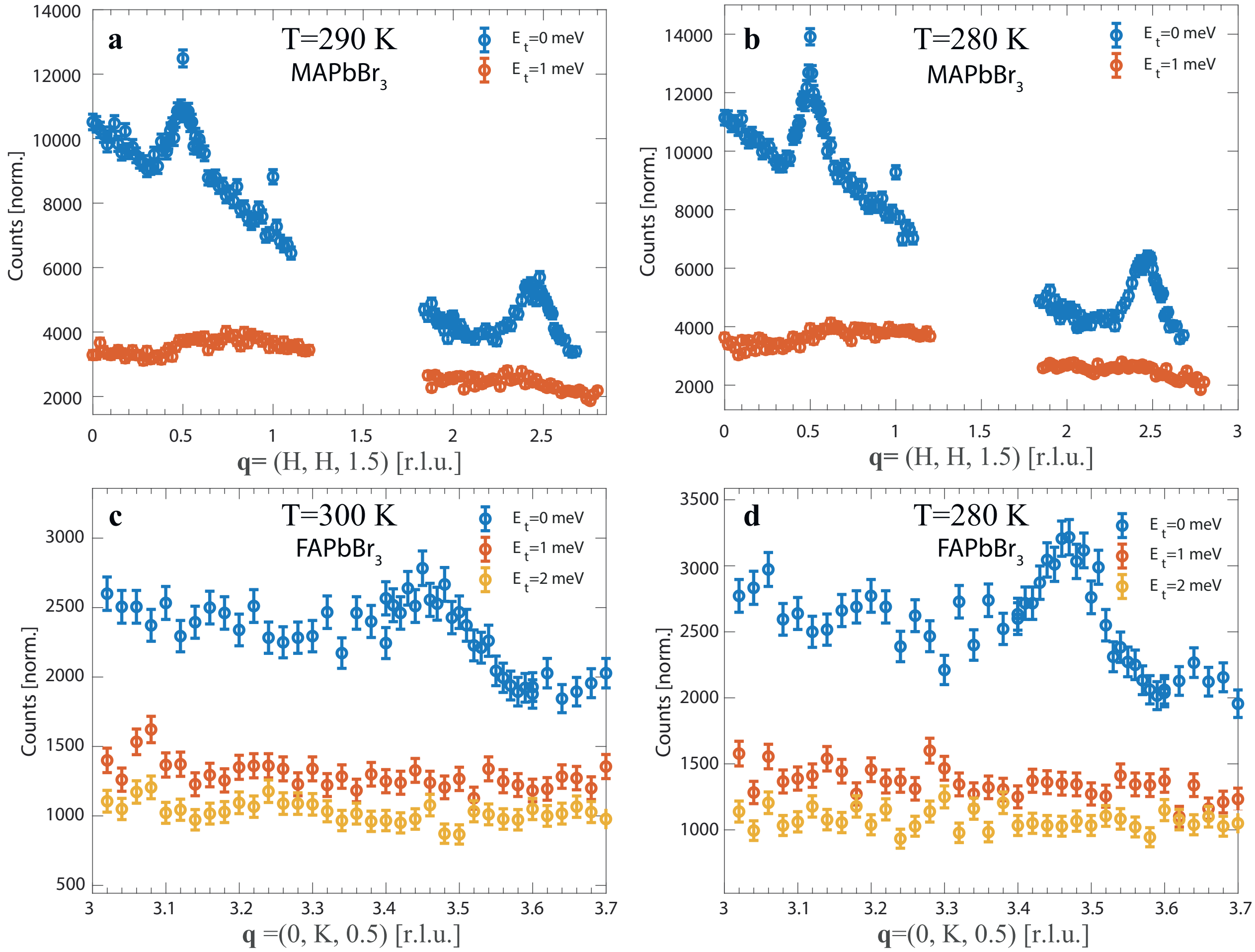

Supplementary Figure 41: **Elastic and inelastic constant energy, variable q scans with Taipan.** (**a**) Elastic ($E_t = 0\,\text{meV}$) and inelastic ($E_t = 1\,\text{meV}$) Q scans across the $[H, H, 1.5]$ direction of $MAPbBr_3$ at $T = 290\,\text{K}$. (**b**) Elastic ($E_t = 0\,\text{meV}$) and inelastic ($E_t = 1\,\text{meV}$) q scans across the $[H, H, 1.5]$ direction of $MAPbBr_3$ at $T = 280\,\text{K}$. (**c**) Elastic ($E_t = 0\,\text{meV}$) and inelastic ($E_t = 1\,\text{meV}, E_t = 2\,\text{meV}$) q scans across the $[0, K, 0.5]$ direction of $FAPbBr_3$ at $T = 300\,\text{K}$. (**d**) Elastic ($E_t = 0\,\text{meV}$) and inelastic ($E_t = 1\,\text{meV}, E_t = 2\,\text{meV}$) q scans across the $[0, K, 0.5]$ direction of $FAPbBr_3$ at $T = 280\,\text{K}$. It is noteworthy that the diffuse scattering peaks in both $MAPbBr_3$ and $FAPbBr_3$ comprise solely elastic scattering, with no inelastic components observed. Data is presented as $\bar{n} \pm \sqrt{n}$, where $\bar{n}$ denotes the mean and $\sqrt{n}$ (depicted with error bars) represents the Poisson counting error for n detected neutrons.

The correlation length (diameter) of the local dynamic nanodomains was calculated using the relationship $\xi = \frac{a}{\pi \cdot \text{HWHM}}$, where $HWHM$ is the half-width half-maximum of the corresponding Lorentzian peak measured in reciprocal lattice units and a is the pseudocubic unit cell [39]. We measured resolution in momentum space for various q points at Taipan and converted it to a maximum detectable correlation length diameter. The results are presented in Supplementary Table X. To conclude, while inelastic neutron scattering does not allow for the precise determination

TABLE X: Real space resolution at various q points at Taipan.

q [r.l.u.]	$[0, 0.5, 0.5]$	$[0, 2.5, 0.5]$	$[0, 3.5, 0.5]$	$[0.5, 0.5, 1.5]$	$[2.5, 2.5, 1.5]$
FWHM [r.l.u.]	0.0192	0.0277	0.0345	0.0135	0.0264
ξ [u.c.]	33.157	22.983	18.4530	47.1570	24.114

of nanodomain lifetimes, we confirm that nanodomains scatter X-rays predominantly quasi-elastically.

15. DIRECT OBSERVATION OF PRESENCE OF FERROELASTIC TWIN DOMAINS IN MAPBBR$_3$ AND THEIR ABSENCE IN FAPBBR$_3$ SINGLE CRYSTALS

Recently, it has been demonstrated that ferroelastic domain walls in halide perovskites can be detected using non-polarized light [40], which differs from the standard technique of using cross-polarized light [41, 42] . The authors propose that in halide perovskites, domain walls form atomically coherent interfaces between twin domains, which share the same composition but exhibit different crystalographic orientations, defined by the mirror twin plane. In materials with low symmetry and optical anisotropy, reflections and refractions may occur due to anisotropy of refractive indices. The optical contrast observed under nonpolarized illumination results from optical reflections and refractions at the domain wall interface, as the refractive indices normal to the interface will differ. We employ the same technique and detect the presence of twin domains in $MAPbBr_3$ and their absence in $FAPbBr_3$. We note that for twins to be easily observable with this method, it is crucial to cleave the crystals to surfaces with very low roughness. Supplementary Figure 43 illustrates the formation of twins when entering the tetragonal phase below 236 K and their

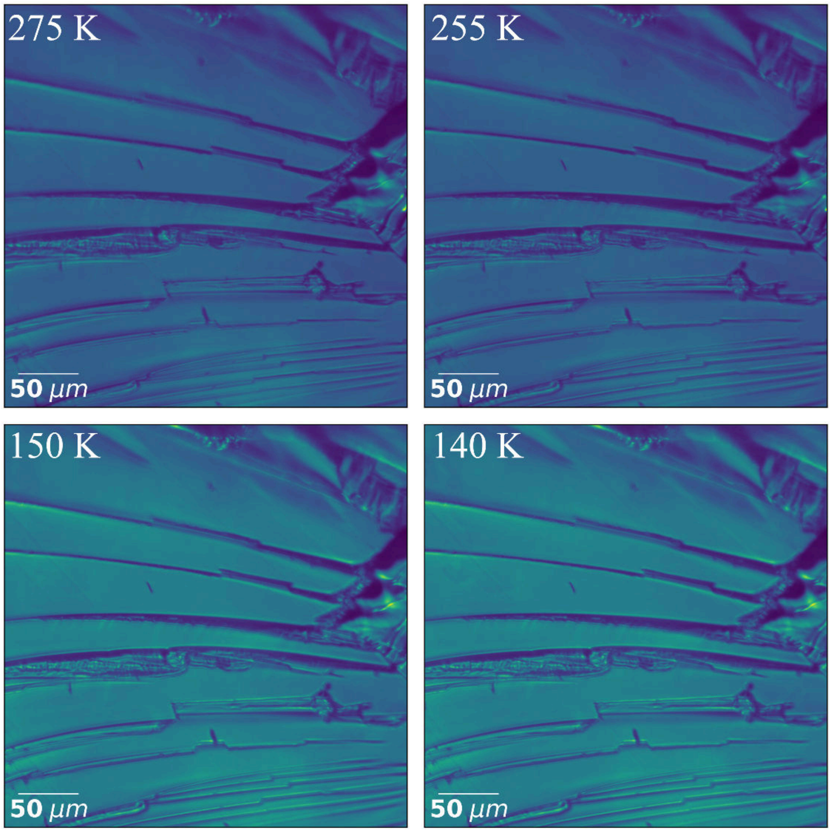

Supplementary Figure 42: **The absence of ferroelastic twin domains in $FAPbBr_3$ was observed using an optical microscope.** Although the system undergoes phase transitions covering the temperature range presented in these images, the formation of ferroelastic twin domains was not observed.

subsequent evolution as the temperature decreases. At T = 200 K, real-space images reveal twin boundaries at a 45-degree angle relative to the principal pseudocubic axes. These boundaries correspond to the (110) twins, which are also identified in reciprocal space through SC-XRD (Supplementary Note 3). The intermediate phase between the cubic and tetragonal phases, previously identified as incommensurate, also results in a distinctive twin domain pattern at T = 150 K, as shown in Supplementary Figure 43. Supplementary Figure 43 **a** further highlights the significant increase in possible twin orientations upon entering the orthorhombic phase at 145 K. This observation aligns with our twinning model for the orthorhombic structure deduced from reciprocal space (Supplementary Note 3), which also predicts the increased degrees of freedom for local structure orientations when transitioning to the orthorhombic phase. Supplementary Figure 43 **a** demonstrates that the formation of twin domains is a macroscopic effect, as it is observable across fields of view (FOVs) spanning several hundreds of microns. On the other hand, we report the absence of ferroelastic twins in Supplementary Figure 43 **b**. We have scanned several fields of view on this and other $FAPbBr_3$ crystals and failed to detect ferroelastic twins in a broad temperature range spanning from 100 K to 300.

This observation is consistent with the existing literature, where ferroelastic twin domain formation has not been reported.

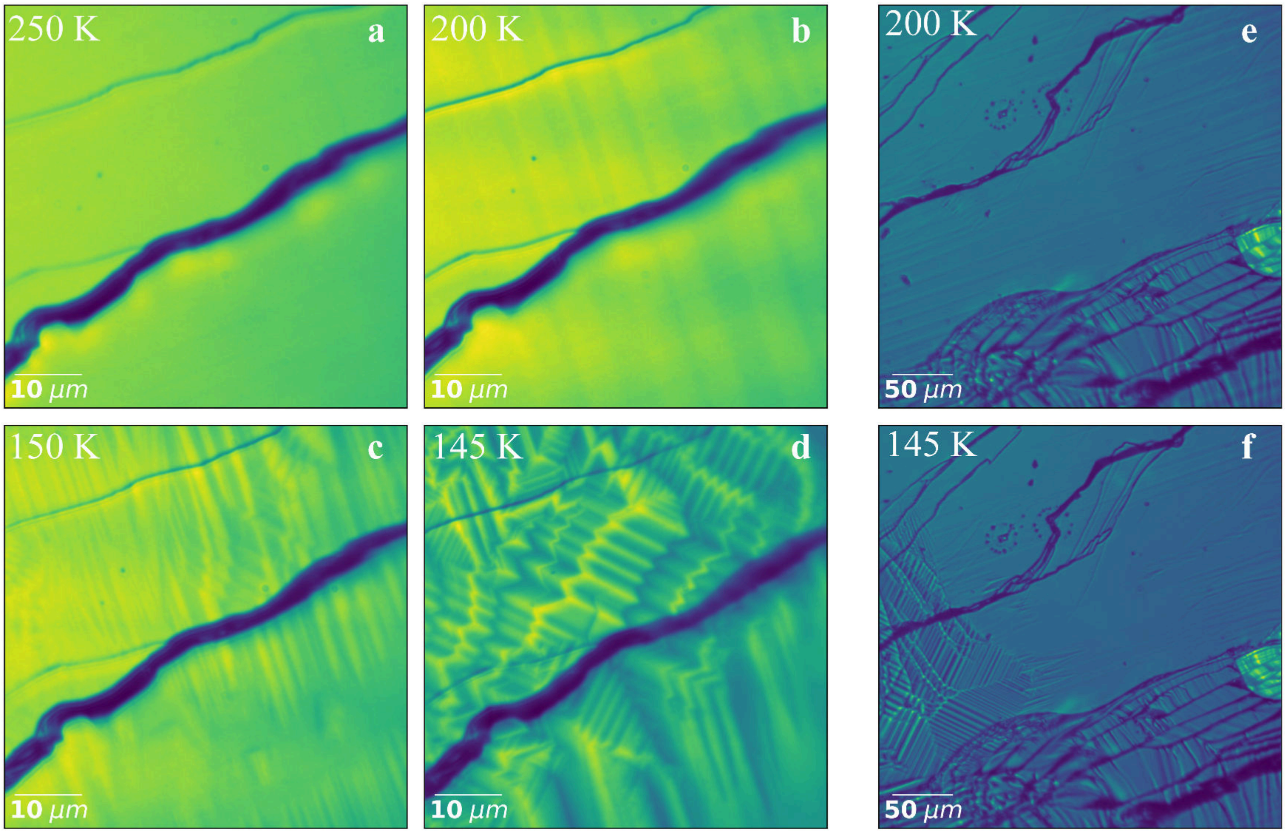

Supplementary Figure 43: **Formation of ferroelastic twin domains in $MAPbBr_3$ was observed using an optical microscope.**(**a**)-(**d**) Diffraction-limited optical images of the same region as a function of temperature. Various twin structures can be observed upon cooling. It is evident that twins are first present at $T = 200\,\mathrm{K}$ (**b**) upon cubic-tetragonal transition and then two types of twins are formed in the orthorhombic phase in (**c**) and (**d**). (**e**) and (**f**) Lower magnification images reveal twin domains in the tetragonal phase at $T = 200\,\mathrm{K}$ and the orthorhombic phase at 145 K.

16. INTERPLAY BETWEEN LOCAL OCTAHEDRAL TILTING AND FERROELASTIC PHASE TRANSTION

The formation of macroscopic twins in $MAPbBr_3$ is observed in optical microscope images of the cleaved single-crystal surface. At 250 K, where $MAPbBr_3$ is in the globally cubic phase (Supplementary Figure 44 **b**), only dynamic local nanodomains are present, with no observable macroscopic twins. However, at 200 K in the average tetragonal phase (Supplementary Figure 44 **e**), the emergence of macroscopic ferroelastic twins is clearly imaged. The twin boundaries are graphically represented in Supplementary Figure 44 **c**, aligning with previous findings in halide perovskites [43, 44]. Moreover, Supplementary Figure 44 **f** illustrates that additional twin types form during the transition from the tetragonal to the orthorhombic phase. The crystallographic relationship between nanodomains of local tetragonal structure in the average cubic phase, as detailed in Supplementary Table I, remains consistent with the global twinning laws observed during the cubic-tetragonal phase transition. These collective observations further reinforce the notion that in $MAPbBr_3$, the emergence of global twinning and ferroelastic phase transition is a direct consequence of the local structure, with the macroscopic twinning governed by symmetry relationships among the local nanodomains.

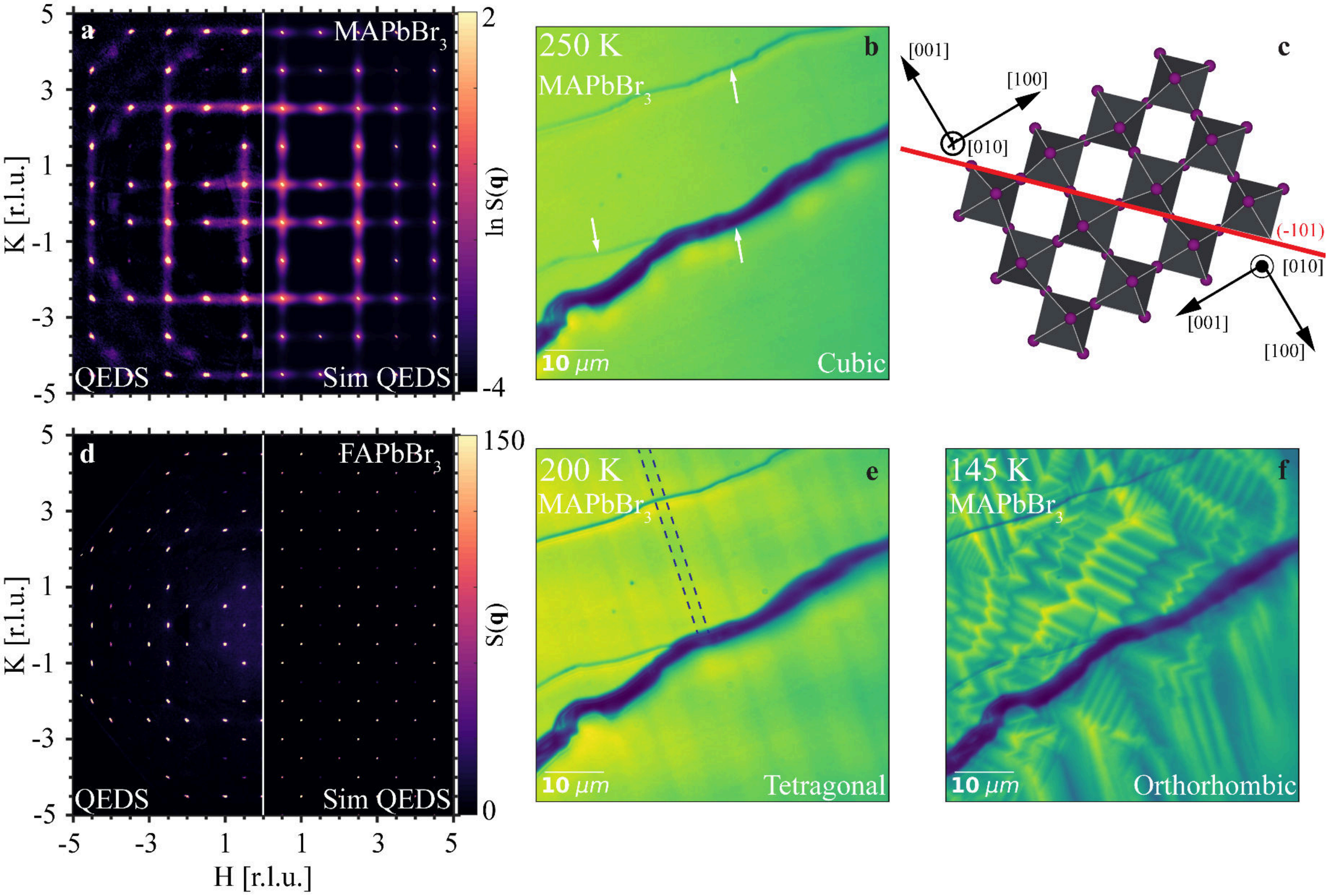

Supplementary Figure 44: **Local structure in the low-temperature phases seeds the macroscopic ferroelastic twins.** (**a**) 2D X-ray $S(\mathbf{q})$ intensity distribution across HK1.5 reciprocal space plane of $MAPbBr_3$ at $T = 200\,K$. Experimental QEDS pattern (left panel) and simulated using the phenomenological model (right panel). Optical microscope image of the cleaved surface of $MAPbBr_3$ in the average (**b**) cubic, (**e**) tetragonal, (**f**) orthorhombic phase. In (**b**) white arrows show crystal cracks and in (**e**), the boundaries of one twin domain are emphasized with dashed blue lines. (**c**) Graphical representation of the two twin components in the tetragonal $MAPbBr_3$ where axes of the two twin components are labelled in the pseudocubic notation, with a clear twin boundary, which we image in (**e**). (**d**) Experimental (left panel) and simulated (right panel) 2D $S(\mathbf{q})$ intensity distribution across HK1.5 reciprocal space plane of $FAPbBr_3$ at $T = 200\,K$, confirming the absence of strong diffuse scattering and no local structure in an average cubic $Im\bar{3}$ symmetry.

We find that $FAPbBr_3$ transitions from cubic $Pm\bar{3}m$ to cubic $Im\bar{3}$ (see further discussion on the assignment of space group in Supplementary Note 12), which is distinct from the commonly suggested $P4/mbm$ space group found in the literature. Under optical microscopy (Supplementary Figure 42) and through crystallographic analysis (Supplementary Figure 32), macroscopic twinning is not detected. This observation is consistent with the lack of reported ferroelasticity in $FAPbBr_3$ [45]. Given that $Im\bar{3}$ forms when each of the three angular components of the octahedra have non-zero and identical tilting angles, it suggests that the suppression of diffuse scattering might result from the disruption of local octahedral correlations when all three components of octahedral angles are locked and exhibit strong infinite correlations in all three directions ($a^+a^+a^+$), as defined by $Im\bar{3}$ space group.

17. OPTOELECTRONIC CHARACTERIZATION OF SINGLE CRYSTALS

The PL full width at half maximum (FWHM) was determined by fitting the PL spectrum to a Gaussian function. To obtain the Urbach energy (E_U) and quasi-Fermi level splitting ($\Delta\mu$) (Supplementary Figure 52), a more robust approach was applied, involving the fitting of the entire PL spectrum $I_{PL}(E)$ to a generalised Planck law derived

from Katahara and Hillhouse [46]:

$$I_{PL}(E) = \frac{2\pi}{h^3c^2} \frac{E^2(1-\exp(-\alpha(E)d))}{\exp\left(\frac{E-\Delta\mu}{kT}\right)-1} \left(1 - \frac{2}{\exp\left(\frac{E-\Delta\mu}{2kT}\right)+1}\right),$$

where E represents the photon energy, h is Planck's constant, and c is the speed of light. The absorption coefficient $\alpha(E)$ is independent of carrier occupation and d is the material's thickness. The denominator $\exp\left(\frac{E-\Delta\mu}{kT}\right)-1$ incorporates the Bose-Einstein distribution, describing the occupation probability of photons at a given energy E. Here, $\Delta\mu$ is the chemical potential difference (or quasi-Fermi level splitting), k is Boltzmann's constant, and T is the temperature. The final term $\left(1 - \frac{2}{\exp\left(\frac{E-\Delta\mu}{2kT}\right)+1}\right)$ arises from the Fermi-Dirac statistics, ensuring proper treatment of carrier occupation probabilities. An appropriate model of the occupation independent absorption coefficient is needed to achieve the full spectrum fitting. Using methods developed by Katahara and Hillhouse [46], the absorption coefficient is modelled using a convolution integral between a tail of subgap states and an idealised (parabolic band) distribution of states above the bandgap:

$$\alpha(\epsilon) = -\frac{\alpha_0\sqrt{\Gamma}}{2\Gamma\left(1+\frac{1}{\Theta}\right)} \int_{-\infty}^{\Delta\epsilon} \exp\left(-\frac{\Delta\epsilon'}{\Theta}\right)\sqrt{\Delta\epsilon - \Delta\epsilon'}\, d\Delta\epsilon',$$

where α_0 is a material-dependent constant, $\Delta\epsilon = (\epsilon - E_g)/\gamma$ is a dimensionless energy relative to the bandgap, γ is a characteristic energy for the width of the subgap tail, Θ modifies the shape of the tail, and Γ is the gamma function. When Θ is set to 1, γ is equal to the Urbach energy E_U. For all fits, we used a value of 5 for the product $\alpha_0 d$, which is a typical parameter for thin films. Consequently, the high-energy side of the spectrum is not perfectly described by this model. This discrepancy arises because our model does not account for photon recycling and PL reabsorption effects which are more dominant in single crystals. Given that information about E_U is contained primarily in the low energy side, this discrepancy will not affect the validity of our results. Alternatively, E_U can be extracted from absorption spectra $\alpha(E)$ estimated from measured PL spectra $\mathrm{PL}(E)$ via the reciprocity theorem:

$$\alpha(E) \propto \frac{\mathrm{PL}(E)}{E^2 \cdot \exp\left(-\frac{E}{kT}\right)},$$

where E is the photon energy, k is the Boltzmann constant, and T is the temperature [47, 48]. We extracted E_u by performing linear fit to the low energy side of $\alpha(E)$ and also by calculating so-called apparent Urbach energy ($E_{U,a}$), defined as the local derivative of the absorption spectra:

$$E_{U,a} = \left(\frac{d\ln(\alpha)}{dE}\right)^{-1}.$$

In Supplementary Figure 48 we demonstrate excellent agreement between E_u extracted from full-spectrum fits, E_u derived from linear fits of estimated absorption spectra from PL, and E_u calculated as the apparent E_u.

The external photoluminescence quantum efficiency (PLQE) is calculated as the ratio of the total emitted photon flux density to the absorbed photon flux density. To determine the total emitted photon flux, we integrate the photoluminescence (PL) spectrum. The absorbed flux density is estimated based on the excitation source parameters, corrected for the sample absorptance, which in the case of thick single crystals is $A = 1 - R$. The laser excitation source delivered 1.39×10^{17} photons/$(\mathrm{s} \cdot \mathrm{cm}^2)$, which is 69% of one sun intensity equivalent photon flux. As we are interested in A at 400 nm, corresponding to the wavelength of our laser, we used a Thorlabs M405LP1 405 nm LED for reflectance measurements. We used a 40× objective (Nikon Plan Fluor ELWD 40x/0.60) for excitation and an Ocean Optics Maya 2000 Pro spectrometer for detection. To determine the local absolute reflectance, we first measured the macroscopic reflectance spectrum of a calibration mirror. The reflected spectrum from the mirror at each point was divided by its macroscopic reflectance spectrum to obtain the calculated incident lamp spectrum. The reflectance data for the sample were then divided by the LED spectrum to determine the sample's absolute reflectance. The temperature-dependent reflectance measurements are shown in Supplementary Figure 51.

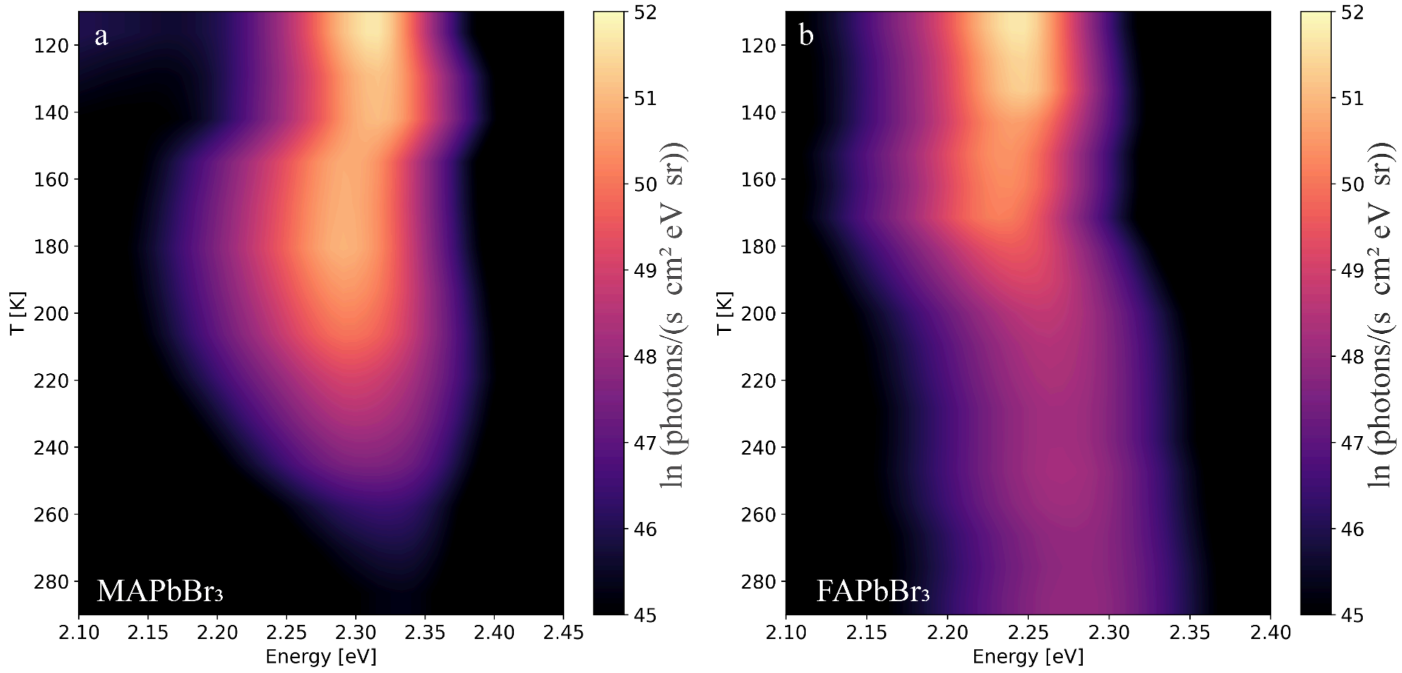

Supplementary Figure 45: **Temperature dependent photoluminescence of $MAPbBr_3$ and $FAPbBr_3$ single crystals.** (**a**) $MAPbBr_3$. (**b**) $FAPbBr_3$. The data is presented in log scale.

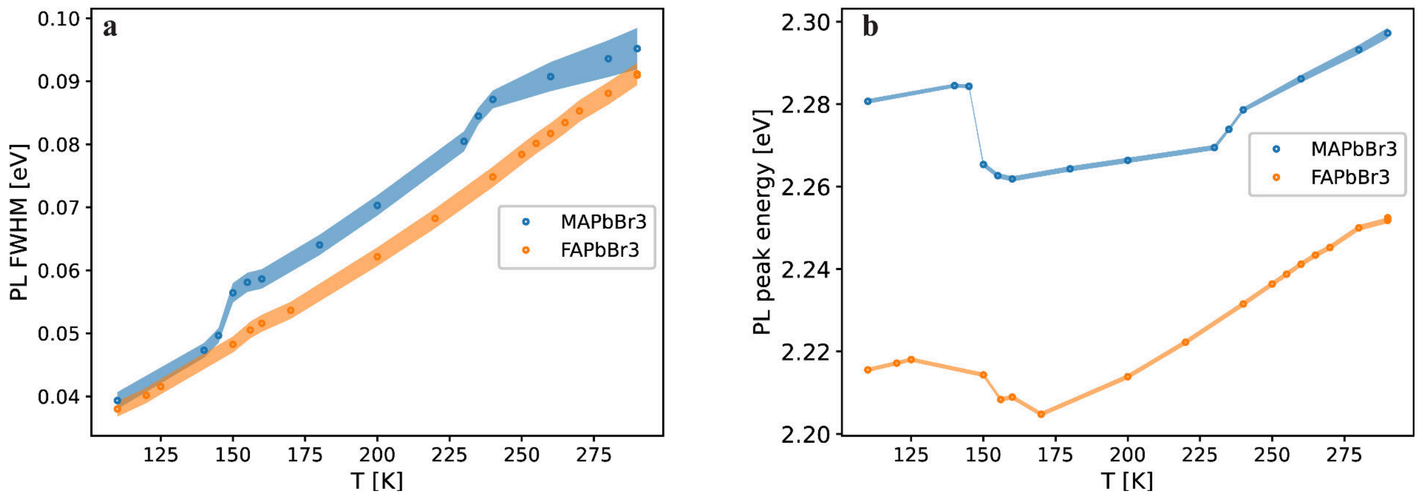

Supplementary Figure 46: **Temperature dependence of fitting parameters obtained by fitting PL spectra to Gaussian function.** (**a**) PL peak energy. (**b**) PL Full Width at Half Maximum (FWHM). The shaded area around the data points represents 95% confidence intervals of the performed fits.

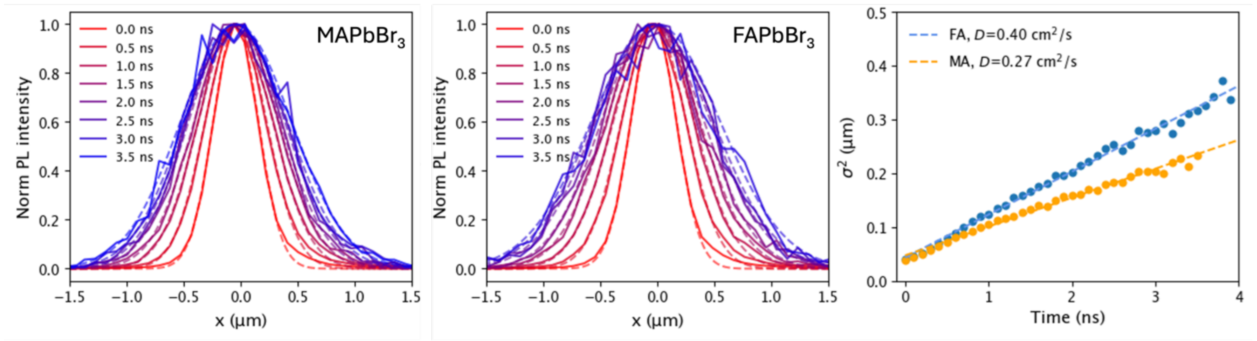

Supplementary Figure 47: **Gaussian fits of the PL profiles at different times and evolution of the squared standard deviation σ^2 of Gaussian fits as a function of time at room temperature.** Diffusion coefficients are found to be 0.40 and 0.27 cm^2/s for $FAPbBr_3$ and $MAPbBr_3$, respectively.

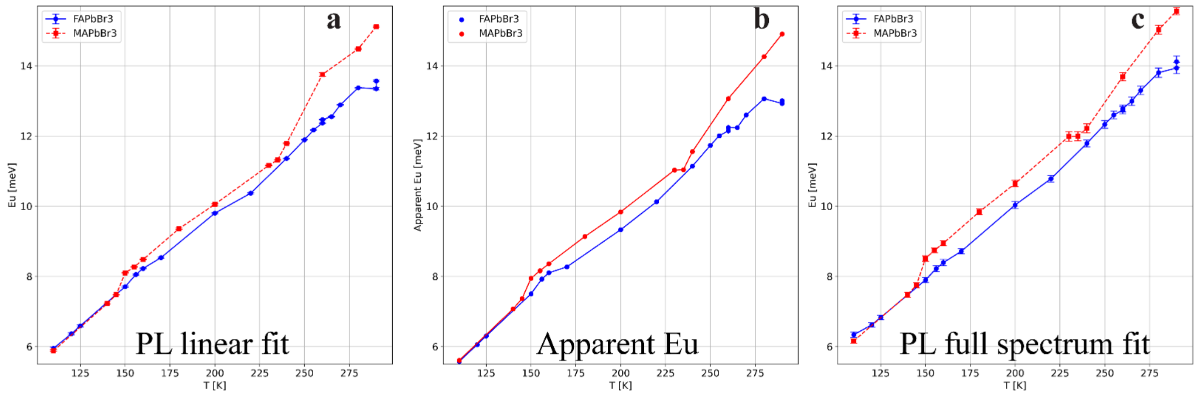

Supplementary Figure 48: **Temperature-dependent Urbach energy (E_u) extracted from the same temperature-dependent PL data using three different methods.** (**a**), By applying a linear fit to the natural logarithm of the absorption coefficient estimated from the PL. (**b**), Using the apparent Urbach energy method. (**c**), By applying a full PL spectrum fit. The length of error bars correspond to 95% confidence intervals. See the Methods section for full details about the different fitting methods.

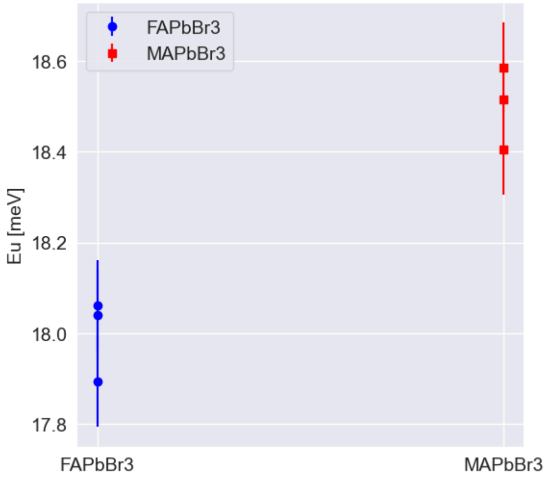

Supplementary Figure 49: **Urbach energy values extracted from PDS measurements.** Each single crystal sample was measured at three different positions. The length of error bars represent 95% confidence intervals of the fits. It is commonly observed that PL-derived Urbach energy is underestimated compared to the PDS method [47, 48], as evidenced by the systematically lower values observed in the PL measurements in Supplementary Figure 48.

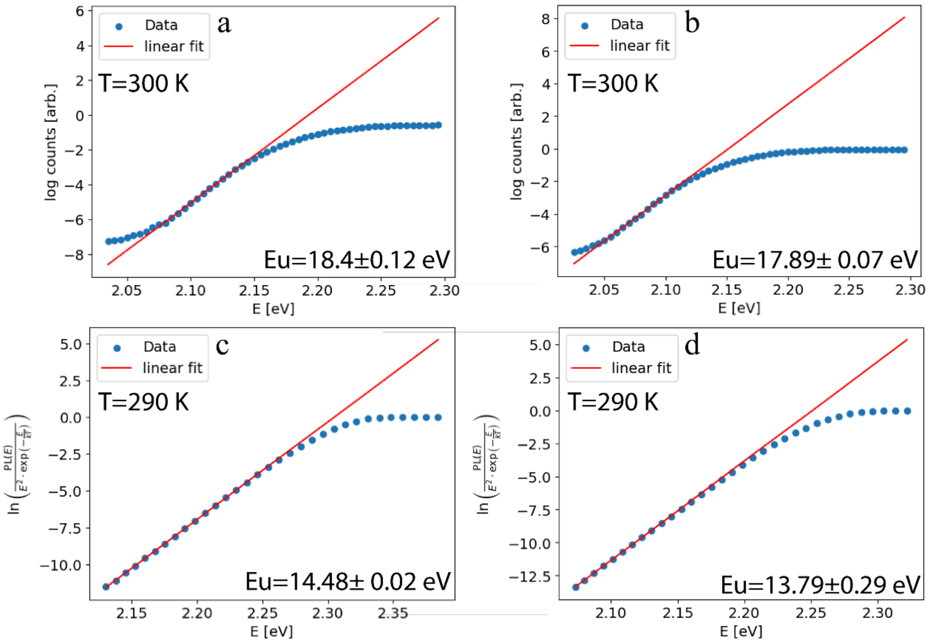

Supplementary Figure 50: **Linear fits performed on PDS and transformed PL data to extract Urbach energy.** PDS data at 300 K with corresponding linear fits and obtained Urbach energy (E_u) values for $MAPbBr_3$ (**a**) and $FAPbBr_3$ (**b**). Estimated absorption coefficient from PL at 290 K with corresponding linear fits and obtained E_u values for $MAPbBr_3$ (**c**) and $FAPbBr_3$ (**d**).

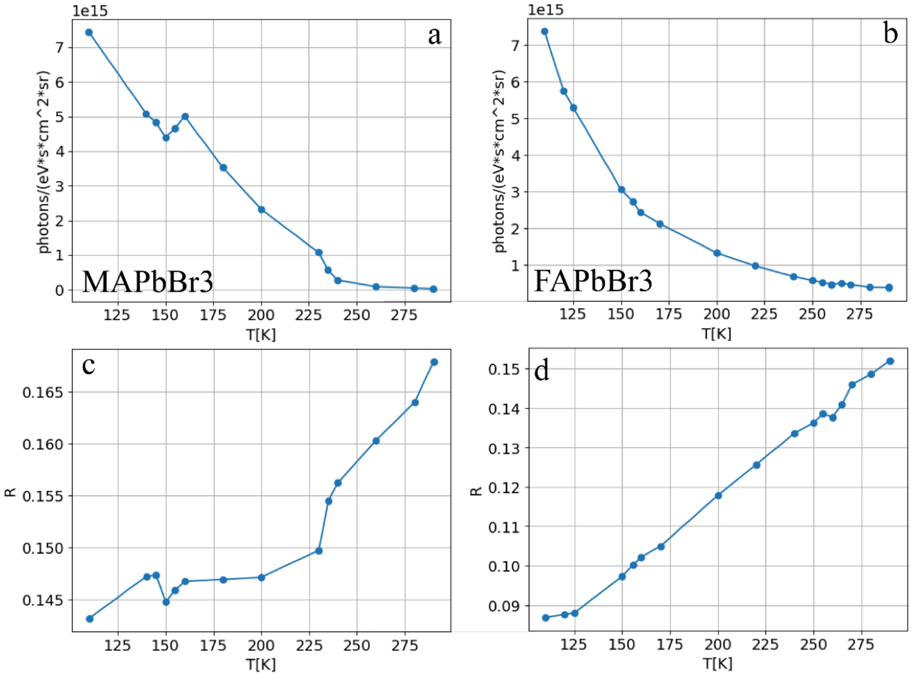

Supplementary Figure 51: **External PLQE measurement.** Panels **a** and **b** display absolute microscopy PL measurements for $MAPbBr_3$ and $FAPbBr_3$, respectively, while panels **c** and **d** show microscopy reflectance measurements for $MAPbBr_3$ and $FAPbBr_3$. These two parameters, along with the known incident laser photon flux, are necessary to determine the PLQE in single crystals.

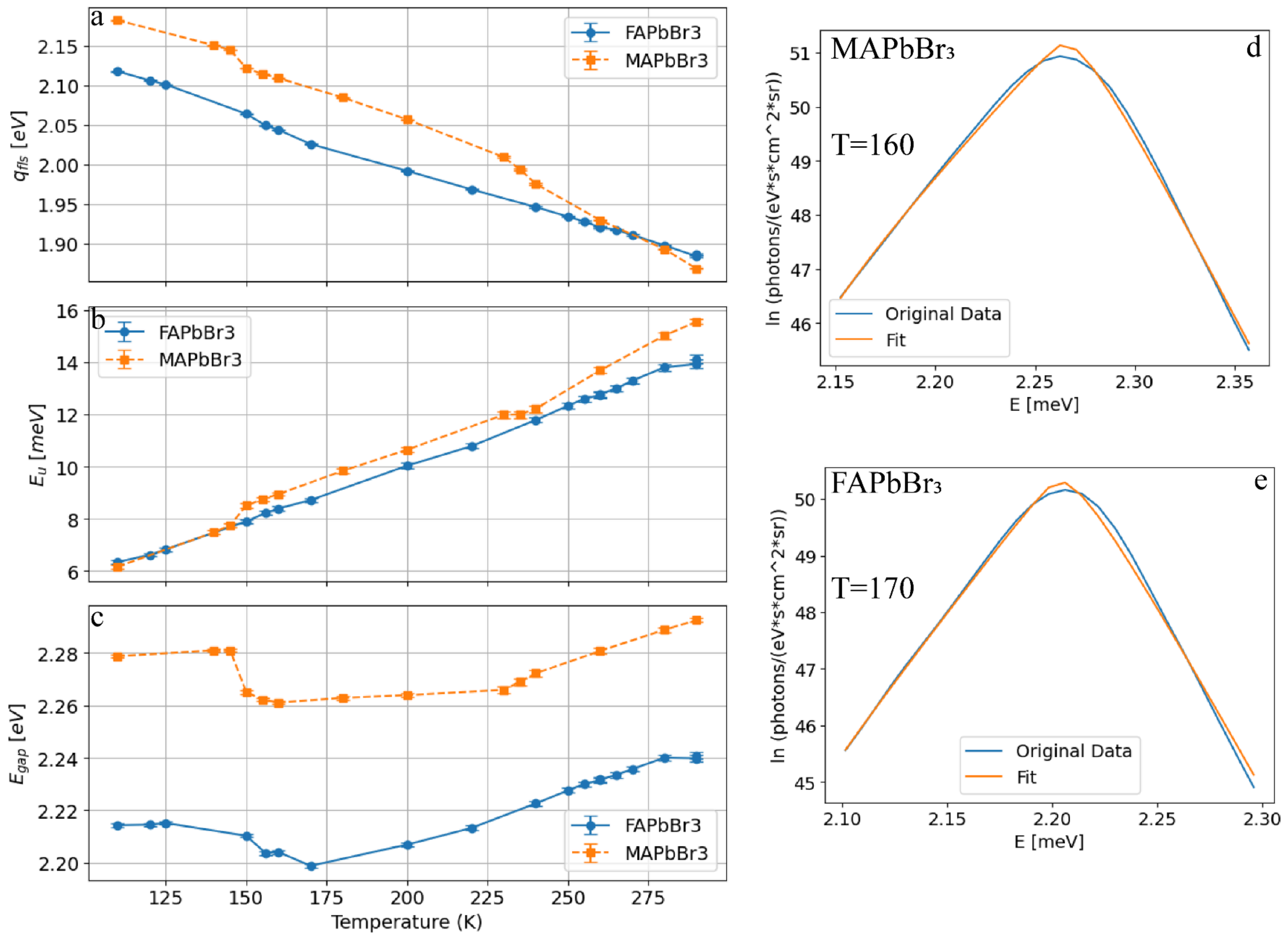

Supplementary Figure 52: **Demonstration of full PL spectrum fitting.** Panels **a**, **b**, and **c** show the extracted quasi-Fermi level splitting (qfls), Urbach energy (E_u), and band gap (E_{gap}) for $MAPbBr_3$ and $FAPbBr_3$ single crystals. Error bars correspond to 95% confidence intervals. Panels **d** and **e** present example PL spectra and the corresponding full spectrum fits for $MAPbBr_3$ at $T = 160\,\text{K}$ and $FAPbBr_3$ at $T = 170\,\text{K}$. The PL data is presented on a logarithmic scale, and R^2 values above 0.99 and relative RMSE below 0.14% (RMSE divided by the mean of the experimental dataset) were achieved for these and all other fits at other temperatures.

[1] Watson, E. S. & O'neill, M. J. Differential microcalorimeter (1966). URL https://patents.google.com/patent/US3263484/en.

[2] Ratto, J., Hatakeyama, T. & Blumstein, R. B. Differential scanning calorimetry investigation of phase transitions in water/ chitosan systems. *Polymer* **36**, 2915–2919 (1995). URL https://www.sciencedirect.com/science/article/pii/003238619594340Y.

[3] Steinmann, W., Walter, S., Beckers, M., Seide, G. & Gries, T. *Thermal Analysis of Phase Transitions and Crystallization in Polymeric Fibers* (IntechOpen, 2013). URL https://www.intechopen.com/chapters/42248. Publication Title: Applications of Calorimetry in a Wide Context - Differential Scanning Calorimetry, Isothermal Titration Calorimetry and Microcalorimetry.

[4] Sun, X., Lee, K. O., Medina, M. A., Chu, Y. & Li, C. Melting temperature and enthalpy variations of phase change materials (PCMs): a differential scanning calorimetry (DSC) analysis. *Phase Transitions* **91**, 667–680 (2018). URL https://doi.org/10.1080/01411594.2018.1469019.

[5] Li, W.-H. *et al.* Dual lattice incommensurabilities and enhanced lattice perfection by low-temperature thermal annealing in photoelectric \$({\mathrm{CH}}_{3}{\mathrm{NH}}_{3})\mathrm{Pb}{\mathrm{Br}}_{3}\$. *Physical Review Materials* **5**, 025401 (2021). URL https://link.aps.org/doi/10.1103/PhysRevMaterials.5.025401.

[6] Guo, Y. *et al.* Interplay between organic cations and inorganic framework and incommensurability in hybrid lead-halide perovskite \${\mathrm{CH}}_{3}{\mathrm{NH}}_{3}{\mathrm{PbBr}}_{3}\$. *Physical Review Materials* **1**, 042401 (2017). URL https://link.aps.org/doi/10.1103/PhysRevMaterials.1.042401.

[7] Wiedemann, D., Breternitz, J., Paley, D. W. & Schorr, S. Hybrid Perovskite at Full Tilt: Structure and Symmetry Relations of the Incommensurately Modulated Phase of Methylammonium Lead Bromide, MAPbBr3. *The Journal of Physical Chemistry Letters* **12**, 2358–2362 (2021). URL https://doi.org/10.1021/acs.jpclett.0c03722.

[8] Gao, Z.-R. *et al.* Ferroelectricity of the Orthorhombic and Tetragonal MAPbBr3 Single Crystal. *The Journal of Physical Chemistry Letters* **10**, 2522–2527 (2019). URL https://doi.org/10.1021/acs.jpclett.9b00776.

[9] Govinda, S. *et al.* Critical Comparison of FAPbX3 and MAPbX3 (X = Br and Cl): How Do They Differ? *The Journal of Physical Chemistry C* **122**, 13758–13766 (2018). URL https://doi.org/10.1021/acs.jpcc.8b00602.

[10] Onoda-Yamamuro, N., Matsuo, T. & Suga, H. Calorimetric and IR spectroscopic studies of phase transitions in methylammonium trihalogenoplumbates (II)†. *Journal of Physics and Chemistry of Solids* **51**, 1383–1395 (1990). URL http://www.sciencedirect.com/science/article/pii/0022369790900217.

[11] Knop, O., Wasylishen, R. E., White, M. A., Cameron, T. S. & Oort, M. J. M. V. Alkylammonium lead halides. Part 2. CH3NH3PbX3 (X = Cl, Br, I) perovskites: cuboctahedral halide cages with isotropic cation reorientation. *Canadian Journal of Chemistry* **68**, 412–422 (1990). URL https://cdnsciencepub.com/doi/abs/10.1139/v90-063.

[12] Sharma, V. K. *et al.* Contrasting Behaviors of FA and MA Cations in APbBr3. *The Journal of Physical Chemistry Letters* **11**, 9669–9679 (2020). URL https://doi.org/10.1021/acs.jpclett.0c02688.

[13] Mozur, E. M. *et al.* Dynamical Phase Transitions and Cation Orientation-Dependent Photoconductivity in CH(NH2)2PbBr3. *ACS Materials Letters* **1**, 260–264 (2019). URL https://doi.org/10.1021/acsmaterialslett.9b00209.

[14] Schueller, E. C. *et al.* Crystal Structure Evolution and Notable Thermal Expansion in Hybrid Perovskites Formamidinium Tin Iodide and Formamidinium Lead Bromide. *Inorganic Chemistry* **57**, 695–701 (2018). URL https://doi.org/10.1021/acs.inorgchem.7b02576.

[15] Keshavarz, M. *et al.* Tracking Structural Phase Transitions in Lead-Halide Perovskites by Means of Thermal Expansion. *Advanced Materials* **31**, 1900521 (2019). URL https://onlinelibrary.wiley.com/doi/abs/10.1002/adma.201900521.

[16] Franz, A., Többens, D. M., Lehmann, F., Kärgell, M. & Schorr, S. The influence of deuteration on the crystal structure of hybrid halide perovskites: a temperature-dependent neutron diffraction study of FAPbBr3. *Acta Crystallographica Section B: Structural Science, Crystal Engineering and Materials* **76**, 267–274 (2020). URL https://journals.iucr.org/b/issues/2020/02/00/ra5076/.

[17] Elbaz, G. A. *et al.* Phonon Speed, Not Scattering, Differentiates Thermal Transport in Lead Halide Perovskites. *Nano Letters* **17**, 5734–5739 (2017). URL https://doi.org/10.1021/acs.nanolett.7b02696.

[18] Agilent Technologies Ltd. Crysalis pro (2014).

[19] Sheldrick, G. M. SHELXT – Integrated space-group and crystal-structure determination. *Acta Crystallographica Section A: Foundations and Advances* **71**, 3–8 (2015). URL http://scripts.iucr.org/cgi-bin/paper?sc5086.

[20] Sheldrick, G. M. Crystal structure refinement with SHELXL. *Acta Crystallographica Section C: Structural Chemistry* **71**, 3–8 (2015). URL http://scripts.iucr.org/cgi-bin/paper?fa3356.

[21] Dolomanov, O. V., Bourhis, L. J., Gildea, R. J., Howard, J. a. K. & Puschmann, H. OLEX2: a complete structure solution, refinement and analysis program. *Journal of Applied Crystallography* **42**, 339–341 (2009). URL //scripts.iucr.org/cgi-bin/paper?kk5042.

[22] Simonov, A. Meerkat: Version 0.3.7. a program for reciprocal space reconstruction. https://github.com/aglie/meerkat (2020). Accessed: 18.12.2023.

[23] Zimmermann, v. M. & Ivashko, O. reconstruction_3d_delta_pdf. https://github.com/4ipsyki/reconstruction_3d_delta_pdf (2023). Accessed: 22.11.2024.

[24] Glazer, A. M. The classification of tilted octahedra in perovskites. *Acta Crystallographica Section B* **28**, 3384–3392 (1972). URL https://onlinelibrary.wiley.com/doi/abs/10.1107/S0567740872007976.

[25] Kielkopf, J. F. New approximation to the Voigt function with applications to spectral-line profile analysis. *JOSA* **63**, 987–995 (1973). URL https://opg.optica.org/josa/abstract.cfm?uri=josa-63-8-987.

[26] Liang, X. *et al.* Structural Dynamics Descriptors for Metal Halide Perovskites. *The Journal of Physical Chemistry C* **127**, 19141–19151 (2023). URL https://doi.org/10.1021/acs.jpcc.3c03377.

[27] Dove, M. T. Theory of displacive phase transitions in minerals. *American Mineralogist* **82**, 213–244 (1997). URL https://doi.org/10.2138/am-1997-3-401.

[28] Weadock, N. J. *et al.* The nature of dynamic local order in CH3NH3PbI3 and CH3NH3PbBr3. *Joule* **7**, 1051–1066 (2023). URL https://www.sciencedirect.com/science/article/pii/S2542435123001290.

[29] Lanigan-Atkins, T. *et al.* Two-dimensional overdamped fluctuations of the soft perovskite lattice in CsPbBr3. *Nature Materials* **20**, 977–983 (2021). URL https://www.nature.com/articles/s41563-021-00947-y.

[30] Cai, S. *et al.* Atomically Resolved Electrically Active Intragrain Interfaces in Perovskite Semiconductors. *Journal of the American Chemical Society* **144**, 1910–1920 (2022). URL https://doi.org/10.1021/jacs.1c12235.

[31] Rothmann, M. U. *et al.* Atomic-scale microstructure of metal halide perovskite. *Science* **370**, eabb5940 (2020). URL https://www.science.org/doi/full/10.1126/science.abb5940.

[32] Coury, F. G., Miller, C., Field, R. & Kaufman, M. On the origin of diffuse intensities in fcc electron diffraction patterns. *Nature* **622**, 742–747 (2023). URL https://www.nature.com/articles/s41586-023-06530-6.

[33] Van Tendeloo, G. & Amelinckx, S. On a simple method to determine the origin of diffuse scattering in electron diffraction patterns. *Scripta Metallurgica* **20**, 335–339 (1986). URL https://www.sciencedirect.com/science/article/pii/0036974886901535.

[34] Rothmann, M. U. *et al.* Structural and Chemical Changes to CH3NH3PbI3 Induced by Electron and Gallium Ion Beams. *Advanced Materials* **30**, 1800629 (2018). URL https://onlinelibrary.wiley.com/doi/abs/10.1002/adma.201800629.

[35] Yang, C.-Q. *et al.* Unveiling the Intrinsic Structure and Intragrain Defects of Organic–Inorganic Hybrid Perovskites by Ultralow Dose Transmission Electron Microscopy. *Advanced Materials* **35**, 2211207 (2023). URL https://onlinelibrary.wiley.com/doi/abs/10.1002/adma.202211207.

[36] Heitler, W. *The Quantum Theory of Radiation* (Courier Corporation, 1984). Google-Books-ID: L7w7UpecbKYC.

[37] Mozur, E. M. & Neilson, J. R. Cation Dynamics in Hybrid Halide Perovskites. *Annual Review of Materials Research* **51**, 269–291 (2021). URL https://www.annualreviews.org/doi/10.1146/annurev-matsci-080819-012808.

[38] Lovesey, S. W. & Lovesey, S. W. *Theory of Neutron Scattering from Condensed Matter: Volume I: Nuclear Scattering*. International Series of Monographs on Physics (Oxford University Press, Oxford, New York, 1986).

[39] Xu, G., Zhong, Z., Hiraka, H. & Shirane, G. Three-dimensional mapping of diffuse scattering in Pb (Zn 1 / 3 Nb 2 / 3) O 3 - x Pb Ti O 3. *Physical Review B* **70**, 174109 (2004). URL https://link.aps.org/doi/10.1103/PhysRevB.70.174109.

[40] Zhang, B. *et al.* Simple Visualization of Universal Ferroelastic Domain Walls in Lead Halide Perovskites. *Advanced Materials* **35**, 2208336 (2023). URL https://onlinelibrary.wiley.com/doi/abs/10.1002/adma.202208336.

[41] Bari, M., Bokov, A. A. & Ye, Z.-G. Ferroelastic Domains and Phase Transitions in Organic-Inorganic Hybrid Perovskite CH3NH3PbBr3. *Journal of Materials Chemistry C* (2021). URL https://pubs.rsc.org/en/content/articlelanding/2021/tc/d0tc05618a.

[42] Strelcov, E. *et al.* CH3NH3PbI3 perovskites: Ferroelasticity revealed. *Science Advances* **3**, e1602165 (2017). URL https://www.science.org/doi/10.1126/sciadv.1602165.

[43] Breternitz, J., Tovar, M. & Schorr, S. Twinning in MAPbI3 at room temperature uncovered through Laue neutron diffraction. *Scientific Reports* **10**, 16613 (2020). URL https://www.nature.com/articles/s41598-020-73487-1.

[44] Rothmann, M. U. *et al.* Direct observation of intrinsic twin domains in tetragonal CH3NH3PbI3. *Nature Communications* **8**, 14547 (2017). URL https://www.nature.com/articles/ncomms14547.

[45] Li, J. *et al.* Ferroelasticity in Organic–Inorganic Hybrid Perovskites. *Chemistry – A European Journal* **28**, e202201005 (2022). URL https://onlinelibrary.wiley.com/doi/abs/10.1002/chem.202201005.

[46] Katahara, J. K. & Hillhouse, H. W. Quasi-Fermi level splitting and sub-bandgap absorptivity from semiconductor photoluminescence. *Journal of Applied Physics* **116**, 173504 (2014). URL https://doi.org/10.1063/1.4898346.

[47] Ugur, E. *et al.* Life on the Urbach Edge. *The Journal of Physical Chemistry Letters* **13**, 7702–7711 (2022). URL https://doi.org/10.1021/acs.jpclett.2c01812.

[48] Witt, C., Schötz, K., Köhler, A. & Panzer, F. Understanding Method-Dependent Differences in Urbach Energies in Halide Perovskites. *The Journal of Physical Chemistry C* **128**, 6336–6345 (2024). URL https://doi.org/10.1021/acs.jpcc.3c07293.
